# Potential of Microalgal Biomass Production in Coastal Deserts for Carbon Dioxide Removal

**DOI:** 10.1021/acs.est.6c08729

**Published:** 2026-08-12

**Authors:** William Leonard, Dale D. McClure, Kok Siew Ng, Aidong Yang

**Affiliations:** † Department of Engineering Science, 6396 University of Oxford, Oxford OX1 3PJ, U.K.; ‡ Department of Chemical Engineering, 3890 Brunel University of London, London UB8 3PH, U.K.

**Keywords:** carbon dioxide removal, microalgal cultivation, direct air capture, biomass burial, biomass energy with carbon capture and storage, air–water CO_2_ mass transfer, technoeconomic assessment

## Abstract

High photosynthetic yields and the ability to grow without fresh water make microalgae interesting mechanisms for carbon dioxide removal (CDR). We characterize two CDR systems which grow microalgae in seawater in coastal deserts—microalgal bioenergy with carbon capture and storage and microalgal biomass burial—and evaluate their potential when using carbon sourced from the atmosphere or ocean to meet CDR objectives via a comparison with direct air carbon capture and storage (DACCS). By deriving and validating a theoretical model of chemically enhanced carbon dioxide mass transfer, we identify the importance of gas–liquid mass transfer into raceway ponds as limiting microalgal productivities and determining technoeconomic viability. Our analysis shows that, while microalgal CDR cannot compete with DACCS in the areal productivity of removals, net removal costs with marine carbon supply are only slightly higher than DACCS with prospects for parity. Though such outcomes may not yet support the use of microalgal biomass for CDR, the carbon supply apparatuses outlined and the theoretical models accompanying them can inform ongoing research into microalgal cultivation without carbon addition for diverse applications.

## Graphical abstract

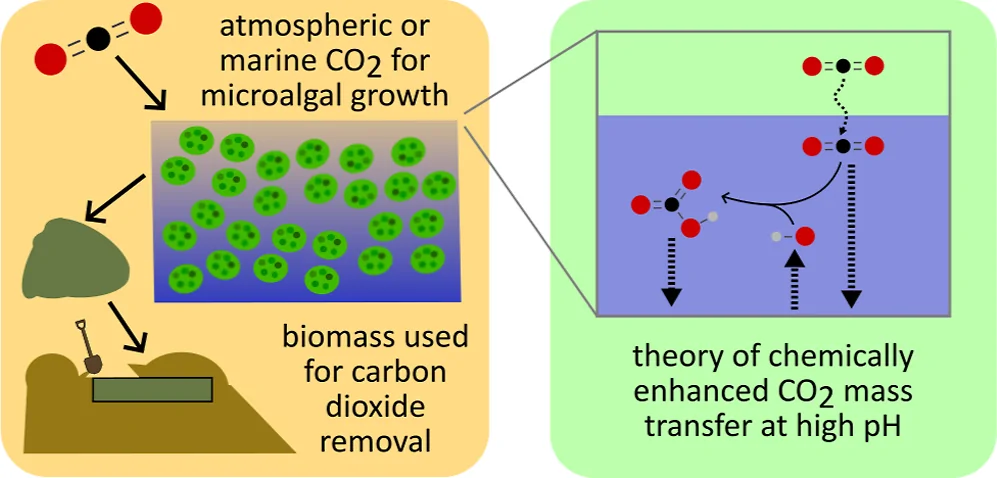

## Introduction

Several billion tons per year of carbon dioxide removal (CDR) are essential for meeting Paris Climate goals of limiting global temperature rise to 1.5 or 2 °C above preindustrial levels by offsetting hard to abate emissions and reducing “legacy” emissions after overshoot.^1^ Guiding CDR accreditation principles from Frontier Climate—a major coordinating body in the voluntary carbon market (VCM)—include durability of stored carbon of greater than 1000 years, nonreliance on arable land, and paths for cost reductions below the loosely defined $100/ton with a capacity of greater than 0.5 gigatons CO_2_/year.^2^ Such objectives generally reflect principles in the literature on both effective climate action^3^ and social sustainability.^4–6^

Currently, the most established CDR strategies are (terrestrial) biomass energy with carbon capture and storage (BECCS, disambiguated herein as tBECCS) and direct air carbon capture and storage (DACCS).^7^ In tBECCS, accumulated biomass is combusted to produce electricity, and released CO_2_ is captured and stored through injection in suitable geological sites. Among novel CDR techniques, tBECCS is the most mature with relatively high levels of technological readiness. However, additional woody biomass plantations required by tBECCS risk competition with agriculture over scarce fresh water and arable land,^8^ while marginal land-cropping and waste biomass are constrained by their limited total scale and highly distributed sourcing.^9^ Meanwhile, DACCS entails the capture of CO_2_ from air by chemical or physical sorbents employing temperature, pressure, or pH swings, yielding compressed CO_2_ which is geologically stored as with BECCS. Primarily consuming only electricity and heat, DACCS has a more straightforward path to scalability. However, DACCS is expensive, with average costs reported in VCMs of $590/ton as opposed to $180/ton for tBECCS^2,10^ ($150–1000 ^11–14^ against $45–200 ^15–18^ in technoeconomic assessments). Biomass burial is a less-mature CDR strategy (though still established in VCMs^19^) of interest to this study: biomass is accumulated and prepared to prevent decomposition—either through high salinity and low water availability,^20^ high acidity, or both^21^—and ultimately encased in a stable natural or synthetic geomembrane to prevent water intrusion for hundreds or thousands of years.^22–24^ Existing comparisons of CDR strategies consider technoeconomics,^25–28^ strategic decision-making,^29–31^ energy efficiency,^32^ scalability,^25,27^ and security^25^ but do not include biomass burial or alternatives to terrestrial biomass-based approaches.

This paper considers the use of microalgae (here referring collectively to photosynthetic eukaryotes and cyanobacteria) for CDR. Marine microalgae, which can photosynthesize more efficiently than plants,^33,34^ fix approximately 50 gigatons of carbon annually through photosynthesis (about half total biosphere fixation), of which hundreds of megatons to multiple gigatons are sequestered for durations of hundreds to tens of thousands of years via the biological carbon pump.^35,36^ Anthropogenic microalgal cultivation, however, is modest in scale and totaled less than 20,000 ton/year biomass in 2023.^37^ Engineered improvements have economized microalgal cultivation by enhancing lipid content^38,39^ and culture growth rate;^33,40,41^ reducing energy costs and chemical consumption of growth,^42,43^ dewatering,^44^ lipid extraction;^45^ and overcoming contamination during industrial-scale cultivation.^46,47^ Meanwhile, bioprospecting of novel cyanobacterial strains have led to dramatic improvements in microalgal growth kinetics.^48–50^ For example, growth rates in outdoor cultivation of nearly 60 g/m^2^/day have been achieved,^44^ double the biofuel industry baseline.^51^

Kim et al.^52^ offer a brief assessment of microalgal CDR by comparing areal productivity of microalgal growth with DACCS, while microalgal biomass energy carbon capture and sequestration (mBECCS),^53^ microalgal biomass burial (mBB),^54,55^ and microalgal biochar production^56^ have specifically been considered as CDR strategies. In all cases, however, quantitative analysis with regard to CDR objectives remains cursory or lacking. Viability of microalgal CDR—along with cultivation for other low or zero carbon applications—depends on carbon supply. While commercial microalgal operations typically supply CO_2_ via sparging allowing for simultaneous pH control, certain studies have suggested the prospects for modest productivities relying upon mass transfer of CO_2_ from the atmosphere into raceways only, a process which may be enhanced by high pH resulting from dissolved inorganic carbon (DIC) depletion from an alkaline growth medium such as seawater.^57–61^

Here, we compare the areal productivity and cost of microalgal CDR strategies with a nonarable land alternative: DACCS. Because of low atmospheric CO_2_ concentration, carbon supply for microalgal CDR is anticipated to be difficult. A natural solution is the reliance on chemically enhanced mass transfer of CO_2_ from air into ponds. Accordingly, we derived and experimentally validated a theoretical model of air–liquid mass transfer in raceway ponds to constrain microalgal growth in such a system. In parallel, we also developed a second carbon supply option to circumvent the gas–liquid mass-transfer bottleneck, which is based on the consumption of DIC supplied from the ocean. Building on physical models of carbon supply in these two options, we performed a technoeconomic assessment of net CDR potential, areal productivity, and cost to allow for comparison of these various CDR strategies and benchmarking against broad CDR objectives. Beyond the prospects for microalgal CDR, we provide insights into the constraints of microalgal cultivation for sustainable applications more generally.

## Methods

### Assessed CDR System Variations

This study examines microalgal CDR through mBECCS (Figure 1a,b) and mBB (Figure 1a,c). We consider mBECCS for its extraction of energy harvested during algal growth—beyond merely carbon capture via photosynthesis—through biomass combustion and electricity generation. Meanwhile, mBB reduces operational complexity, hoping to streamline scaling up of the photosynthetic carbon capture process. At the expense of enthalpy recovery, storage via burial is straightforward—yet durable—and could reduce process costs.

**Figure 1:**
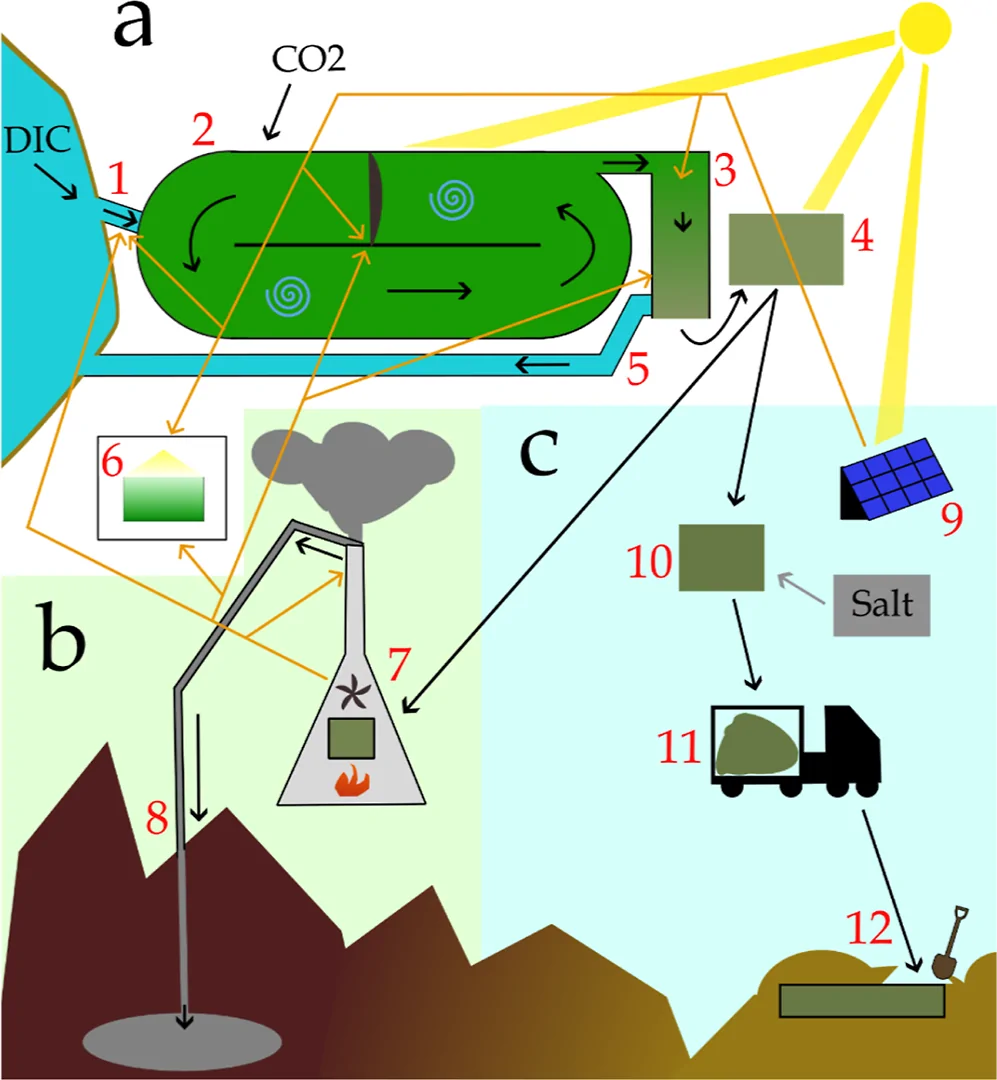
CDR configuration schematics including (a) microalgal biomass production with (1) seawater supply, (2) microalgal raceway pond, (3) biomass sedimentation and dewatering, (4) solar drying, (5) water removal, and (6) photobioreactor; (b) mBECCS equipment with (7) BPP and (8) carbon capture and geological storage; and (c) mBB equipment with (9) solar panel and battery array, (10) salt addition, (11) biomass transport to burial site, and (12) burial. Electricity flows are shown with orange arrows, sunlight is shown in yellow, and black arrows represent carbon fluxes. Note that electricity requirements are provided by the BPP in mBECCS and by photovoltaics in mBB and that marine and atmospheric options are not distinguished.

Microalgae are grown identically in mBECCS and mBB. One or several microalgal strains (which are selected or engineered for halotolerance and growth in local conditions) are grown in raceway ponds built on coastal nonarable land with water and salinity levels maintained by pumping of seawater, and harvested biomass is dewatered and solar-dried (shown to be preferable to thermal drying in Supporting Information, Section S19) to 8% moisture content. For mBECCS, biomass is then transported to a biomass power plant (BPP), where oxy-fuel combustion is performed (favorable to pre- and postcombustion capture in tBECCS technoeconomics^62^). A compression/purification unit (CPU) then captures carbon dioxide from flue gas, which is transported via pipeline to a geological site for injection and storage. Electricity generated powers the air separation unit (ASU) for oxygen production, the CPU, a photobioreactor (PBR, for inoculating raceway ponds), raceway paddle wheels, seawater pumping, and harvesting equipment.

In mBB, after solar drying, the biomass is trucked to a burial site. Burial sites are characterized by extremely low levels of soil moisture and precipitation. Ample salt is added to biomass before it is encased by a polyethylene geomembrane and buried 2–3 m deep in the sand. As mBB fails to generate electricity (necessary to operate equipment on-site), electricity must be supplied.

We consider two possible options for supply of carbon to ponds for microalgal growth. In the *atmospheric* option, ponds are replenished with seawater slowly—only to balance evaporation—to minimize seawater pumping costs and allow access to noncoastal sites. Carbon for growth is derived from mass transfer from the atmosphere into ponds. Meanwhile, in the *marine* option, ponds are replenished rapidly to supply cultures with DIC replete seawater and relieve carbon limitation, at the expense of increased spending on water supply infrastructure (as well as electricity supply to operate it). Water from raceway ponds is pumped to much-enlarged settling ponds (as compared with their sizing when used for harvest alone as in the *atmospheric* option) where biomass settles into a dense phase which is either harvested or recirculated into raceway ponds, leaving a DIC and biomass-depleted supernatant which can be pumped back into the ocean.

mBB and mBECCS were compared against DACCS. A typical low-temperature solid sorbent DAC system with an electricity-driven, heat pump-based temperature swing was assumed for DACCS (Section S1). Off-grid systems—where electricity demand and surplus are provided for and utilized on-site within the considered system—were preferred. In addition to the three CDR strategies and two carbon supply options, several *configurations* were considered representing combinations of power supply and CO_2_ capture. These include three primary configurations, namely DACCS and mBB powered by photovoltaics (PV) with a battery to accommodate intermittency (denoted PV-DACCS and PV-mBB, respectively) and mBECCS coupled with DACCS, the latter utilizing surplus power from the former (mBECCS-DACCS); further configurations, such as mBECCS with electricity sale, are presented in the Supporting Information. Each configuration was assessed for areal productivity and cost of (net) CDR. *Atmospheric* and *marine* options are distinguished as PV-mBB­(*atm*), PV-mBB­(*mar*) etc. For a given configuration, two parametrization *scenarios* were investigated. The *realistic* scenario determined cost and areal productivity with parameters indicative of current technology, whereas the *optimistic* scenario relied on parameters derived from projected technological advancement. Instead of employing technology learning approximations,^63,64^ for the optimistic scenario we individually determined all parameters including efficiencies, costs, embodied emissions, scaling factors, and lifetimes based on novel technologies or technical projections in the literature. Scenario design choices were made to minimize cost before areal productivity.

### Mass-Transfer Prediction and Observation

We derived a theoretical model of chemically enhanced mass transfer of atmospheric CO_2_ into microalgal culture media (see Results section). This model in turn was validated by mass-transfer measurement in bubble columns. High alkalinity media depleted of DIC (representative of complete DIC consumption by microalgae) was bubbled with air and pH was regularly measured. DIC concentration at each time point was determined from a pH-DIC calibration curve corresponding to the constant total alkalinity (TA) used in the bubble column experiments. Further description of this methodology is given in Section S15. Given that no DIC source or sink other than mass transfer was present (media lacked divalent cations to avoid precipitation of Mg­(OH)_2_ and CaCO_3_ which would consume TA and DIC), changes in DIC concentration equaled mass transfer. Media had a TA of 5.0 mM, and ion molarities of 589 mM Na^+^, 546 mM Cl^–^, 23.8 mM SO_4_^2–^, 10.2 mM K^+^, 100 μM total borate, 32 μM NO_3_^–^, and 2 μM total phosphate for a total salinity of 35.6 g/kg (the absence of divalent cations, leads to a slightly lower ionic strength than normal seawater at this salinity). Experiments were conducted at 30 °C.

### Quantifying CDR

All CDR areal productivities and costs were determined from net CDR of a configuration. Throughout, CDR systems were assessed over 1 ha areas. This 1 ha refers to the primary area of sunlight harvest in each configuration, that is the PVs for PV-DACCS and the raceway ponds for mBECCS-DACCS and for PV-mBB. All equipment was assumed to be constructed at an arbitrarily large scale (ignoring coupling efficiencies between subprocesses) before efficiencies and costs were normalized down to the hectare scale. Net removal was calculated considering the removal rate, capacity factor of CO_2_ removing steps (when relevant), and the embodied emissions (or other CO_2_ equivalent radiative forcings) associated with the removal process. Net removal (*R*, ton/ha/yr—unless specified otherwise, tons refer to metric tons of CO_2_) was calculated according to eq 1

1 $$R=G‐GHG_{m,op}-\frac{GHG_{\alpha }+GHG_{m,cons}}{\tau }$$

where *G* refers to gross removals (ton/ha/yr), GHG_m,op_ (ton/ha/yr) and GHG_m,cons_ (ton/ha) refer to embodied greenhouse gas emissions of operation and construction, respectively, GHG_α_ (ton/ha) refers to the CO_2_ equivalent forcing due to albedo change, and τ (yr) is the plant lifetime. The albedo term was calculated via the ∑TDEE methodology over a 100 year period (Section S4). For PV-DACCS gross removal, *G*_PV‑DACCS_ was calculated according to eq 2

2 $$G_{PV‐DACCS}=\frac{\mathrm{I̅}\cdot \eta_{PV}\cdot \left(1-l_{\Psi ,DACCS}\right)}{W_{DAC}}$$

where η_PV_ is the total solar panel efficiency, I̅ is the average daily surface insolation (kWh/m^2^/day), $l_{\Psi ,DACCS}$ is the fractional electricity loss due to curtailment or battery inefficiency, and *W*_DAC_ is the total (specific) electricity consumption of the DAC process (kWh/ton). Values of all nominal analysis constants are provided in Tables 1 and S1–S3. *W*_DAC_ is itself calculated according to eq 3

3 $$W_{DAC}=\Psi_{DAC}+\frac{H_{DAC}}{cop}+W_{comp}$$

where Ψ_DAC_ is the DAC electricity demand (kWh/ton), *H*_DAC_ is the DAC heat demand (kWh/ton), cop is the heat pump coefficient of performance, and *W*_comp_ (kWh/ton) is the energy requirement to compress output CO_2_ to 15 MPa (suitable for transmission to storage).

**Table 1:** Values of Key Technoeconomic Parameters for PV-mBB and mBECCS-DACCS in the Realistic and Optimistic Scenarios^a^

parameter (units)	symbol	real. value	opt. value
PBR/inoculum electricity consumption (kWh/ha/day)	*W*_PBR_	4.5	3
paddlewheel/growth elec. cons. (kWh/ha/day)	*W*_r_	50	40
harvest/dewatering elec. cons. (kWh/ton AFDW)	*W*_h_	14	8
biomass concentration raceway pond (*atmospheric* option, g AFDW/L)	*b*_r_	0.2	0.2
sediment pond output biomass concentration (g AFDW/L)	*b*_set_	10	111
dewatering output moisture (ton H_2_O/ton wet biomass)	*M*_0_	0.8	0.8
seawater DIC concentration (mM)	δ_0_	2.3	2.3
raceway DIC concentration (mM)	δ_r_	0.3	0.3
settling pond frac. biomass in a supernatant	$l_{set}$	0.1	0.05
*atmospheric* option CO_2_ mass transfer (g/m^2^/day)	*J*_r_	3.25	4.9
*marine* option CO_2_ mass transfer (g/m^2^/day)	*J*_r_	3.25	3.25
*atmospheric* algal growth rate (g AFDW/m^2^/day)	*y*_0_	1.77	2.51
*marine* algal growth rate (g AFDW/m^2^/day)	*y*_0_	18	25
biomass HHV (MJ/kg)	HHV	21	23
algal carbon content (g C/g AFDW)	*f*_C_	0.5	0.532
operating days per year	op	330	365
C/P biomass molar ratio	*r*_P_	86	134
C/N biomass molar ratio	*r*_N_	7.3	7.3
ASU elec. cons. (kWh/ton O_2_)	*W*_O_2__	200	137
CPU elec. cons. (kWh/ton)	*W*_CPU_	164	132
other auxiliary power (kWh/kWh gross)	*W*_aux_	0.05	0.03
carbon capture efficiency	η_cc_	0.94	0.97
average insolation (kWh/m^2^/day)	I̅	4.65	5.93
PV efficiency (kWh DC/kWh insolation)	η_DC_	0.22	0.4
total battery/curtailment elec. loss frac. [PV-mBB]	$l_{\Psi }$	0.35	0.35
battery: PV capacity ratio (kWh per kW) [PV-mBB]		2.5	2.6
pipeline distance (km)		165	132
distance to burial site (km)	*d*_T_	15.8	7.5

a A complete list of parameter values (including those presented above) with sources and justifications can be found in Tables S1–S3.

PV-mBB gross productivity *G*_PV‑mBB_ was calculated according to eq 4

4 $$G_{PV‐mBB}=\kappa \cdot Y$$

where κ is the microalgal biomass CO_2_ equivalence (g CO_2_/g ash free dry weight [AFDW]) and *Y* is the annual harvested biomass yield (ton AFDW/ha/yr), which can be converted from daily harvest *y* in g AFDW/m^2^/day via eq 5

5 Y=y·op

where op is the annual fraction of raceway pond operation days. In the *atmospheric* option, *y* is assumed to be dictated by and thus corresponds to the gas–liquid mass transfer of CO_2_ into the raceways *J*_r_ (g/m^2^/day)—with the implicit assumption that carbon supplied by DIC in influent seawater is counterbalanced by carbon lost as DIC or biomass in the effluent. The quantification of *J*_r_ is derived in this work from theoretical prediction validated by experimental observation. Meanwhile, *y* in the *marine* option is calculated according to eq 6

6 $$y=y_{0}-l_{set}\cdot b_{r}\cdot Q_{set}$$

where *y*_0_ is the raw biomass productivity (g AFDW/m^2^/day), dictated by the DIC supply rate through seawater circulation (see eq 8), *Q*_set_ is the flow rate into settling ponds per unit raceway pond (m^3^/ha/day), $l_{set}$ is the fraction of biomass entrained in the supernatant of settling ponds (and thereby lost due to pumping back into the sea), and *b*_r_ is the biomass concentration (g AFDW/L) in the raceway. Note that not all unit conversions are shown. *Q*_set_ is calculated according to the flow rate of water from the sea into the raceway ponds *Q*_in_ (m^3^/ha/day) in eq 7, which is in turn calculated according to the DIC demand necessary to sustain *y*_0_ in eq 8

7 $$Q_{set}=\frac{Q_{in}}{1-\left(1-l_{set}\right)\cdot \frac{b_{r}}{b_{set}}}$$

8 $$Q_{in}=\frac{y_{0}\cdot \kappa -J_{r}}{\delta_{0}-\delta_{r}}$$

where *b*_set_ is the biomass concentration (g AFDW/L) in the settling pond sedimented phase (as opposed to supernatant) and δ_0_ and δ_r_ are the DIC concentrations (mM) in the sea and raceways, respectively.

Similarly, for mBECCS-DACCS gross productivity *G*_mBECCS‑DACCS_ was calculated according to eq 9

9 $$G_{mBECCS‐DACCS}=\kappa \cdot Y\cdot \eta_{cc}+Y\cdot \frac{W_{BPP}-W_{m}}{W_{DAC}}$$

where η_cc_ is the CPU carbon capture efficiency, and *W*_BPP_ and *W*_m_ are the net power generation from the BPP and total power consumption of microalgal growth, respectively (kWh/ton AFDW). *W*_m_ was calculated with eq 10

10 $$W_{m}=\frac{W_{PBR}+W_{r}+W_{p}}{Y}+W_{h}$$

where *W*_PBR_, *W*_r_, and *W*_p_ are energy consumptions (kWh/ha/day) for PBR, raceway paddlewheel, and water supply pumping, respectively, and *W*_h_ is the energy requirement of biomass harvest (from settling ponds until the beginning of solar drying, kWh/ton AFDW). *W*_p_ is calculated according to eq 11

11 $$W_{p}=\frac{Q_{in}\cdot \rho_{sea}\cdot g\cdot h_{p}}{\eta_{pump}}$$

where ρ_sea_ is the water density (kg/m^3^), *g* is the gravity (m/s^2^), *h*_p_ is the total (frictional and elevation) head loss during pumping (m), and η_pump_ is the pump efficiency. Meanwhile, *W*_BPP_ was determined by eqs 12 and 13

12 $$W_{BPP}=HHV\cdot \eta_{comb}\cdot \left(1-W_{aux}\right)-W_{ASU}-\frac{W_{CPU}}{\kappa }$$

13 $$W_{ASU}=Y\cdot W_{O_{2}}\cdot r_{O_{2}}\cdot \left(1+l_{O_{2}}\right)$$

where HHV is the microalgal higher heating value (MJ/kg AFDW), η_comb_ is the gross electrical efficiency of power generation, *W*_aux_ is the fractional auxiliary power consumption (not including ASU and CPU), and *W*_ASU_ and *W*_CPU_ are total ASU and CPU electricity consumptions, respectively (kWh/ton and kWh/ton AFDW, respectively). *W*_O_2__ is the energy consumption of oxygen production (kWh/ton O_2_), *r*_O_2__ is the stoichiometric mass requirement of oxygen for combustion (ton O_2_/ton AFDW), and $l_{O_{2}}$ is the excess oxygen required for combustion.

### Areal CDR Productivity Estimation

For each configuration and scenario, areal productivity was calculated as the net removal divided by the areal footprint of all (significant) land-occupying components of the configuration. Areal productivity η_A_ (ton/ha/yr) was generally calculated according to eq 14

14 $$\eta_{A}=\frac{R}{\sum_{i}\frac{A_{i}}{fr_{i}}}$$

where *A*_0_ ∈ *A*_*i*_ is the primary land (1 ha) occupied by PV panels or raceway ponds to yield net removal *R* (see eq 1). For other *i*, *A*_*i*_ refers to the land requirement of supplemental process *i* (based on the 1 ha primary land use), while *fr*_*i*_ is the filling ratio of process *i* (the fraction of land productively used from a process’ complete footprint). For PV-DACCS, no supplemental processes are required; for microalgae-based configurations solar drying site, settling pond (*marine* configurations only, included in *fr*_0_ for *atmospheric*), and solar panels for electricity supply (PV-mBB only) constitute supplemental land use. Solar drying site land requirement *A*_sd_ was calculated according to eq 15

15 $$A_{sd}=\frac{r_{W}\cdot H_{ev}\cdot y}{\mathrm{I̅}\cdot \eta_{sd}}$$

where *r*_W_ is the mass ratio of water to be evaporated per unit biomass, *H*_ev_ is the energy requirement of evaporation (MJ/kg H_2_O), and η_sd_ is the energy efficiency of solar drying. *r*_W_ was calculated according to eq 16

16 $$r_{W}=\frac{M_{dw}-M_{f}}{\left(1-M_{dw}\right)\cdot \left(1-M_{f}\right)}$$

where *M*_dw_ is the moisture content of biomass before solar drying and *M*_f_ is the final moisture content.

Settling pond area requirement *A*_set_ for mBECCS and mBB in the marine option is calculated according to eq 17

17 $$A_{set}=\frac{Q_{set}\cdot a_{set}\cdot \tau_{set}}{v_{set}}$$

where *a*_set_ and *v*_set_ are the areal footprint (m^2^) and total volume (m^3^) of the settling ponds and τ_set_ is the residence time (hours) of water in the settling ponds to achieve biomass settling corresponding to $l_{set}$ .

For PV-mBB, the supplemental area for solar panels was calculated according to eq 18

18 $$A_{PV,PV‐mBB}=\frac{Y\cdot W_{m}}{\mathrm{I̅}\cdot \eta_{PV}\cdot \left(1-l_{\Psi ,mBB}\right)}$$

where $l_{\Psi ,mBB}$ is the electricity loss rate.

### CDR Cost Estimation and Uncertainty Analysis

The cost of each configuration was calculated by combining technoeconomic data with net CDR. All costs were inflation adjusted to USD 2024. Costs represent a levelized cost of carbon (LCOC) calculated via eq 19

19 $$LCOC=\frac{\left(CapEx-LQ\right)\cdot crf+OpEx}{R}$$

where CapEx is the capital expenditure (in $), OpEx reflects total operating expenses in ($/year), crf is the annuity factor calculated according to eq S8, and LQ is the liquidation value ($) calculated according to eq S9. CapEx includes land purchase, microalgal growth infrastructure, pipelines, PVs, batteries, heat pumps, BPP, CPU, ASU, and DAC plants (Table S2). Due to inconsistent dependence of variable operating expenses on raw productivity or biomass yield, all operating expenses are presented as fixed ($/ha/year). Truly fixed operating expenditures include labor, maintenance, taxes, and insurance (Section S8). Variable operating costs include DAC adsorbent costs; CO_2_ injection cost and burial monitoring cost; BPP ash treatment, water, capture chemicals, and other consumables; macronutrients for microalgal growth (phosphorus and nitrogen); mBB trucking costs; and burial costs of excavation, geomembrane, and salt.

While PV-DACCS and mBECCS-DACCS operating costs are generally derived from literature (Section S8), PV-mBB costs are novel and presented here. Microalgal growth sites entail a photobioreactor for seeding covered and primary raceway ponds, harvesting equipment—that is, sedimentation ponds, membrane filters (realistic only), centrifuges (realistic only), and filter presses (optimistic only)—with capital costs calculated according to Davis et al.,^65^ solar drying site, water supply pipelines (Section S8), and oversized settling ponds for DIC replenishment in the *marine* option (costs adapted from Davis et al. settling ponds). Phosphorus and nitrogen—delivered through diammonium phosphate and ammonia, respectively—costs are calculated according to eqs 20 and 21.

20 $$P_{op}=C_{DAP}\cdot \frac{r_{P}\cdot \left(1-\eta_{P}\right)}{f_{C}}\cdot Y_{0}$$

21 $$N_{op}=C_{{NH}_{3}}\cdot \frac{r_{N}-2\cdot r_{P}\cdot \left(1-\eta_{P}\right)}{f_{C}}\cdot Y_{0}$$

with *N*_op_ and *P*_op_ in $/ton, *C*_DAP_ and *C*_NH_3__ as molar fertilizer costs ($/mol [P,N]), *r*_P_ and *r*_N_ as the P/C and N/C molar ratios, η_P_ as the fraction of phosphorus recovery (nonzero only in optimistic mBECCS where 90% of phosphorus is recovered from bottom ash), and *f*_C_ as the biomass carbon mass fraction related to κ by the ratio of carbon and CO_2_ molar masses, and *Y*_0_ as annual average raw productivity (ton AFDW/ha/year). Trucking costs for mBB were calculated according to eq 22

22 $$B_{T}=c_{crow}\cdot c_{ret}\cdot C_{T}\cdot d_{T}\cdot \frac{Y}{1-M_{f}}$$

where *B*_T_ is the total trucking cost ($/year), *c*_crow_ is the quotient of road distance and Euclidean distance to burial sites, *c*_ret_ is the quotient of roundtrip trucking cost and one way (loaded) transportation cost, *C*_T_ is the baseline trucking cost ($/km/ton), and *d*_T_ (km) is the Euclidean distance from growth to burial sites.

During burial, the site is excavated to a depth *h*_x_ (m). A biomass-salt composite of height *h*_b_ (m) is then placed, encased by a water impermeable geomembrane (here considered only on top and bottom as a simplification for large burial sites), before the excavated soil is replaced on top of the upper geomembrane as a protective layer. Burial salt cost *B*_s_ ($/ton) was calculated according to eq 23

23 $$B_{s}=C_{s}\cdot \frac{f_{s}}{\kappa }$$

where *f*_s_ is the salt requirement during burial (ton salt/ton biomass) and *C*_s_ is the raw salt cost ($/ton salt). Geomembrane cost *B*_geo_ ($/ton) was calculated according to eq 24

24 $$B_{geo}=\frac{2\cdot C_{geo}}{m_{bio}\cdot h_{b}\cdot \kappa }$$

where *C*_geo_ is the geomembrane cost ($/m^2^, doubled for top and bottom) and *m*_bio_ is the partial density of biomass in the burial composite (ton AFDW/m^3^), calculated according to eq 25

25 $$m_{bio}=\frac{\rho_{s}\cdot \rho_{bio}}{\rho_{s}+\rho_{bio}\cdot f_{s}}$$

where ρ_s_ and ρ_bio_ are salt and biomass densities, respectively (ton [salt, AFDW]/m^3^—biomass moisture is assumed to have no effect on volume and so the dry density is used). Finally, excavation cost *B*_x_ ($/ton) is calculated according to eq 26

26 $$B_{x}=\frac{h_{x}\cdot C_{x}}{h_{b}\cdot m_{bio}\cdot \kappa }$$

where *C*_x_ is the excavation cost ($/m^3^ excavated). A burial monitoring cost *B*_M_ ($/ton) is also assessed, while burial site CapEx is neglected.

We performed global sensitivity and uncertainty analyses by selecting upper and lower bounds for selected parameters judged as plausible from the literature for the realistic scenario. Parameters incorporated into the sensitivity and uncertainty quantifications and the ranges for each are given in Table S4. The sensitivity was calculated according to the methodology of Kiparissides et al. (Section S9).^66^ Parameters *l*_set_ and *b*_r_ of microalgal configurations were not included in global sensitivity or uncertainty analyses due to susceptibility of the strategy to become unviable with relatively small perturbations to the two. Cost and CDR viability as functions of variations in these two parameters are instead presented separately.

## Results

### Atmospheric CO_2_ Mass Transfer

Microalgal cultures in both options are assumed to be carbon, as opposed to light, limited. In prospective coastal deserts where microalgal CDR might be implemented, there is sufficient irradiance even on the lowest light days in winter to maintain the biomass productivity in the *marine* option of 18 g AFDW/m^2^/day with a photosynthetic efficiency of 3.7% (see Section S5). While achieving this efficiency in raceway ponds is ambitious, we assume that daily productivities in both options can be met year-round to avoid consideration of variable operation that would follow from colimitation and to maintain a focus on carbon supply pathways. Under carbon limitation, determining microalgal harvest *y* in the *atmospheric* option requires engagement with mass-transfer theory. Mass transfer of atmospheric CO_2_ into seawater is based upon a weak driving force based on equilibration between dissolved CO_2_ at the water surface and its partial pressure in the air according to eq 27

27 $$J_{CO_{2}}=k_{L}\cdot a\cdot \left(\left[CO_{2}^{*}\right]-\left[CO_{2\left(aq\right)}\right]\right)\cdot E$$

where $J_{CO_{2}}$ is the mass-transfer rate (mol/s), *k*_L_ is the gas–liquid mass-transfer coefficient (m/s), *a* is the area (m^2^) of the interface across which mass transfer occurs, [CO_2_^*^] and [CO_2(aq)_]­(mol/m^3^) are the equilibrium CO_2_ concentration as determined by Henry’s law and the actual dissolved CO_2_ concentration, respectively, and *E* is a chemical enhancement factor discussed later. Without chemical enhancement, that is with *E* = 1, mass transfer will only lead to an input of about 1.2 g CO_2_/m^2^/day in a raceway containing seawater fully depleted of CO_2_ at 25 °C, pH 8.1 and with a *k*_L_ of 2.4 × 10^–5^ m/s (pond depth of 0.2 m, fluid velocity of 0.25 m/s). Such mass transfer supports productivity far below baselines for viability in microalgal technoeconomics.^59^ Accordingly, economic viability of microalgal production with atmospheric CO_2_ is contingent on substantial chemical enhancement.

To determine mass-transfer limitations to productivity of microalgal cultures without CO_2_ addition, we offer a theoretical treatment of the specific chemical enhancement involved. The enhancement is based primarily on chemical reactions i and ii, which occur after CO_2_ has been absorbed into the surface layer of seawater (reactions of hydroxide with other alkaline species will have negligible effect due to their low abundance in seawater)

i $$CO_{2}+OH^{-}⇋HCO_{3}^{-}$$

ii $$HCO_{3}^{-}+OH^{-}⇋CO_{3}^{2-}+H_{2}O$$

Though chemical enhancement is negligible for seawater at pH 8.1, when microalgal growth consumes dissolved CO_2_, chemical equilibrium with bicarbonate and carbonate regenerates some dissolved CO_2_, releasing hydroxide. If microalgal growth consumes all DIC, this release of hydroxide ions will raise the pH to greater than 10, hence the potential of noticeable chemical enhancement.

Existing treatments of chemical enhancement lack analytical solutions outside of asymptotic cases in the diffusion or reaction rate-limited regimes. Accordingly, we derive a mass-transfer relationship valid over wide temperature and pH ranges following the method of Hogendoorn et al.^67^ Hogendoorn shows that the (explicit) approximation for enhancement factor with irreversible reaction derived by DeCoursey for surface renewal theory^68^ remains reasonably precise for reversible reactions when the asymptotic enhancement factor of the diffusion-limited regime *E*_a,∞_ is used, even when derived from another mass transfer theory. DeCoursey’s mass transfer equation is shown in eq 28

28 $$E=\frac{-Ha^{2}}{2\left(E_{a,\infty }-1\right)}+\sqrt{\frac{Ha^{4}}{4{\left(E_{a,\infty }-1\right)}^{2}}+\frac{E_{a,\infty }Ha^{2}}{E_{a,\infty }-1}+1}$$

where *Ha* is the Hatta number calculated according to eq 29

29 $$Ha=\sqrt{\frac{k_{1}\cdot D_{Z}\cdot F_{b}}{k_{L}^{2}}}$$

with *k*_1_ (m^3^/mol/s) as the forward rate constant of equation (i), *D*_Z_ as the diffusivity of CO_2_ (m^2^/s), and *F*_b_ as the concentration of OH^–^ in the bulk liquid (mol/m^3^).

Like Hogendoorn, we derive an expression for *E*_a,∞_ in film theory given the work of Olander^69^ with adjustments to the derivation based on our subsequent chemical reactions (Section S12). This yields the explicit expression for *E*_a,∞_ in eq 30

30 $$E_{a,\infty }=1+\frac{K_{1}}{D_{Z}\left(Z_{i}-Z_{b}\right)}\left(Z_{i}\left(D_{X}F_{i}+D_{V}F_{i}^{2}K_{2}\right)-Z_{b}\left(D_{X}F_{b}+D_{V}F_{b}^{2}K_{2}\right)\right)$$

where *K*_1_ and *K*_2_ are the equilibrium constants (m^3^/mol) for the reactions i and ii, respectively, *Z*_i_ and *Z*_b_ as the aqueous CO_2_ concentrations (mol/m^3^) at the interface and in the bulk, respectively, *F*_i_ and *F*_b_ as the interfacial and bulk OH^–^ concentrations (mol/m^3^), and *D*_X_ and *D*_V_ as the diffusivities (m^2^/s) of HCO_3_^–^ and CO_3_^2–^, respectively. Unlike other variables, *F*_i_ cannot easily be measured or assumed but can be determined by solution of a cubic with respect to the above parameters in addition to OH^–^ and H^+^ diffusivities (eq S35). For conditions representative of seawater, we find that *E*_a,∞_ is large relative to *Ha* and accordingly *J* can be well approximated with Hatta’s theory of enhancement based on an irreversible reaction i alone.^70^

Based on such an enhancement factor, we can model instantaneous mass-transfer rate as a function of diffusivities of CO_2_, HCO_3_^–^, CO_3_^2–^, OH^–^, and H^+^, equilibrium constants for reactions i and ii, the forward rate constant for reaction i, bulk concentrations of OH^–^ and CO_2_, an interfacial concentration of CO_2_ via Henry’s law, and a mass-transfer coefficient *k*_L_ (calculated in Sections S13 and S14). These values are in turn expressed in terms of empirical relationships with temperature, salinity, pH, total DIC concentration in the bulk, raceway depth, and fluid velocity. We can then calculate an upper bound for microalgal growth by assuming instantaneous and complete consumption of DIC in 12 daylit hours, evaluating effects of this consumption on seawater chemistry—specifically bulk OH^–^ and CO_2_ concentrations—and then numerically integrating to determine total mass transfer. Importantly, in addition to the carbonate equilibria involved in enhancement factor calculation, increased pH by DIC consumption will also affect equilibria of other alkaline species, namely that of borate naturally existing in seawater and those of phosphate and ammonium added as nutrients. Starting integration with the assumption of complete DIC depletion at dusk for growth media characteristic of microalgal growth in the *atmospheric* option (salinity = 35 g/kg, TA = 2.5 mM, [total borate] = 0.1 mM, phosphate and ammonia depletion, temperature = 30 °C, depth = 20 cm, and fluid velocity = 25 cm/s), microalgal growth during the following day totals to only 4.8 g AFDW/m^2^, with a daytime enhancement factor of 4.58 falling to 4.08 at maximum DIC concentration at dawn. The total microalgal growth decreases to 3.9 g AFDW/m^2^/day when ammonia and total phosphate concentrations are increased even to the modest 0.3 mM and 0.05 mM, respectively, reflecting the inability of microalgae to immediately consume added nutrients, because a smaller fraction of conserved TA exists as hydroxide. Figure 2a shows *E* in seawater as a function of DIC depletion, while Figure 2b,c show the upper bound (mass transfer limit) to *y* at varying fluid velocity and temperature and at varying salinity and TA, respectively.

**Figure 2:**
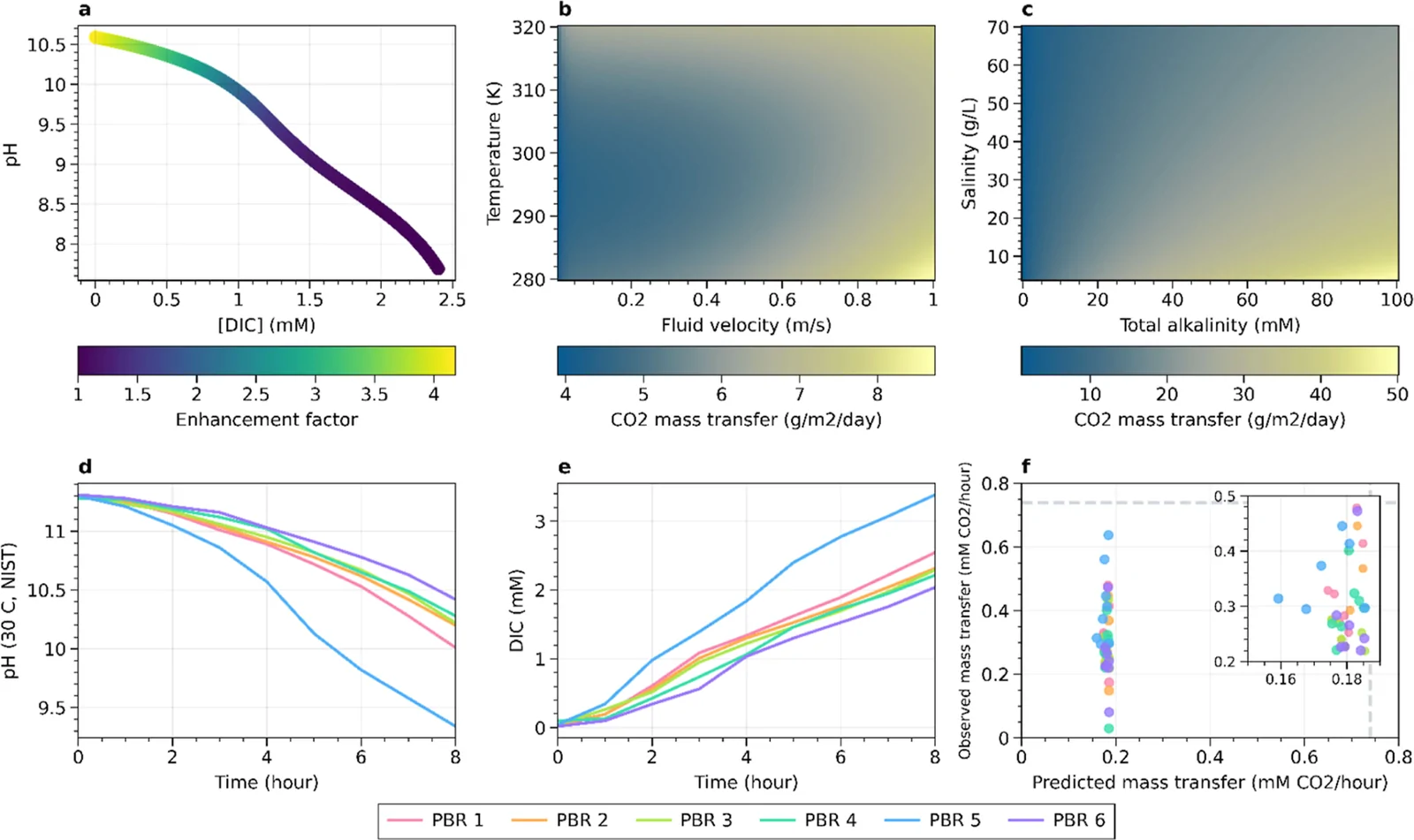
(a) Thermodynamic pH and mass transfer enhancement factor as a function of DIC depletion in seawater (TA = 2.5 mM, [total borate] = 0.1 mM, salinity = 35 g/kg, temperature = 25 °C, depth = 0.2 m, fluid velocity = 0.25 m/s); (b) CO_2_ mass-transfer rate in seawater (as in a) as a function of fluid velocity and temperature; and (c) CO_2_ mass-transfer rate in seawater (as in a) as a function of TA and salinity. Total DIC depletion is assumed, so all alkalinity exists as hydroxide except 0.1 mM borate. Note that the TA range shown is far beyond that of natural seawater, requiring chemical addition; and (d) measured pH and (e) calculated DIC concentration by bubble column over time; and (f) comparison of observed and predicted CO_2_ mass-transfer rates during bubble column experiment, with dashed gray lines showing complete mass transfer of CO_2_ from bubbles.

### Mass Transfer Observation

To validate the conclusion of reaction rate limited chemical enhancement, we observed gas–liquid mass transfer of CO_2_ in bubble columns. Figure 2d,e show measured pH and calculated DIC concentrations in the three bubble columns over time. Figure 2f compares observed mass transfer to a prediction (Section S14) based on correlations for *k*_L_ and *a* for bubble columns and calculation of driving force and enhancement (further refinements based on CO_2_ depletion in bubbles given in Section S16). Predicted mass transfer remained consistent as a driving force and predicted enhancement factor at the bubble interface only varied slightly throughout the experiment. Due to the high *k*_L_ of 7.6 × 10^–4^ m/s at the bubble interface (an order of magnitude higher than in raceway ponds), *E* for DIC depleted seawater with 2.5 mM TA as above is predicted at 1.01 (compared with 4.58 in raceways) and *Ha* vanishes, ensuring reaction rate-limited chemical enhancement. Mass transfer across the headspace in the bubble columns was also calculated, and a lower *k*_L_ of 9.2 × 10^–5^ m/s allowed for a noticeable *E* of 2.05, falling to near 1 at the end of the experiment. While the decreasing headspace enhancement factor explains the slight decrease in predicted mass transfer with falling pH, bubble interface mass transfer still dominated, and this trend is not discerned in a noisy mass-transfer observation. Use of the *k*_L_ independent diffusion limited *E* = *E*_a,∞_ would yield an *E* of 428 at the beginning of the experiment; the reasonable agreement between the theoretical model’s prediction and the experimental data suggests that such diffusion-limited chemical enhancement was not present. While the lower *k*_L_ in raceway ponds may produce more diffusion-limited behavior allowing for greater enhancement than that observed in bubble columns, there was no evidence that *E* even lay in an intermediate regime which might invalidate the theoretical model. An approximate agreement between observed and predicted mass transfer *J* validates the conclusion of extreme carbon limitation in the *atmospheric* option raceway ponds. The theoretical model is further validated by its general reproduction of albeit noisy pH-dependent observations of $J_{CO_{2}}$ by Crowe 2022.^59^

### Areal CDR Productivity

Areal productivity was calculated as the quotient of net CO_2_ removals and the areal footprint of the land-occupying steps of the removal process. The footprints of other components were ignored. Specifically considered were the footprints of photovoltaic arrays, microalgal raceways, settling ponds, and solar drying sites. Energy and carbon flows were then tracked through the balance of each configuration with scenario-dependent energy demands and efficiencies. The embodied emissions of each process from operations, construction, and the radiative forcing of the albedo shift were then evaluated and converted to CO_2_ equivalence for ultimate areal productivity quantification.

Figure 3a–c track solar energy through realistic PV-mBB­(*mar*), mBECCS-DACCS­(*mar*), and PV-DACCS configurations, while Figure 3d–f show the CO_2_ equivalent embodied forcings. In realistic *marine* option microalgal configurations, while PV and drying land footprints are small, settling pond area accounts for 15% and 23% of raceway area in the realistic and optimistic scenarios, respectively. Configurations in the *atmospheric* option are not shown as their embodied emissions exceed gross removals. Even in the optimistic scenario the *atmospheric* option’s low biomass productivity and high footprint-based embodied forcings (raceway construction and albedo) lead to embodied forcings at 78% of gross removals and very low net productivity. Similarly, high footprint forcings in realistic PV-mBB­(*mar*) and mBECCS-DACCS­(*mar*) lead to higher total embodied reductions of 28% and 29% as compared to 16% for PV-DACCS; in the optimistic scenario embodied forcings fall to 13%, 10%, and 7% for PV-mBB­(*mar*), mBECCS-DACCS­(*mar*), and PV-DACCS, respectively. Figure 3g shows the areal productivities of different CDR configurations for both scenarios. In the realistic scenario, PV-DACCS, mBECCS-DACCS­(*mar*), and PV-mBB­(*mar*) garner removals of 832, 33, and 27 ton/ha/year, respectively while these increase to 7539, 202, and 78 ton/ha/year in the optimistic scenario. Efficiencies of mBECCS-DACCS­(*mar*) and PV-mBB­(*mar*) are quite similar in the realistic scenario where mBECCS surplus electricity is small, but mBECCS-DACCS­(*mar*) outpaces PV-mBB­(*mar*) in the optimistic scenario where DAC contributes more than half of gross removals. Meanwhile, PV-DACCS substantially outperforms both PV-mBB­(*mar*) and mBECCS-DACCS­(*mar*) in both scenarios because of the higher energy efficiency of the core DAC capture process where the adsorbent fixes atmospheric CO_2_ and is regenerated as compared to carbon capture via photosynthesis. Optimistic PV-mBB­(*atm*) efficiencies are very low due to low *J*_r_ and high embodied forcings.

**Figure 3:**
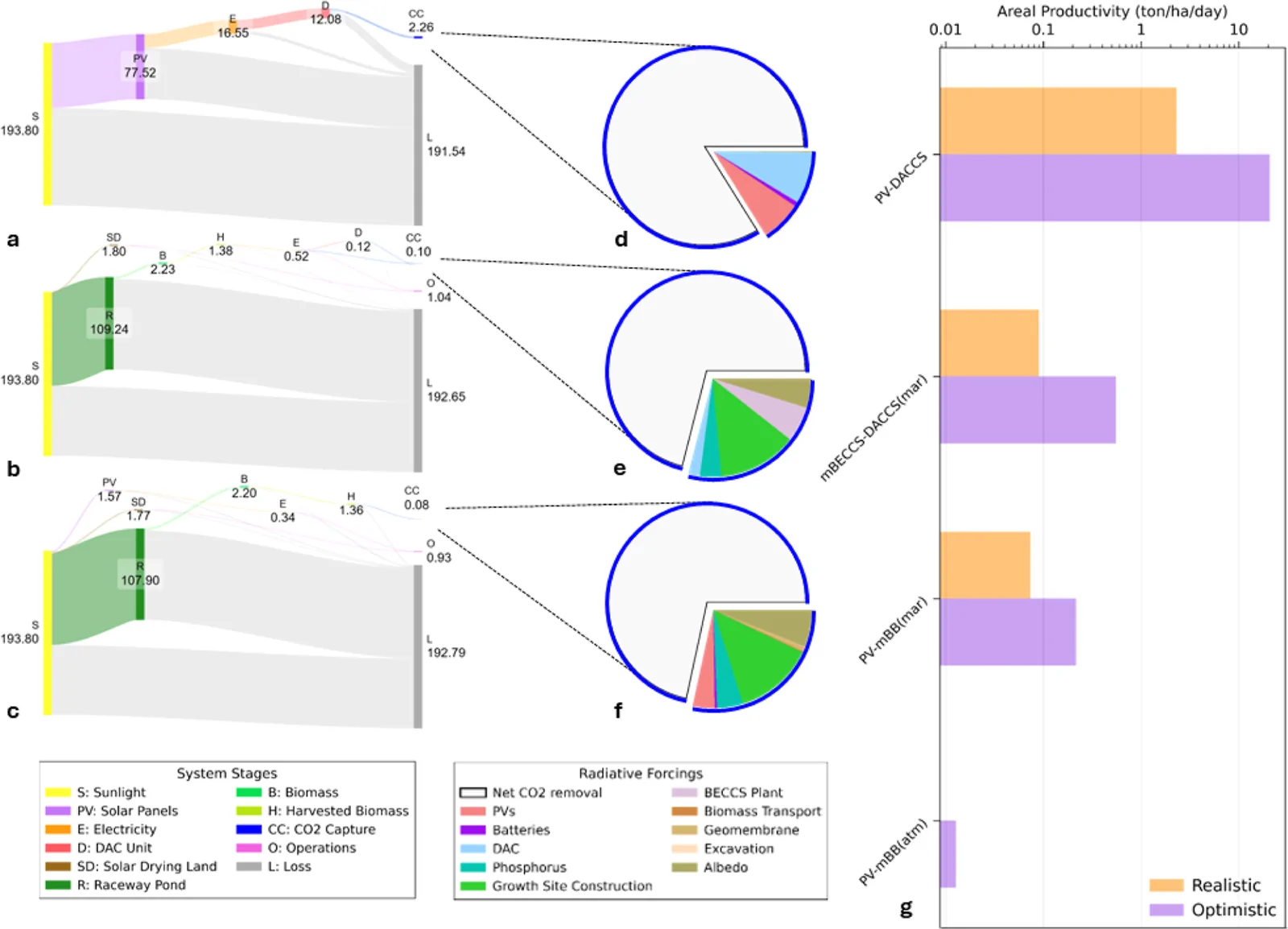
(a–c) Realistic scenario flows of solar energy (W/m^2^) through PV-DACCS, mBECCS-DACCS, and PV-mBB scenarios, respectively. Diagrams begin with energy allocation to land consuming steps according to their areal footprint, trace conversion of energy to alternative forms and corresponding losses, and end with energy usefully used in carbon capture based on separation of CO_2_ from air at a partial pressure of 423 ppm and compression to 15 MPa. Operations refer to energy productively used for necessary system operations beyond carbon capture. The H-CC energy flow for PV-mBB does not represent any undertaken process but instead indicates the fraction of buried biomass chemical energy thermodynamically necessary for CO_2_ separation of contained carbon; (d–f) embodied emissions of each considered configuration as a fraction of total gross CDR; and (g) net areal productivity.

### Net Removal Costs

Next, we calculated the levelized cost of carbon (LCOC) for each configuration. LCOC incorporates operating costs (including maintenance, labor, consumables, and insurance), capital costs (construction of facilities), project lifetimes, and end-of-life liquidation. In assessing locationally based parameters (insolation, pipeline distances and energy consumptions, burial site distances), generally favorable values are chosen reflecting a hypothetical site where a configuration may be constructed.

Realistic and optimistic costs of PV-DACCS, mBECCS-DACCS­(*mar*), and PV-mBB­(*mar*) are presented in Figure 4a–c, broken down by component, alongside PV-mBB­(*atm*) in 4d. In realistic PV-DACCS, costs of the DAC facility (blues, 59% of the total) are larger than those of the PV-battery arrays (oranges and reds, 39%) with a 2% contribution from CO_2_ storage (beiges). The DAC site contributes more greatly to LCOC in the optimistic scenario. In realistic mEBCCS-DACCS­(*mar*), algal growth site costs (greens, 66%) exceed those associated with the BPP (purples, 19%), while DAC costs are 14%; in the optimistic scenario, DAC costs rise substantially to 52% as surplus electricity allotted to DACCS increases. In realistic PV-mBB­(*mar*), costs are dominated by algal growth (95%) as opposed to the burial site (beiges, 5%) with large contributions from raceway construction, nutrients, and PV/battery arrays, while costs come to be dominated by green ammonia (see Section S20) and construction of raceway and settling ponds in the optimistic scenario. Construction costs dominate the already expensive optimistic PV-mBB­(*atm*). PV-DACCS has lower costs ($491/ton) than mBECCS-DACCS­(*mar*) or PV-mBB­(*mar*) ($642/ton and $661/ton, respectively) in the realistic scenarios, while costs of these three configurations drop and converge in the optimistic scenario—$187/ton, $176/ton, and $181/ton for PV-DACCS, mBECCS-DACCS­(*mar*), and PV-mBB­(*mar*), respectively. The equivalent microalgal production cost (from growth through drying) for PV-mBB­(*mar*) was determined to be $860/ton AFDW, at the upper end of commercial cultivation estimates (Figure S8b). This can be attributed to the water pumping and biomass settling demands in CO_2_ supply even despite favorable assumptions of operation at scale, and absence of quality and composition restrictions on microalgal biomass for CDR. DACCS costs generally agree with technoeconomic assessments in the literature after considering the extent of systems evaluated (Figure S8a). Notably, the potential for improvement shown by the cost reduction between realistic and optimistic results were higher for microalgal configurations than PV-DACCS where higher technological readiness means that the improvement space has already been partially traversed.

**Figure 4:**
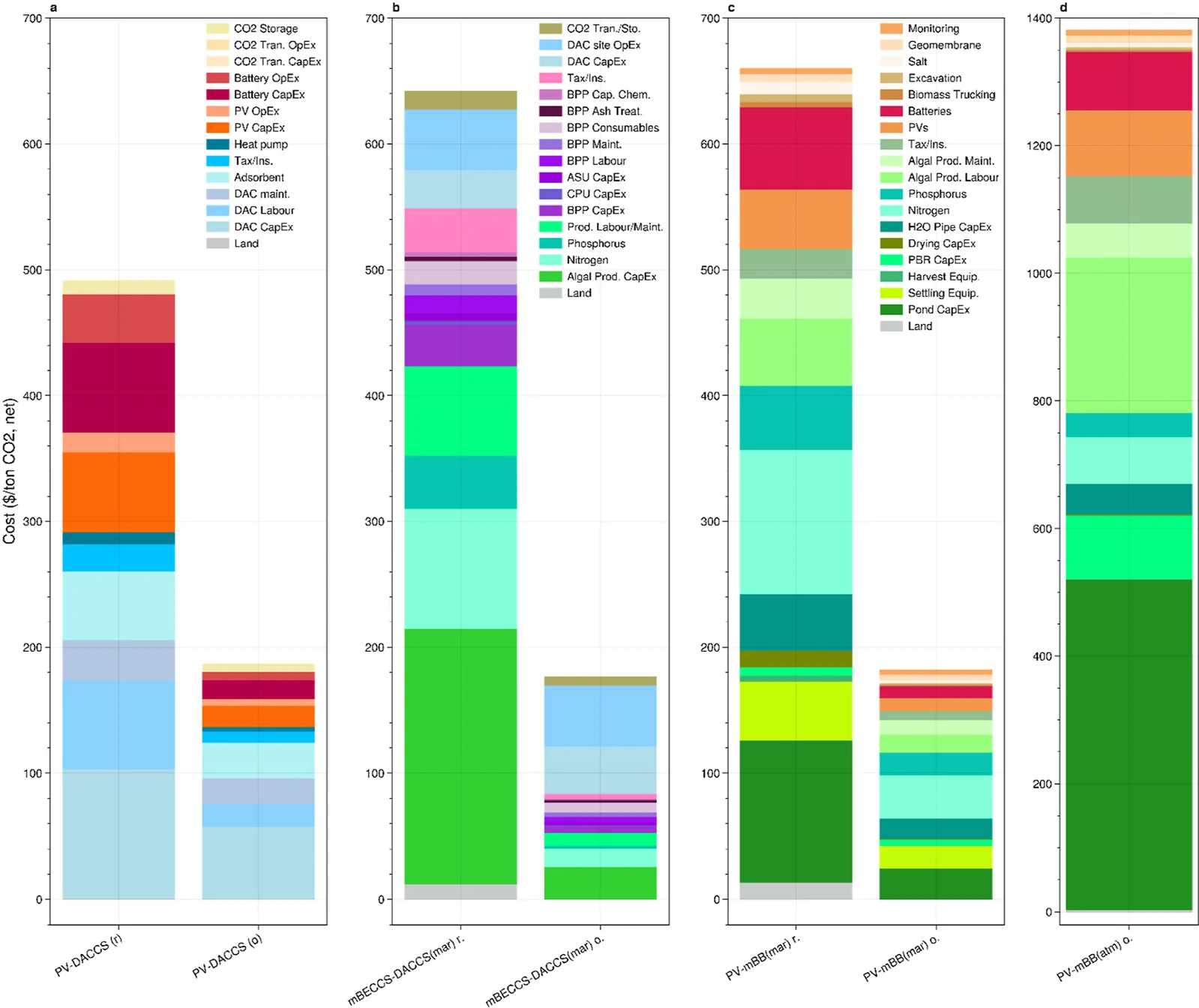
LCOC breakdowns by configuration for realistic (r) and optimistic (o) scenarios, with all removals converted into $/ton CO_2_(net removal) for (a) PV-DACCS, (b) mBECCS-DACCS­(*mar*), (c) PV-mBB­(*mar*), and (d) PV-mBB­(*atm*) (note doubled y-scale; realistic scenario not included as not net negative). In (b), algal production CapEx covers raceway, settling, harvest, drying, and pipeline as itemized in (c); similarly, DAC CapEx refers to a DAC plant and heat pump, while DAC OpEx covers heat pump OpEx and DAC labor, maintenance, adsorbent, and tax/insurance, as itemized in (a).

We performed global uncertainty and sensitivity analyses for *marine* configurations in the realistic model to indicate variation in cost outcomes and the parameters responsible for this variation, respectively. Important model parameters (OpExes, CapExes, efficiencies, and carbon intensities) were selected, and reasonable ranges for each identified parameter were chosen. Figure 5a presents results of the uncertainty analysis. In all configurations, median costs are higher than nominal values due to nonmonotonic relationships of parameters with cost. The highest probability of lower costs as well as the narrowest probability distribution are found for PV-DACCS followed by mBECCS-DACCS­(*mar*) and finally PV-mBB­(*mar*). mBECCS-DACCS­(*mar*) and PV-mBB­(*mar*) had divergent costs (non-net negative or >$5000/ton) in 0.3% and 1% of the parameter space, respectively, corresponding to high energy consumption (*W*_r_ and *h*_p_), low productivity (*y* and op), and high embodied forcings. Sensitivity results (Figure 5b) indicate that in all cases the bulk of sensitivity derives from a few efficiency parameters: algal growth rate and days of pond operation for PV-mBB­(*mar*) and mBECCS-DACCS­(*mar*) and total capture electricity requirement for PV-DACCS. Parameters $l_{set}$ and *b*_r_ were not included in global sensitivity or uncertainty as a small variation in these parameters could cause the rate of biomass loss from settling ponds during DIC replenishment to exceed biomass productivity. Instead, Figure 5c and d show base model mBECCS and mBB costs as a function of $l_{set}$ and *b*_r_. Settling pond area is adapted from that of Davis et al.^65^—where a τ_set_ of 4 h achieves an $l_{set}$ of 0.1 at a *b*_r_ of 0.5 g AFDW/L—according to τ_set_ ∝ *b*_r_^2^ in line with an aggregation or flocculation-limited regime at low densities where the settling rate is determined by collision frequency (Section S30). PV-mBB­(*mar*) is viable across a wider range of the two parameters as the ability to supply electricity is not dependent on *y*, but the configuration is more susceptible to price change based on perturbation in $l_{set}$ than mBECCS-DACCS­(*mar*) within regions of the parameter space where costs are modest. In both configurations, costs decrease with both *b*_r_ and $l_{set}$ as minimizing these values maximizes harvest *y*. However, at very low *b*_r_ close to the minimum of approximately 0.05 g AFDW/l (below which settling rate cannot supply enough biomass for harvest), costs rise due to the high cost and albedo forcing for very large settling ponds; maintenance of a robust culture at such low *b*_r_ is already very unlikely.

**Figure 5:**
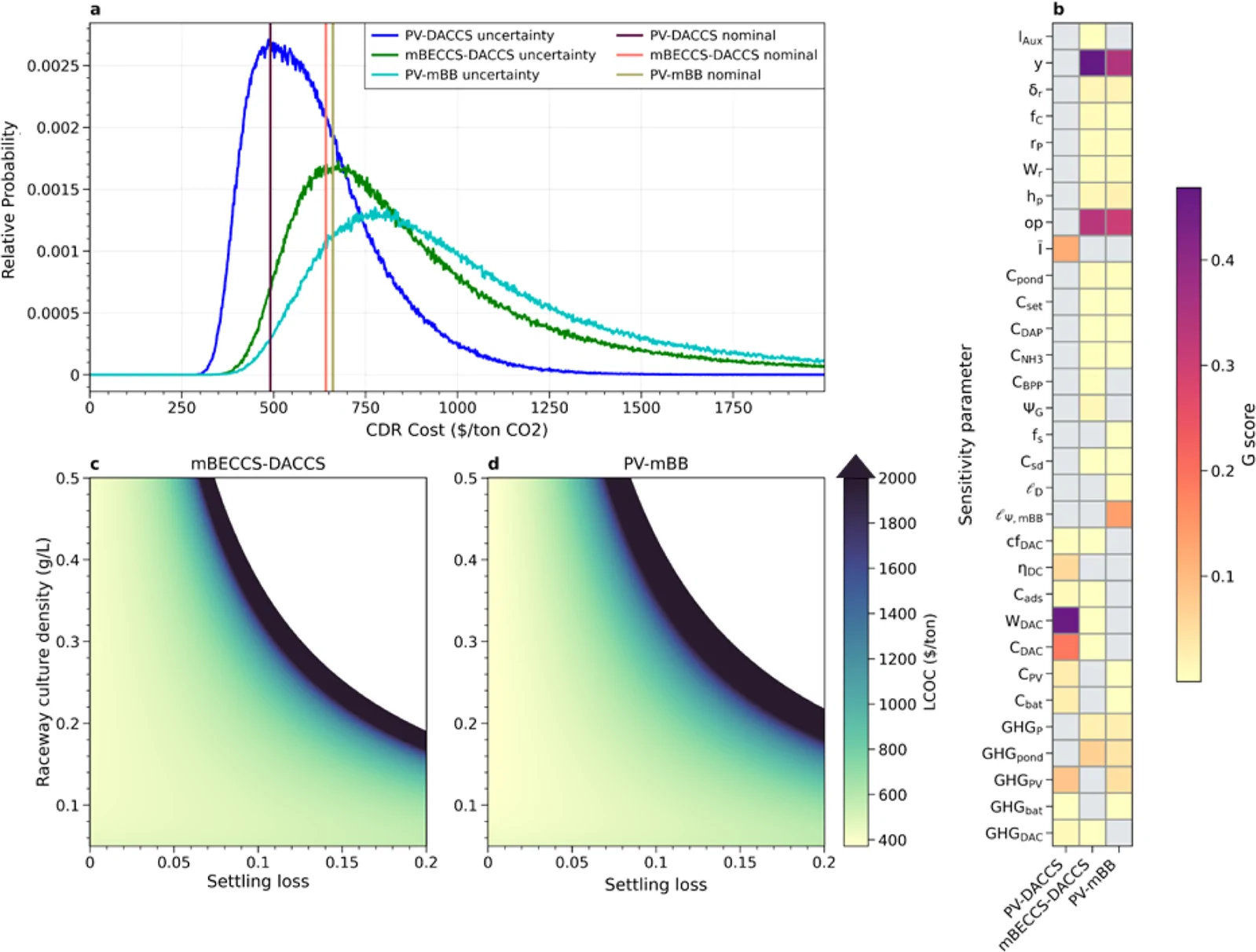
(a) Probability density function of cost in the uncertainty analysis compared with the base model for realistic scenario marine options, (b) sensitivity analysis G scores by parameter for marine options of realistic configurations (gray indicates a parameter was not included in the test space for a given configuration) and LCOC as a function of raceway culture density, and settling pond loss rate for (c) mBECCS-DACCS­(*mar*) and (d) PV-mBB­(*mar*).

## Discussion

The development of CDR strategies that can (1) reach gigaton scale annual CO_2_ removal at (2) costs approaching or below $100/ton CO_2_ without (3) the use of arable land is of great importance to meeting Paris Climate goals, stabilizing and ultimately reducing atmospheric CO_2_ concentrations, and preventing irreversible climate-induced crises. This work examined the potential of microalgal CDR strategies to meet these objectives benchmarked against the better-established DACCS technology. To minimize CDR land use, PV-DACCS proved substantially more effective. Further, we generally found that—even with the near ideal environmentally derived parameters assumed—microalgal CDR costs markedly exceeded those of PV-DACCS in the realistic scenario (regardless of configuration) and were comparable in the optimistic (for *marine* configurations). Though mBECCS with electricity sale (mBECCS-sale­(*mar*)) (considered throughout the Supporting Information) does attain costs nearly 30% below PV-DACCS in the optimistic scenario such a conclusion should be treated cautiously (see Sections S33 and S34). Between the two microalgal strategies, mBECCS produced slightly lower costs than mBB across configurations, but PV-mBB­(*mar*) has the important benefit of simplicity. While the *marine* carbon supply option requires ample energy and capital, biomass burial is a very simple and low-cost CDR strategy, especially compared with the large plants, energy consumptions, and demand for specialized capture chemicals associated with both BECCS or DAC. Biomass burial as a novel CDR strategy does, however, retain certain risks of decomposition and methane production which—while minimized through careful biomass treatment (i.e., salt addition and geomembrane encasement) and measurable^24^—may make it less preferable to geological injection used in BECCS and DACCS. Generally, we caution that more plausible operation constraints on microalgal CDR (in terms of variable electricity demand and supply) especially outside of the most optimal coastal locations which would be necessary to achieve gigaton scalability will produce costs higher than those presented herein. Meanwhile, PV-battery-DAC capacity optimization with real environmental data will not much impact PV-DACCS costs due to the higher contribution of locationally independent costs. Thus, we conclude that to serve as a reasonable CDR alternative to the more established PV-DACCS, microalgal CDR costs would need to fall substantially. This could possibly be accomplished by synergies with other industries: desalination, wastewater treatment, coproduction of high value products, or sale of nutrients given their extraction from biomass and accordingly net nutrient consumption from seawater (Section S31), though to influence technoeconomics of microalgal CDR at the gigaton scale these synergies would also need to be implemented at similarly large scales.

In addition to ramifications for CDR applications, we find that microalgal cultivation relying upon mass transfer of atmospheric CO_2_ into microalgal growth medium will generally produce costs much higher than *marine* configurations or when CO_2_ is provided through conventional sparging (Figure S8). This is primarily driven by low productivity *y*_0_, resulting from low mass transfer $J_{CO_{2}}$ even with enhancement from DIC consumption from seawater as identified in the theoretical mass-transfer model. Moreover, the analysis provided here does not consider limits to microalgal survival under extreme carbon limitation and corresponding high pH, which will almost certainly affect biomass productivity and cost (Figure S9). Further model limitations are presented in Section S35. Various strategies such as air bubbling, media circulation through an air contactor, use of biofilms to increase the mass-transfer surface area over the footprint or increase of media pH to increase chemical enhancement might all improve $J_{CO_{2}}$ but will all face serious difficulties in terms of energy cost, capital cost, or operational constraints—especially given implementation at a CDR relevant scale—as discussed in Sections S25–S29. The model for mass transfer of atmospheric CO_2_ into seawater, with calculation of chemical enhancement, can not only inform mass transfer expectations in raceways and photobioreactors but is also relevant to natural alkaline ecosystems such as soap lakes or coastal ecosystems where DIC consumption—while conserving alkalinity—by microalgae or other photoautotrophs leads to substantial pH increase.

While the *marine* CO_2_ supply option far outperforms the *atmospheric* for microalgal production and CDR in this analysis, we stress that its viability is contingent upon system parameters $l_{set}$ and *b*_r_, and to a lesser extent δ_r_ falling within quite a narrow range (Figure 5c,d). Without a highly effective biomass settling and resuspension mechanism, biomass loss during seawater circulation will approach or exceed DIC supply during the same process, with dramatic effects on viability and technoeconomics. Thus, research into microalgal sedimentation or reversible flocculation at low culture densities is of great interest not only to microalgal CDR but also to the prospects of any effort to cultivate microalgae without CO_2_ sparging. Another important limitation to *marine* configuration microalgal CDR viability relates to additionality. The model presented counts every mole of DIC consumed by harvested microalgae as CDR, which thereby assumes that all depletion of DIC in the medium will be rectified by mass transfer from the atmosphere after water is pumped back into the ocean. Though the ocean has a very large area to facilitate such equilibration, dilution means a very modest driving force and no chemical enhancement of mass transfer. This reflects a concern relevant to direct ocean capture and ocean alkalinity enhancement^71^ CDR strategies, wherein perfect equilibration is considered unlikely. Beyond the risk of downwelling an unequilibrated effluent parcel, equilibration of DIC must occur before that parcel enters the influent pipeline, or else δ_0_ will fall toward δ_r_ and viability of the settling pond DIC replenishment mechanism will again be threatened (eq 8). Carbon supply (and risks of maintaining it at scale) highlight a mechanistic similarity between the microalgal farming CDR strategies considered and established direct ocean capture CDR, which typically involves uptake and pH swing of seawater to shift DIC equilibria and capture DIC as released CO_2_ bubbles or carbonate precipitates before water neutralization and release back into the ocean.^72^ Future work may consider *marine* mBECCS or mBB as classes of microalgal direct ocean capture for better comparison of energy consumption and unified considerations of siting, seawater supply, and additionality.

## Supplementary Material



## Data Availability

Data and code supporting the findings of this work are available upon request from the corresponding author.

## Notes

Supplementary methods comprising CDR configurations (Sections S1-S3), albedo (Section S4; Fig S1), carbon and light limited growth (Section S5), water supply pipelines (Section S6), techno-economic equations (Section S7), nominal analysis parameters (Section S8; Tables S1-S3), sensitivity and uncertainty analysis (Sections S9, S10; Table S4), PV-mBB and PV-DACCS optimisation (Section S11), mass transfer modelling (Sections S12-S14; Table S5), and pH measurements (Section S15; Fig S2); Supplementary results comprising mass transfer (Sections S16, S17, S24; Figs S3, S9), burial site capacity (Section S18), comparison of alternative designs (Sections S19, S20; Fig S4), productivity, costs and comparison with literature (Sections S21-S23), and mass transfer enhancement options (Sections S25-S29); Supplementary discussion concerning biomass harvesting, nutrient supply and land use (Sections S30-S32; Fig S5-S8), mBECCS-sale and long-term mCDR perspectives (Section S33; Fig S10), contextualization of the optimistic scenario and limitations (Sections S34, S35) (PDF)

## Abbreviations

AFDW: ash free dry weight
ASU: air separation unit
BPP: biomass power plant
CapEx: capital expenditures
CDR: carbon dioxide removal
CPU: compression/purification unit
DAC: direct air capture
DACCS: direct air carbon capture and storage
DIC: dissolved inorganic carbon
mBB: microalgal biomass burial
mBECCS: microalgal biomass energy with carbon capture and storage
OpEx: operating expenditures
PBR: photobioreactor
PV: photovoltaic
TA: total alkalinity
tBECCS: terrestrial biomass energy with carbon capture and storage
VCM: voluntary carbon market

## References

[1] Core Writing Team ; Climate Change 2023: Synthesis Report. Contribution of Working Groups I, II and III to the Sixth Assessment Report of the Intergovernmental Panel on Climate Change, 184, 2023. https://www.ipcc.ch/report/sixth-assessment-report-cycle/ (accessed May 01, 2025).

[2] An advance market commitment to accelerate carbon removal, Frontier. https://frontierclimate.com (accessed May 01, 2025).

[3] Brunner C., Hausfather Z., Knutti R. (2024). Durability of carbon dioxide removal
is critical for Paris climate goals. Commun.
Earth Environ..

[4] Dooley K., Pelz S., Norton A. (2024). Understanding
land-based carbon dioxide
removal in the context of the Rio Conventions. One Earth.

[5] Dooley K., Christiansen K. L., Lund J. F., Carton W., Self A. (2024). Over-reliance
on land for carbon dioxide removal in net-zero climate pledges. Nat. Commun..

[6] Perkins O. (2023). Toward quantification
of the feasible potential of land-based carbon
dioxide removal. One Earth.

[7] Smith, S. M. ; State of Carbon Dioxide Removal: A Global, Independent Assessment of Carbon Dioxide Removal, 2024. https://static1.squarespace.com/static/633458017a1ae214f3772c76/t/665ed1e2b9d34b2bf8e17c63/1717490167773/The-State-of-Carbon-Dioxide-Removal-2Edition.pdf (accessed May 22, 2025).

[8] Babin A., Vaneeckhaute C., Iliuta M. C. (2021). Potential and challenges of bioenergy
with carbon capture and storage as a carbon-negative energy source:
A review. Biomass Bioenergy.

[9] Rosa L., Sanchez D. L., Mazzotti M. (2021). Assessment of carbon dioxide removal
potential via BECCS in a carbon-neutral Europe. Energy Environ. Sci..

[10] CDR.fyi. https://www.cdr.fyi/(accessed May 01, 2025).

[11] Fasihi M., Efimova O., Breyer C. (2019). Techno-economic
assessment of CO2
direct air capture plants. J. Clean. Prod..

[12] McQueen N. (2020). Cost Analysis of Direct
Air Capture and Sequestration Coupled to
Low-Carbon Thermal Energy in the United States. Environ. Sci. Technol..

[13] Keith D. W., Holmes G., St Angelo D., Heidel K. (2018). A Process for Capturing
CO2 from the Atmosphere. Joule.

[14] Lilonfe S., Rodgers S., Abdul-Manan A. F. N., Dimitriou I., McKechnie J. (2025). Technical, economic and lifecycle
greenhouse gas emissions
analyses of solid sorbent direct air capture technologies. Carbon Capture Sci. Technol..

[15] Bhave A. (2017). Screening and techno-economic
assessment of biomass-based power generation
with CCS technologies to meet 2050 CO2 targets. Appl. Energy.

[16] Emenike O. (2020). Initial techno-economic
screening of BECCS technologies in power
generation for a range of biomass feedstock. Sustain. Energy Technol. Assess..

[17] Ricardo Energy & Environment Analysing the Potential of Bioenergy with Carbon Capture in the UK to 2050, 2020.

[18] Buchheit, K. L. ; Lewis, E. ; Mahbubani, K. ; Carlson, D. R. Technoeconomic and Life Cycle Analysis for Bio-Energy with Carbon Capture and Storage (BECCS) Baseline, 2021. https://www.osti.gov/biblio/1810056 (accessed April 25, 2025).

[19] Puro Earth . Terrestrial Storage of Biomass, 2023. https://7518557.fs1.hubspotusercontent-na1.net/hubfs/7518557/Supplier%20Documents/Terrestrial%20Storage%20of%20Biomass.pdf (accessed May 12, 2026).

[20] Stevenson A. (2015). Is there
a common water-activity limit for the three domains of life?. ISME J..

[21] Sallon S. (2008). Germination, Genetics,
and Growth of an Ancient Date Seed. Science.

[22] Zeng N. (2024). 3775-year-old wood burial supports “wood vaulting”
as a durable carbon removal method. Science.

[23] Yablonovitch E., Deckman H. W. (2023). Scalable, economical, and stable
sequestration of agricultural
fixed carbon. Proc. Natl. Acad. Sci. U.S.A..

[24] Gooding J. L. (2023). Geologic
perspective for carbon sequestration by woody biomass burial. Sci. Technol. Energy Transit..

[25] Mühlbauer A. (2025). Assessment of technologies
and economics for carbon dioxide removal
from a portfolio perspective. Int. J. Greenh.
Gas Control.

[26] Lehtveer M., Emanuelsson A. (2021). BECCS and
DACCS as Negative Emission Providers in an
Intermittent Electricity System: Why Levelized Cost of Carbon May
Be a Misleading Measure for Policy Decisions. Front. Clim..

[27] Desport L. (2024). Deploying direct air
capture at scale: How close to reality?. Energy
Econ..

[28] Abegg M., Clulow Z., Nava L., Reiner D. M. (2024). Expert insights
into future trajectories: assessing cost reductions and scalability
of carbon dioxide removal technologies. Front.
Clim..

[29] Rueda O., Mogollón J. M., Tukker A., Scherer L. (2021). Negative-emissions
technology portfolios to meet the 1.5 °C target. Glob. Environ. Change.

[30] Fuss S., Reuter W. H., Szolgayová J., Obersteiner M. (2013). Optimal mitigation
strategies with negative emission technologies and carbon sinks under
uncertainty. Clim. Change.

[31] Abraham E. J., Linke P., Al-Mohannadi D. M. (2022). Optimization
of low-cost negative
emissions strategies through multi-resource integration. J. Clean. Prod..

[32] Babacan O. (2020). Assessing the feasibility
of carbon dioxide mitigation options in
terms of energy usage. Nat. Energy.

[33] Melis A. (2009). Solar energy
conversion efficiencies in photosynthesis: Minimizing the chlorophyll
antennae to maximize efficiency. Plant Sci..

[34] Zhu X.-G., Long S. P., Ort D. R. (2008). What is
the maximum efficiency with
which photosynthesis can convert solar energy into biomass?. Curr. Opin. Biotechnol..

[35] Zhang C. (2018). Evolving paradigms in
biological carbon cycling in the ocean. Natl.
Sci. Rev..

[36] Yang X., Liu L., Yin Z., Wang X., Wang S., Ye Z. (2020). Quantifying
photosynthetic performance of phytoplankton based on photosynthesis–irradiance
response models. Environ. Sci. Eur..

[37] Qin S., Wang K., Gao F., Ge B., Cui H., Li W. (2023). Biotechnologies for bulk production of microalgal biomass: from mass
cultivation to dried biomass acquisition. Biotechnol.
Biofuels Bioprod..

[38] Song X., Liu B.-F., Kong F., Ren N.-Q., Ren H.-Y. (2022). Overview
on stress-induced strategies for enhanced microalgae lipid production:
Application, mechanisms and challenges. Resour.,
Conserv. Recycl..

[39] Mulgund A. (2022). Increasing
lipid accumulation in microalgae through environmental manipulation,
metabolic and genetic engineering: a review in the energy NEXUS framework. Energy Nexus.

[40] Chu W.-L. (2017). Strategies
to enhance production of microalgal biomass and lipids for biofuel
feedstock. Eur. J. Phycol..

[41] Delavari
Amrei H., Ranjbar R., Rastegar S., Nasernejad B., Nejadebrahim A. (2015). Using fluorescent material for enhancing microalgae
growth rate in photobioreactors. J. Appl. Phycol..

[42] Narala R. R., Garg S., Sharma K. K., Thomas-Hall S. R., Deme M., Li Y., Schenk P. M. (2016). Comparison
of Microalgae
Cultivation in Photobioreactor, Open Raceway Pond, and a Two-Stage
Hybrid System. Front. Energy Res..

[43] Steinrücken P., Erga S. R., Mjøs S. A., Kleivdal H., Prestegard S. K. (2017). Bioprospecting
North Atlantic microalgae with fast growth and high polyunsaturated
fatty acid (PUFA) content for microalgae-based technologies. Algal Res..

[44] Long B., Fischer B., Zeng Y., Amerigian Z., Li Q., Bryant H., Li M., Dai S. Y., Yuan J. S. (2022). Machine
learning-informed and synthetic biology-enabled semi-continuous algal
cultivation to unleash renewable fuel productivity. Nat. Commun..

[45] Kamaroddin M. F., Rahaman A., Gilmour D. J., Zimmerman W. B. (2020). Optimization
and cost estimation of microalgal lipid extraction using ozone-rich
microbubbles for biodiesel production. Biocatal.
Agric. Biotechnol..

[46] Zhu Z., Jiang J., Fa Y. (2020). Overcoming
the Biological Contamination
in Microalgae and Cyanobacteria Mass Cultivations for Photosynthetic
Biofuel Production. Molecules.

[47] Naseema
Rasheed R., Pourbakhtiar A., Mehdizadeh Allaf M., Baharlooeian M., Rafiei N., Alishah Aratboni H., Morones-Ramirez J. R., Winck F. V. (2023). Microalgal co-cultivation -recent
methods, trends in omic-studies, applications, and future challenges. Front. Bioeng. Biotechnol..

[48] Schubert M. G., Tang T.-C., Goodchild-Michelman I.
M., Ryon K. A., Henriksen J. R., Chavkin T., Wu Y., Miettinen T. P., Van Wychen S., Dahlin L. R. (2024). Cyanobacteria newly
isolated from marine volcanic seeps display rapid sinking and robust,
high-density growth. Appl. Environ. Microbiol..

[49] Yu J., Liberton M., Cliften P. F., Head R. D., Jacobs J. M., Smith R. D., Koppenaal D. W., Brand J. J., Pakrasi H. B. (2015). Synechococcus
elongatus UTEX 2973, a fast growing cyanobacterial chassis for biosynthesis
using light and CO2. Sci. Rep..

[50] Włodarczyk A., Selão T. T., Norling B., Nixon P. J. (2020). Newly discovered
Synechococcus sp. PCC 11901 is a robust cyanobacterial strain for
high biomass production. Commun. Biol..

[51] U.S. Department of Energy: Office of Energy Efficiency and Renewable Energy . Multi-Year Program Plan, 2014. https://www.energy.gov/sites/prod/files/2014/07/f17/mypp_july_2014.pdf (accessed April 28, 2025).

[52] Kim D. S., Moreno-Cabezuelo J. A. ´., Schulz E. N., Lea-Smith D. J., Sagaram U. S. (2024). Recent advances
in engineering fast-growing cyanobacterial
species for enhanced CO2 fixation. Front. Clim..

[53] Even C., Hadroug D., Boumlaik Y., Simon G. (2022). Microalgae-based
Bioenergy
with Carbon Capture and Storage quantified as a Negative Emissions
Technology. Energy Nexus.

[54] Jovine, R. ; Methodology: Carbon Dioxide Removal Through Algal Biomass Burial, 2023. https://www.ecoengineers.us/wp-content/uploads/2023/11/EcoEngineers-BrilliantPlanet-Methodology.pdf (accessed April 25, 2025).

[55] Bradley, T. ; Forbes, J. ; Ingram-Hardwick, G. The potential of microalgae for large-scale carbon capture and storage: A life cycle assessment based on real-world data. Preprint, 2023. 10.21203/rs.3.rs-3147220/v1

[56] Victoria A. J., Astbury M. J., McCormick A. J. (2024). Engineering
highly productive cyanobacteria
towards carbon negative emissions technologies. Curr. Opin. Biotechnol..

[57] Vadlamani A., Pendyala B., Viamajala S., Varanasi S. (2019). High Productivity Cultivation
of Microalgae without Concentrated CO2 Input. ACS Sustain. Chem. Eng..

[58] Gao S. (2023). A newly
isolated alkaliphilic cyanobacterium for biomass production
with direct air CO2 capture. J. CO2 Util..

[59] Crowe, B. Mass Transfer Coefficients, kL, and Air-CO2 Ingassing Rates in 3.4 M2 and 1-Acre Raceway Ponds, 2022. https://www.osti.gov/biblio/2440137 (accessed May 05, 2025).

[60] Thomas P.
K., Gerlach R., Narwani A. (2026). Hunting for Extremophiles: A Systematic
Screening of Freshwater Microalgae for Tolerance to High-pH and High-Alkalinity
Cultivation. ACS Sustain. Chem. Eng..

[61] Crowe, B. ; Greer, M. ; Benemann, J. ; Gao, S. ; Edmundson, S. ; Huesemann, M. ; Nalley, J. ; White, R. Air Carbon for Algae Production (AirCAP) – Expanding Algae Resource Potential via Direct (in-Pond) Air-CO2 Capture, 2024. https://www.osti.gov/biblio/2440050 (accessed May 18, 2026).

[62] Al-Qayim K., Nimmo W., Pourkashanian M. (2015). Comparative
techno-economic assessment
of biomass and coal with CCS technologies in a pulverized combustion
power plant in the United Kingdom. Int. J. Greenh.
Gas Control.

[63] Faber G., Ruttinger A., Strunge T., Langhorst T., Zimmermann A., van der Hulst M., Bensebaa F., Moni S., Tao L. (2022). Adapting Technology
Learning Curves for Prospective Techno-Economic
and Life Cycle Assessments of Emerging Carbon Capture and Utilization
Pathways. Front. Clim..

[64] Thomassen G., Van Passel S., Dewulf J. (2020). A review on learning effects in prospective
technology assessment. Renew. Sustain. Energy
Rev..

[65] Davis, R. ; Markham, J. ; Kinchin, C. ; Grundl, N. ; Tan, E. C. ; Humbird, D. Process Design and Economics for the Production of Algal Biomass: Algal Biomass Production in Open Pond Systems and Processing Through Dewatering for Downstream Conversion, 2016. https://www.osti.gov/biblio/1239893 (accessed May 02, 2025).

[66] Kiparissides A., Kucherenko S. S., Mantalaris A., Pistikopoulos E. N. (2009). Global
Sensitivity Analysis Challenges in Biological Systems Modeling. Ind. Eng. Chem. Res..

[67] Hogendoorn J. A., Vas Bhat R. D., Kuipers J. A. M., van Swaaij W. P. M., Versteeg G. F. (1997). Approximation for
the enhancement factor applicable
to reversible reactions of finite rate in chemically loaded solutions. Chem. Eng. Sci..

[68] DeCoursey W. J., Thring R. W. (1989). Effects of unequal diffusivities on enhancement factors
for reversible and irreversible reaction. Chem.
Eng. Sci..

[69] Olander D. R. (1960). Simultaneous
mass transfer and equilibrium chemical reaction. AIChE J..

[70] Hatta, S. 613–632, 1932. https://babel.hathitrust.org/cgi/pt?id=uiug.30112032946532 (accessed May 05, 2026).

[71] Zhou M. (2025). Mapping the global variation
in the efficiency of ocean alkalinity
enhancement for carbon dioxide removal. Nat.
Clim. Change.

[72] Karunarathne S. (2025). Review on CO2 removal
from ocean with an emphasis on direct ocean
capture (DOC) technologies. Sep. Purif. Technol..

