# Supplementary material for: Potential of Microalgal Biomass Production in Coastal Deserts for Carbon Dioxide Removal
Source: Environ Sci Technol. 2026 Aug 12;60(33):23231–44. doi: 10.1021/acs.est.6c08729 (PMC13523709; doi:10.1021/acs.est.6c08729)
Supplement: Supplementary file 1 [file es6c08729_si_001.pdf]

Potential of Microalgal Biomass Production in Coastal Deserts for Carbon Dioxide Removal

Supplemental Information

William Leonard,[1*] Dale D. McClure,[2] Kok Siew Ng,[2] and Aidong Yang[1]**

[1] Department of Engineering Science, University of Oxford, OX1 3PJ, UK

[2] Department of Chemical Engineering, Brunel University of London, UB8 3PH, UK

*Email: william.leonard@eng.ox.ac.uk

**Corresponding author. Email: aidong.yang@eng.ox.ac.uk

Contents:

Supplementary methods

1. PV-DACCS configuration description
2. mBECCS-mBB configuration description
3. mBECCS-sale configuration description
4. Albedo
    a. Figure S1
5. Carbon and light limited microalgal cultures
6. Microalgal water supply pipelines
7. Technoeconomic equations
8. Nominal analysis parameter values and constants
    a. Table S1
    b. Table S2
    c. Table S3
9. Sensitivity and uncertainty analysis
10. Sensitivity analysis parameters
    a. Table S4
11. PV-mBB and PV-DACCS configuration optimization
12. Enhancement factor derivation
13. Raceway $k_L a$ calculation
14. Bubble column $k_L a$ calculation
    a. Table S5
15. Measured pH and chemical equilibria
    a. Figure S2

Supplementary results

16. Bubble column driving force consideration
17. Mass transfer prediction in raceways
    a. Figure S3
18. Burial site capacity
19. mBECCS solar and thermal drying comparison
20. mBB/mBECCS green and grey ammonia comparison
    a. Figure S4
21. Extended configuration and scenario areal productivities
    a. Figure S5
    b. Figure S6
22. Extended configuration CDR costs
    a. Figure S7
23. Microalgal growth, DAC, and total CDR cost comparison with literature
    a. Figure S8
24. PV-mBB(*atm*) and mass transfer
    a. Figure S9
25. Turbulence and J_r
26. Air sparging and J_r
27. Air contactor and J_r
28. Biofilms and J_r
29. Alkalinity enhancement and J_r

Supplementary discussion

30. Settling ponds, auto-sedimentation and flocculation
31. Nutrient supply, recovery, and depletion
32. Broad scope land use considerations
33. mBECCS-sale(*mar*) and longterm microalgal CDR prospects
    a. Figure S10
34. Contextualization of the optimistic scenario
35. Model limitation

Supplementary methods:

Section S1: PV-DACCS configuration description

The PV-DACCS system presented here consists of four components: PV array, battery storage, DAC plant, and CO_2 transport and storage. The PV array is placed on the same marginal or desert land as microalgal ponds, and generates electricity, some of which is stored by grid-scale Li-ion batteries. This PV-battery system then supplies electricity to a DAC plant. The entire system is off grid. Given the variable nature of the renewable energy supply (even with battery storage), both curtailment and a reduced DAC plant capacity factor are assumed.

The DAC plant examined in the realistic scenario is based on the model of Climeworks, a DAC industry leader. The Climeworks DAC process involves cyclic temperature-pressure swing adsorption with amine based sorbents built into relatively small and modular plants.[1] Physical sorbents have the advantage of requiring cycle temperature swings of less than 100 °C which makes such low temperature DAC suitable for use of heat pumps, and allows for relatively good efficiency relying on a fully electric energy supply.

Meanwhile, in the optimistic scenario, DAC performance is less explicitly linked to the Climeworks model, instead relying on data from novel DAC sorbents, that have potential for substantially reduced energy requirement for desorption compared to amine-based alternatives.[2] In particular, the optimistic scenario parameters derive primarily from a proposed zeolite-based low temperature DAC model,[3] performance of which is in line with industry objectives of 10% thermodynamic efficiency for CO_2 capture (even though the specific technology in reference is designed for cold climates and would accordingly be unsuitable for the majority of locations considered in the regional and global analyses herein).

After CO_2 is captured, it is transported via pipeline to a geological storage site for injection. Though the DAC plants are assumed to be small, pipelines are assumed to have relatively large diameters and high flow rates associated with optimal pipeline performance, consistent with an array of DAC plants feeding a single pipeline. CO_2 losses from the pipeline or during injection are often assumed at rates between 1.8% and 6% of total captured CO_2[4] but are ignored herein due to lack of bounding for a comparable biomass loss rate for the mBB process.

It is important to clarify that the PV-DACCS configuration is presented in this study for comparison with microalgal CDR as a representative scalable option. This work makes no survey of the DAC configuration space, and accordingly, alternative DAC power sources such as on or offshore wind,[5] or geothermal[1] may perform better or produce lower CDR costs.

Section S2: mBECCS-mBB configuration description

mBECCS-mBB is designed as a configuration where electricity demand for mBB pond operation and biomass harvest is met through surplus electricity from allocation of a sufficient amount of biomass to mBECCS. mBECCS-mBB is otherwise designed just as PV-mBB. The fraction of total biomass required for combustion f_{mBECCS} is calculated according to equation (S1):

$$f_{mBECCS} = \frac{W_m}{W_{BPP} \cdot \left(1 - \ell_{\Psi,mBECCS-mBB}\right)} \tag{S1}$$

where W_m and W_{BPP} are pond energy consumption and net BPP electricity yield (kWh/ton AFDW), and $\ell_{\Psi,mBECCS-mBB}$ is the configuration electricity loss rate. Demand for battery storage and curtailment is removed

under the assumption used for mBECCS-DACCS that collections of biomass power plants produce a constant baseload electricity even if individual plants do have a capacity factor of less than 1 due to maintenance. CO_2 capture from BPP plants is transported via pipeline for storage under the same assumptions as in mBECCS-DACCS, but with lower throughput.

Section S3: mBECCS-sale configuration description

Instead of using excess electricity from mBECCS to operate DAC or mBB, the mBECCS-sale configuration assumes that this electricity can be sold. The off-grid constraint is accordingly relaxed in this configuration. Cost of grid connection (which may not be trivial given that sites proposed in this work are on marginal and desert lands that tend to be far from sites of electricity demand) is not directly assessed but instead internalized via a parameter f_{prof} included to indicate the fraction of total electricity surplus that leads to profit (after consideration of curtailment, grid connection costs, etc.) based on the assumed electricity price. The total revenue of electricity sale rev_E (\$/ton biomass, considered in LCOC calculation as a negative OpEx) is calculated according to equation (S2):

$$rev_E = W_{BPP} \cdot f_{prof} \cdot C_E \tag{S2}$$

where C_E is electricity purchase or sale price in \$/kWh. Many dynamics of grid connection and operation of mBECCS—as would be standard for tBECCS for power supply—are ignored by this simple equation. We offer the mBECCS-sale configuration for purposes of comparison with both mBB and tBECCS but posit that it is not currently the configuration most well-equipped for CDR in the context of the framing principles with which this paper engages (see section S31).

Section S4: Albedo

Usage change from desert to PVs, water (raceway and settling ponds), or biomass (drying land) substantially decreases the albedo (fraction of incoming shortwave radiation reflected by the surface) of a parcel of land. This is accompanied by a positive radiative forcing. Albedo consideration is occasionally given for afforestation and tBECCS projects and the magnitude of forcing can at times offset or counteract the forcing caused by carbon removal.[6] Accordingly, quantifying the magnitude of this effect is very important for indication of the viability of microalgal CDR to actually mitigate climate change. The global radiative forcing due to albedo change of PV-mBB (and analogously for other configurations) ΔRF_E (W/m^2), was calculated according to equation (S3):

$$\Delta \mathrm{RF_E} = \frac{\bar{\mathrm{I}} \cdot \sum_{\mathrm{i}}(\Delta\alpha_{\mathrm{i}} \cdot \mathrm{A_i})}{\mathrm{G_{PV-mBB}} \cdot \mathrm{A_E}} \tag{S3}$$

where $\Delta\alpha$ is the unitless change in albedo over the footprint of a specific land requiring process i for PV-mBB (as in equation (14)), A_i is that process' land requirement (ha/ha), and A_E is the earth's surface area (ha). This yields a global radiative forcing of 0.093 and 0.049 W/m^2 for each gigaton per year of net removal capacity in the realistic and optimistic scenarios respectively.

How exactly to compare this albedo effect with—or integrate it into—CDR modelling is not trivial. At base level this is because radiative forcing due to albedo behaves differently than a CO_2 pulse emission due to the finite lifetime of CO_2 in the atmosphere, the continuous CO_2 removals on a single parcel of land (under the considered

CDR strategies), and the potential for albedo reversion to original state after project completion, not to mention secondary albedo effects such as increased cloud formation driven by evaporation from microalgal ponds. Here we use the $\sum TDEE$ approach following Bright et al.,[7,8] whereby the effective of a nonconstant albedo change is compared to CDR by determining the annual negative and/or positive CO_2 emissions pulses that cause equivalent forcing. Time-dependent emissions equivalence $TDEE$ (ton CO_2/ha) was calculated according to equation (S4):

$$\mathrm{TDEE} = \mathrm{A}_{\mathrm{E}}^{-1} \cdot \mathrm{k}_{\mathrm{CO}_2}^{-1} \cdot \Gamma_{\mathrm{CO}_2} \cdot \mathrm{RF}_{\Delta\alpha}^{*} \tag{S4}$$

where k_{CO_2} is the global mean radiative efficiency of CO_2 (W/m^2/ton), Γ_{CO_2} is a (unitless) lower triangular matrix with residual atmospheric fraction of a CO_2 pulse emitted in a given year (in other words, a more precise representation of CO_2 lifetime, as referred to by figure S1 below), and $RF_{\Delta\alpha}^{*}$ is a column vector of the annual radiative forcings resulting from the albedo change (W/m^2). Γ_{CO_2} is here taken equivalently for each year from the impulse response function of a CO_2 pulse at 389 ppm.[9] $RF_{\Delta\alpha}^{*}$ was calculated according to equation (S5):

$$RF_{\Delta\alpha}^{*}(t) = \bar{I} \cdot (\alpha(t) - \alpha_0) \tag{S5}$$

where $\alpha_0 = 0.3$ is the (unitless) albedo of desert land and $\alpha(t)$ is the albedo of the parcel of land at a year t equal to $\alpha_r = 0.1$ for raceway ponds, settling ponds, and drying land in mBB and mBECCS configurations and $\alpha_{PV} = 0$ for PV in mBB and DACCS. Beyond the lifetime of the projects, $RF_{\Delta\alpha}^{*}$ becomes zero, consistent with desert

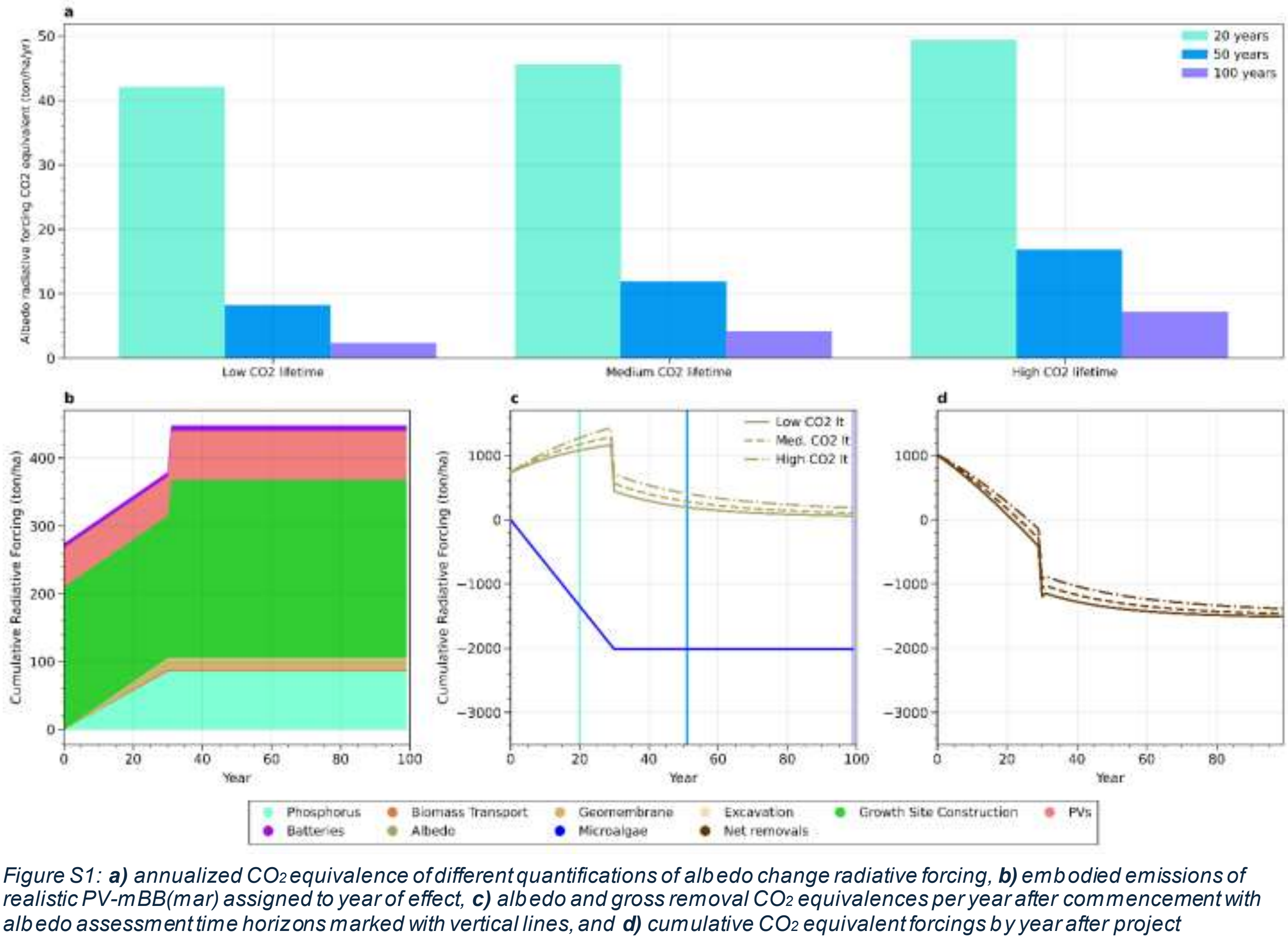

Figure S1: ***a)*** *annualized CO_2 equivalence of different quantifications of albedo change radiative forcing,* ***b)*** *embodied emissions of realistic PV-mBB(mar) assigned to year of effect,* ***c)*** *albedo and gross removal CO_2 equivalences per year after commencement with albedo assessment time horizons marked with vertical lines, and* ***d)*** *cumulative CO_2 equivalent forcings by year after project commencement.*

restoration (and pond draining) following project completion. The dimension of the Γ_{CO_2} matrix, corresponding to the time horizon over which albedo effect is converted to CO_2 equivalence determines the time point at which albedo effect will be assessed and need not be limited by the length of the nonzero change of albedo effect. Here we use a time horizon of 100 years. Finally, the albedo CO_2 equivalence is embedded into the nominal model by summation of the annual CO_2 equivalent pulses as per equation (S6):

$$\mathrm{GHG}_{\alpha} = \sum_{0}^{100} \mathrm{TDEE} \tag{S6}$$

Recall that GHG_α is then annualized over CDR project lifetime τ while its effect on net removals R is assessed (equation (1)). Notably, the magnitude of GHG_α is highly dependent on embedded assumptions such as values for k_{CO_2} (which is variable and dependent on atmospheric CO_2 concentration), choice of project lifetime, time horizon in equation (S4), and whether land is actually converted back to its native albedo. Figure S1a shows the value of GHG_α for realistic mBB (the albedo effect for mBECCS only differs slightly according to lifetime and the small PV footprint, the DACCS albedo effect can be neglected due to high areal productivity). Figure S1b shows the cumulative forcings from the construction and operational resource consumption of the PV-mBB system, given different assumptions in TDEE calculation (PV, battery and growth site construction are treated as pulse emissions 80% in year 0 and 20% in year $N+1$, to approximate the split between manufacturing and end-of-life embodied emissions). Complementary to S1b, S1c shows cumulative forcings of microalgae (gross removals) and albedo, while S1d finally shows the net cumulative forcing. Note that net radiative forcing of PV-mBB is positive for the majority of project lifetime before the effect of removals outweighs albedo and construction effects, and that land restoration following project completion (especially given short project lifetimes) has a large effect on long term emissions equivalences.

Section S5: Carbon and light limited microalgal cultures

CO_2 sparged microalgal cultures are generally light limited or demonstrate co-limitation between light (or temperature) and carbon, which thereby leads to behavior such as annual variation in biomass productivity according to colder temperatures and reduced insolation in the winter.[10,11] In this analysis, however, we assume that cultures are uniquely limited by carbon and thereby the supply pathways outlined in the *atmospheric* and *marine* options. While reduced temperatures and light intensities in winter may be expected to reduce the ability of microalgal cultures to survive at very low DIC concentrations which will thereby affect the rate of gas-liquid mass transfer in both options and the efficiency of the DIC replenishment scheme in the *marine* option, we do not assume that temperature and light intensity representative of suitable microalgal CDR locations will be insufficient to maintain growth, even in the winter. mBB in particular is contingent on desert conditions to prevent water intrusion into buried biomass. In a representative coastal desert, such as Marsa Matruh in Egypt, 99% of days have a photosynthetically active radiation[12] above 168 μmol/m^2/s even after reducing insolation to account for reflection off of the raceway pond surface.[13,14] This insolation is sufficient for the realistic and optimistic *marine* option biomass productivities of 18 and 25 g biomass/m^2 with a photosynthetic efficiency of 3.7% and 5.2%. While achieving such photosynthetic efficiencies in raceway cultures, especially at the high midday light intensities entailed, is not trivial, we assert that this is a strain selection issue, and that such strains do exist—as demonstrated by winter productivities of 14 g/m2/day[15] in carbon replete environments and theoretical photosynthetic efficiency maxima extending above 10%.[16] Accordingly, and so as to prioritize the focus on carbon supply, any effects of colimitation by light are

not considered in the technoeconomic model beyond the capacity factor op which allows for days with no assumed biomass productivity (see table S1).

Section S6: Water supply pipelines

Water supply pipeline diameter in the *atmospheric* option was based on the water influx Q_{in} necessary to maintain constant water and salinity (the latter being higher than that of the ocean) in raceways which are losing water to evaporation. While Q_{in} for the *marine* option is calculated based on DIC demand (equation (8)), for the *atmospheric* option Q_{in} is calculated according to equation (S7):

$$Q_{in} = \Phi \cdot \frac{S_r}{S_r - S_0} \quad (S7)$$

where Φ is the evaporation rate (m/year), and S_0 and S_r are the salinities (g/kg) of the ocean and raceway respectively. While influent pipeline energy requirement is calculated by equation (11), effluent pipelines are assumed to travel downwards, and so energy consumption is neglected for both carbon supply options.

Section S7: Technoeconomic equations

The annuity factor (unitless) and liquidation value ($), used for calculating levelized cost of carbon in equation (19) are calculated via equations (S8) and (S9):

$$crf = \frac{r \cdot (1 + r)^{\tau}}{(1 + r)^{\tau} - 1} \quad (S8)$$

$$LQ = \frac{c_{LQ} \cdot CapEx}{(1 + r)^{\tau}} \quad (S9)$$

where τ is lifetime as elsewhere, r is weighted average cost of capital set at 0.06 and c_{LQ} is the fractional value retained at the time of liquidation (20%).

Section S8: Technoeconomic analysis parameter values and constants

Technoeconomic analysis constants are given in tables S1-S3. Table S1 gives physical constants and process efficiencies; table S2 offers economic parameters; table S3 provides embodied emissions and albedo effects.

Table S1: Process efficiencies and physical constants

Parameter (units)	Symb.	Real. value	Realistic notes	Opt. value	Optimistic notes
Microalgal ponds					
PBR/inoculum electricity consumption (kWh/ha/day)	W_{PBR}	4.5	50% increase from opt. for maintaining high growth	3	[10]
Paddlewheel/growth elec. cons. (kWh/ha/day)	W_r	50	[10]	40	[10]
Harvest/dewatering elec. cons. (kWh/ha/day)	W_h	14	[10]	8	[17] Given bioflocculation/ autosedimentation water content

Biomass concentration raceway pond [*marine*] (g AFDW/L)	b_r	0.2	Based on optimistic specific growth rates and culture depth of 0.2-0.4 m,[18–20] see note[1]	0.2	
Sediment pond output biomass concentration (g AFDW/L)	b_{set}	10	[10]	111	[17]
Biomass concentration membrane filter (g AFDW/L)		10	[10]	n/a	
Membrane filter output biomass concentration (g AFDW/L)		130	[10]	n/a	
Centrifuge output biomass concentration (g AFDW/L)		200	[10]	n/a	
Filter press output biomass concentration (g AFDW/L)		n/a		200	[10]
Dewatering output moisture (ton H_2O/ton wet biomass)	M_0	0.8	converted from above	0.8	converted from above
Final moisture content (ton H2O/ton wet biomass)	M_f	0.08	[21] suitable for mBB and mBECCS	0.08	
Evaporation energy req. (MJ/kg H_2O)	H_{ev}	2.53	Heat of vaporization + energy to raise temp. 65 C	2.53	
Solar drier efficiency	η_{sd}	0.4	[22]	0.62	[23]
Seawater DIC concentration (mM)	δ_0	2.3	[24] upper bound	2.3	
Raceway DIC concentration (mM)	δ_r	0.3	Laboratory microalgal growth experiments	0.3	
Setling pond frac. biomass dissolved phase	ℓ_{set}	0.1	[10]	0.05	[25]
Atmospheric opt. CO2 mass transfer (g/m^2/day)	J_r	3.25	Mass trans. model, [DIC] = 0.3 mM, [total phosphate] = 0.05 mM, [ammonia] = 0.8 mM	4.9	Mass trans. model, [DIC] = [total phosphate] = [ammonia] = 0
Marine opt. CO2 mass transfer (g/m^2/day)	J_r	3.25	Realistic *atmospheric*	3.25	Realistic *atmospheric*
Atmospheric algal growth rate (g AFDW/m^2/day)	y_0	1.77	Converted from J_r	2.51	Converted from J_r
Marine algal growth rate (g AFDW/m^2/day)	y_0	18	Somewhat poorly constrained due to novelty of carbon supply scheme, based on high productivities observed in high pH growth with alternative carbon supply[26–28]	25	Largely operationally constrained by DIC supply and settling mechanism, plausible based on non-sparged growth data[11,29]
Biomass HHV (MJ/kg)	HHV	21	[30]	23	[31] higher values possible but tradeoff with y
Algal carbon content (g C/g biomass)	f_C	0.5	[30] biofuels standard	0.532	[32] average marine microalga
Operating days per year	op	330	[10]	365	[10] sensitivity maximum
C:P biomass molar ratio	r_P	86	[33,34]	134	[34]
Phosphorus recovery frac. [mBECCS-DACCS, mBECCS-sale]	η_P	0		0.9	phosphorus recovery from bottom ash at BPP
Phosphorus recovery frac. [PV-mBB]	η_P	0		0	
Phosphorus recovery frac. [mBECCS-mBB]	η_P	0		0.023	90% recovery assumed from mBECCS biomass only
C:N biomass molar ratio	r_N	7.3	[34]	7.3	[34]
Raceway pond filling ratio	fr_r	0.658	[10]	0.8	equal to PV array maximum
Evaporation rate (m/year)	Φ	1.5	[35]	1.5	
Ocean salinity (g/kg)	S_0	35	[36]	35	
Raceway salinity (g/kg)	S_r	52.5	Optimization, see section S11	52.5	Optimization, see section S11

[1] Data on minimizing culture b_r are poorly constrained due to the general incentive towards high b_r (>0.5 g AFDW/L, generally a tool to achieve higher biomass productivities and increased robustness).[10] A raceway depth of 0.2-0.4 m at 0.2 g AFDW/l implies sustained specific growth rate of 0.2-0.37/day which align with observations sited. Use of a lower b_r also lowers the buffer to resist culture crash due to small perturbations such as contamination, photo effects, or other environmental changes.

Influent water supply pipeline diameter [*atmospheric*] (m)	$d_{p,H2O}$	0.373	Calculated from water pipeline velocity of 1.84 m/s (ref[37]) and 250 ha service area per pipeline, see note[2]	0.399	
Influent water supply pipeline diameter [*marine*] (m)	$d_{p,H2O}$	1.95	Calculated as *atmospheric* realistic	2.58	
Biomass power plant (500 MW rated capacity)					
BPP gross electrical efficiency	η_{comb}	.381	[38]	0.455	[39]
ASU elec. cons. (kWh/ton O_2)	W_{O2}	200	[40] industry standard	137	[41] chemical looping target
O req. for comb. (g O_2/g biomass)	r_{O2}	1.67	[30] microalgal biomass oxidation stoichiometry	1.67	[30]
O_2 excess fraction	ℓ_{O2}	0.05	[42]	0.02	
CPU elec. cons. (kWh/ton)	W_{CPU}	164	[43] see note[3]	132	[38]
Other auxiliary power (kWh/kWh gross)	W_{aux}	0.05	[38]	0.03	[45]
Carbon capture efficiency	η_{cc}	0.94	[46]	0.97	[47]
BPP capacity factor		0.85	[48] common high end	0.9	industry target
Frac. of elec. profited (mBECCS-sale)	f_{prof}	0.233	[49] Allowing 2/3 of elec. sale revenue to offset infrastructure construction cost	0.7	[49] based on electricity demand profiles
DAC plant					
DAC elec. cons. (kWh/ton)	Ψ_{DAC}	300	[50] values for Climeworks, see note[4]	223	[3] zeolite sorbent performance
DAC heat cons. (kWh/ton)	H_{DAC}	2000	[50] Climeworks	1110	[3]
DAC compression elec. (kWh/ton)	W_{comp}	101	[1]	86.1	[44]
Heat pump COP	cop	3	[50] LT DAC standard	3.5	[51] for Climeworks 120 C desorption temperature
DAC capacity factor [PV-DACCS]	cf_{DAC}	0.81	[52] 90% (maint. only), 90% (power avail.)	0.855	[53] 95% (maint. only), 90% power availability
DAC capacity factor [mBECCS-DACCS]		0.765	Uncorrelated BPP and DAC maint.	0.9	Simultaneous maintenance
PVs and batteries					
Average Insolation (kWh/m²/day)	$\bar{I}$	4.65	[54] global average	5.93	[55] desert conditions (no clouds)
PV efficiency (kWh DC/kWh insolation)	η_{DC}	0.22	[56] good commercial performance	0.4	[57] Novel PV lab performance
Inverter efficiency (kWh AC/kWh DC)		0.97	[58]	0.98	[59]
Solar panel filling ratio	fr_{PV}	0.4	[60]	0.8	[60]
Total battery/curtailment elec. loss frac. [PV-DACCS]	ℓ_{Ψ}	0.27	Optimization, see section S11	0.25	Optimization, see section S11
Total battery/curtailment elec. loss frac. [PV-mBB]	ℓ_{Ψ}	0.35	Optimization, see section S11	0.35	Optimization, see section S11
Total curtailment elec. loss frac. [mBECCS-mBB]	ℓ_{Ψ}	0.18	Set as half of the PV-mBB loss rate, see note[5]	0.18	

[2] Practically pipeline diameter would be constrained to readily available pipe schedules which may slightly influence capital and energy expenditures. Potential optimization of pipe diameter, fluid supply and pipeline service area is ignored here.

[3] CPU outlet pressure from both Cau and Wei are only 11 MPa. 9.4 kWh/ton and 6.1 kWh/ton are added for realistic and optimistic scenarios respectively for the additional compression necessary to reach 15 MPa.[44]

[4] As described, the PV-DACCS parametrization is based on the company Climeworks. Differing primary sets of energy efficiency values exist in Fasihi et al, and Deutz and Bardow. We adopt the Ψ_{DAC} and H_{DAC} numbers used in the former[50] while the latter offers values of $\Psi_{DAC} = 700$ kWh/ton and $H_{DAC} = 3310$ kWh/ton in a today scenario with future targets of $\Psi_{DAC} = 500$ kWh/ton and $H_{DAC} = 1500$ kWh/ton with cop=2.51,[1] which in both scenarios lead to substantially higher W_{DAC} than that of Fasihi. For this analysis we use the values of Fasihi et al as they are more representative of the best values in the DAC industry for purposes of rigorous comparison with mBB and mBECCS.

[5] In theory electricity supplied by mBECCS for mBB could be nearly lossless as biomass for mBB could be stored and input to the BPP in exact correlation with demand. This, however, would be associated with a low BPP capacity factor and extra cost. Instead, the loss rate is set at half of that of PV-mBB (itself derived from the regional analysis optimization), to reflect that both electricity yield from mBECCS

Battery: PV capacity ratio (kWh per kW) [PV-DACCS]		2.73	Optimization, see section S11	2.88	Optimization, see section S11
Battery: PV capacity ratio (kWh per kW) [PV-mBB]		2.5	Optimization, see section S11	2.6	Optimization, see section S11
CO_2 transport and storage					
Pipeline distance (km)		165	[61]	132	[61] 20% reduction MENA vs. Europe
Pipeline diameter [PV-DACCS] (m)	$d_{p,CO2}$	0.889	[62] Based on flow rate, diameter limits	0.889	[62]
Pipeline diameter [mBECCS -DACCS] (m)	$d_{p,CO2}$	0.324	See note[6]	0.496	
Pipeline diameter [mBECCs-sale] (m)	$d_{p,CO2}$	0.297		0.349	
Pipeline diameter [mBECCS-mBB] (m)	$d_{p,CO2}$	0.272		0.221	
Burial site					
Distance to burial site (km)	d_T	15.8	Optimization, see section S11	7.5	Optimization, see section S11
Crow factor (km road/km Euclidean)	c_{crow}	1.6	conservative value for remote locations	1.4	[64]
Trucking return factor ($ roundtrip/$ loaded)	c_{ret}	1.75	this work	1.75	
Truck emissions return factor (ton CO_2(eq) roundtrip/loaded)		1.75	[65] calculated from data	n/a	(no footprint, see table S3)
Excavation depth (m)	h_x	3	this work	2	
Salt mass requirement (ton salt/ton AFDW)	f_s	0.667	[21]	0.25	[21]
Biomass buried layer thickness (m)	h_b	4	this work	4	
Biomass density (ton/m^3)	ρ_{bio}	0.6	[66]	0.6	[66]
Salt density (ton/m^3 salt)	ρ_s	2.16		2.16	
Decomposition frac.		0	see table S4	0	

Table S2: Configuration economic parameters					
Parameter (units)	Symbol	Real. value	Realistic notes	Opt. value	Optimistic Notes
General technoeconomic parameters					
Discounting rate	r	0.06		0.06	
PV-DACCS lifetime	$\tau_{PV-DACCS}$	25		40	
mBECCS-[DACCS, sale] lifetime	τ_{mBECCS}	25	also mBECCS-mBB real.	40	
PV-mBB lifetime	τ_{PV-mBB}	30		50	also mBECCS-mBB opt.
Land					
Land cost ($/ha)		5200	[10] reduced by half for remote, MENA context	206	[67]

(without biomass storage) and pond electricity consumption will be correlated with biomass productivity directly and via temperature and evaporation rate.

6 Whereas CO_2 pipelines for PV-DACCS are assumed to be operating at realistic limits on their scale (a diameter of 0.889 m and flow rate of 52 Mt CO_2/year), corresponding to PV areas of 180 and 40 km^2 in realistic and optimistic scenarios respectively, this same pipeline limitation for mBECCS-DACCS would imply algal pond areas of 11300 and 2300 km^2 which is too large to be serviced by a single storage pipeline. Accordingly, pipeline diameter for mBECCS-DACCS was determined based on an assumption that pipelines serve an area of algal ponds equal to 1/64th or 1/100th in realistic or optimistic scenarios of the area corresponding to a circle with radius equal to pipeline distance. Given corresponding flow rates of 3.9 and 12.3 Mt CO_2/year, pipeline diameter $d_{pipe,CO2}$ (m) was calculated according to equation (S10):

$$d_{p,CO2} = 0.132 \cdot \left(Q_{CO_2}\right)^{0.46} + 0.0762 \quad \text{(S10)}$$

with Q_{CO_2} in Mt/year, as an empirical curve fitted to optimal CO_2 pipeline diameter calculation by Heddle.[63]

Microalgal pond[7]					
Growth pond CapEx ($/ha)		7.74e4	[10] (10 ha minimum), 10% reduction for MENA /scaling	4.77e4	[10] (50 ha minimum), 20% reduction for MENA/ scaling
PBR/inoculum CapEx ($/ha)		4594	[10]	9187	[10] doubled, higher precision equipment for optimization
Sediment pond CapEx ($/(m^3/day))		9.36	[10] 15% increase over Davis	6.92	[17] faster sedimentation rate possible
Filtration CapEx ($/(m^3/day))		250	[10]	n/a	
Centrifuge CapEx ($/(m^3/day))		671	[10]	n/a	
Filter press CapEx ($/(m^3/day))		n/a		98.2	[10] given assumed culture density of 111 g/L
Solar drying CapEx ($/m^2 sd)		55.7	[68] solar greenhouse dryer (scaling factor=0.8; 50 ha growth ponds)	6.3	[10] liner cost (sun drying system)
Algal raw labor cost [*marine*] ($/ha/year)		1974	[10] (50-acre pond)	1382	[10] savings consistent with MENA vs. US labor costs
Algal raw labor cost [*atmospheric*] ($/ha/year)		1545	[10] reduced personnel because of low biomass productivity	1081	As above
Labor burden [algal pond, biomass power, DAC]	c_{LB}	0.3	[69] see total labor cost note;[8] consistent labor burden used for all modules, see note[9]	0.3	
DAP cost ($/ton DAP)	C^*_{DAP}	643	[70]	550	[71]
Grey ammonia cost ($/ton NH_3)		500	[72] conservatism for volatility	380	[73]
Green ammonia cost ($/ton NH_3)	$C^*_{NH_3}$	1153	[74] TEA	500	[75] projection
Maintenance cost [*atmospheric*] ($/ha/year)		438	[10] 3% of harvest equipment, 0.5% other	289	[10] 3% of harvest equipment, 0.5% other
Maintenance cost [*marine*] ($/ha/year)		1522	As above	1369	
Additional tax/property insurance (frac. CapEx)		0.007	[10]	0.007	[10]
Water pipeline cost ($/km)		5.23e6	[37] sf=0.9 with $d_{p,H2O}$	4.70e6	[37]
Effluent pipeline cost (frac. influent)		0.309	[63] Given weight of cost components	0.309	
Water pipeline Opex ($/km)		5286	[63]	5286	
Biomass power plant (all costs per 500 MW rated capacity)					
Biomass plant CapEx ($)		1.24e9	[76] (sf=0.85)	5.01e8	[39] (sf=0.7)
ASU CapEx ($)		1.88e8	[77] (sf=1)	2.01e8	[78] (sf=1)
CPU CapEx ($)		8.59e7	[79] (sf=0.85)	6.80e7	[80] (sf=0.7)
BPP raw labor cost ($/year)		2.44e7	[69]	1.71e7	[69] savings consistent with MENA vs. US labor costs
BPP consumables ($/year)		4.17e7	[69]	4.17e7	[69]
BPP ash disposal/ treatment ($/year)		7.79e6	[69]	9.95e6	[69] See note[10]
BPP capture chemicals ($/year)		7.84e6	[69]	5.49e6	[69] 30% savings (see DAC sorbents opt. vs. real.)
BPP maintenance ($/year)		2.01e7	[69]	2.01e7	[69]

[7] Instead of assuming scaling factors for the algal pond, costs per ha from Davis are used or adjusted (primarily in optimistic) given system improvement potential.

[8] Total labour cost for any subsystem is calculated according to equation (S11):

$$OpEx_{lbr} = (1 + c_{LB}) \cdot C_L \qquad (S11)$$

where c_{LB} is the labour burden and C_L is the raw labour rate.

[9] Here we depart from the NREL report which offers a labour burden of 0.9,[10] as 0.3 is more consistent with labour burden values across industry and yields consistency in comparison.

[10] We assume that phosphorus constitutes 13.6% of microalgal ash (based on 10.5% biomass ash content[81]), and that leaching phosphorus from ash costs equal the price of phosphorus rock.[82] This leads to P recovery costs of $47.6/ton ash. Half of ash disposal cost from Buchheit is paid additionally.

Additional tax/property insurance (frac. CapEx)		0.025	[69]	0.007	[10]
Electricity sale price ($/kWh)		0.119	[83]	0.133	[49]
DAC plant (costs per plant)					
DAC plant rated capacity (ton/year)		6000		10000	
DAC plant cost ($)		5.71e6	[50] Climeworks (sf=0.85, modular)	7.49e6	[50] 15% improvement, in line with other DAC tech (sf=.85, modular)
Heat pump CapEx ($/kW thermal)		309	[84]	216	70% realistic
DAC raw labor ($/year)		2.23e5	[85] US case (sf=0.9, LT DAC is modular)	1.17e5	[85] Oman case (sf=0.95, LT DAC is modular)
DAC Maintenance ($/year)		1.33e5	[85] 2.3% CapEx implied[11]	1.74e5	[85] 2.3% CapEx implied
Adsorbent cost ($/ton)		45.7	[85]	26.1	[86] MOF sorbent projection
Additional tax/property insurance (frac. CapEx)		0.0155	[85] CapEx implied	0.01	[85]
Heat pump OpEx ($/kW thermal/year)		12.2	[84]	12.2	
PVs and batteries					
Panel CapEx ($/kW DC)	C_{PV}	808	[87] UAE auction value	606	[88] advanced 2050 value[12]
Panel OpEx ($/kW DC/year)		15.1	[89]	15.1	[89]
Battery CapEx ($/kWh capacity)	C_{bat}	334	[90]	189	[90] moderate 2050 value
Battery OpEx (frac. CapEx) [DACCS]		.04	[90] 2.5% Cole et al. + 1.5% given long project lifetime	.03	[90] 1% Cole et al. + 2% given long project lifetime
Battery OpEx (frac. CapEx) [mBB]		.044	extended lifetime	.033	extended lifetime
CO_2 transport and storage					
CO_2 pipeline CapEx ($/km/m)		1.41e6	[63]	8.32e5	[63]
CO_2 storage CapEx ($/(ton/year))		0	[62]	0	[62]
CO_2 pipeline maint. ($/km/year)		5286	[63]	5286	[63]
CO_2 injection/storage OpEx ($/ton)		8.31	[62] see note[13]	5.38	[62]
Burial Site					
Burial Site CapEx ($)		0	neglected	0	
Trucking cost ($/ton/km)	C_T	0.112	[92]	0.112	[92]
Excavation cost ($/m^3)	C_x	5.44	[93]	2.55	[93]
Salt cost ($/ton salt)	C_s	19.5	[94] 20% increase for delivery	16.3	[94] solar production from brine TEA
Geomembrane cost ($/m^2)	C_{geo}	8.15	see note[14]	8.15	
Burial monitoring cost ($/ton)	B_M	2.84	[91] GRI monitoring, reporting and validation and verification value for biomass burial	2.84	[91]

Table S3: Embodied emissions and radiative forcings

Parameter (units)	Symbol	Real. value	Realistic notes	Opt. value	Optimistic notes

[11] Used here due to the discrepancies in the definition of CapEx with Young et al. (1.5% maintenance and 1% property insurance are given.)

[12] Note that cost improvement is compatible with areal efficiency improvement as costs are measured in $/kW not $/m^2.

[13] The value used for storage costs from Heddle includes monitoring costs and is taken here to encompass monitoring, reporting and verification (MRV) for geological injection CO_2 storage. Note that estimates of such MRV costs are time averaged over long periods to give a cost per ton. They are quite speculative and can vary widely. Values from Heddle compare well with DACCS and tBECCS MRV costs from Mercer et al.[91] which also provides the numbers used for mBB MRV, but should nonetheless be considered as only preliminary.

[14] The geomembrane is assumed to be 4 mm thick.[95] Areal cost is calculated from an HDPE bulk price of $1050 per ton[96] and then doubled for sheet production, with an HDPE density of 0.97 ton/m^3.

Microalgal pond					
Algal pond construction emissions (ton CO_2(eq)/ha)	GHG_{pond}	263	[97]	263	[97]
Grey ammonia emissions (ton CO_2(eq)/ton NH_3)		3.04	[98]	3.04	[98]
Green ammonia emissions (ton CO_2(eq)/ton NH_3)		0	neglected	0	
DAP emission (ton CO_2(eq)/ton DAP)	GHG_P	1.4	[98]	1.4	[98]
Biomass energy plant					
Biomass power total emissions (g CO_2(eq)/kWh)		49	[99]	49	[99]
DAC plant					
DAC total emissions (ton CO_2(eq)/ton)	GHG_{DAC}	0.085	[100]	0.03	[100]
PVs and batteries					
PV production/transportation emissions (g CO_2(eq)/kWh)	GHG_{PV}	46	[101]	46	[101]
Battery production emissions (ton CO_2(eq)/MWh capacity)	GHG_{bat}	75	[102]	75	[102]
Burial site					
Trucking emissions (g CO_2(eq)/ton/km)		58.6	[103]	0	electrified, see note[15]
Salt production emissions (ton CO_2(eq)/ton salt)		0	solar power used	0	
Geomembrane production emissions (kg CO_2(eq)/m2)		7.37	[65]	4.27	[65] recycled HDPE
Excavation emissions (kg CO_2(eq)/m3)		1.01	[16]	0	electrified
Albedo					
Earth area (km²)	A_E	5.1e8		5.1e8	
Radiative efficiency of CO_2 (W/m²/ton)	k_{CO_2}	1.62e-12	[7] calculated as in Bright with atmospheric CO_2 at 423 ppm	1.62e-12	
Vacant land albedo	α_0	0.4	[106] representative desert	0.4	[106]
Raceway pond albedo	α_r	0.1	[106] representative water	0.1	[106]
PV albedo	α_{PV}	0	No reflectance assumed	0	

Section S9: Sensitivity and uncertainty analysis

Global sensitivity of the cost model to selected parameters was calculated according to equations (S12)–(S14) following the methodology of Kiparissides et al.:[107]

$$\hat{E_i} = \frac{\partial j}{\partial v_i} \cdot \frac{1}{v_{i,max} - v_{i,min}} \qquad (S12)$$

$$\hat{g_i} = \int_{\hat{H}^n} \hat{E_i^2}\, dv \qquad (S13)$$

$$\hat{G_i} = \frac{\hat{g_i}}{\sum_{i=1}^{n} \hat{g_i}} \qquad (S14)$$

where $\hat{E_i}$ is the local partial derivative for the cost model $j(v)$ for a specific parameter i, normalised by the possible range of that parameter conveyed through $v_{i,max}$ and $v_{i,min}$, $\hat{g_i}$ calculates the average of the square of the partial

15 Trucking and excavation are assumed to be electrified, but the electricity production is not incorporated into the system considered. Electricity cost is contained within cost of trucking/excavation.

16 Calculated from an energy requirement for excavation of 14 MJ/m³ (ref.[104]) with energy supplied by gasoline with an emission factor of 72.2 g CO_2(eq)/MJ.[105]

derivatives of parameter i over the entire n-dimensional parameter space $\widehat{H^n}$, and the G score $\widehat{G_i}$ indicates a normalized contribution of parameter i to the total sensitivity of the model. The integration in equation (S13) was approximated by quasi-Monte Carlo sampling of the parameter space with 2^{20} Sobol' sampled points for each configuration. For PV-mBB and mBECCS-DACCS regions of the parameter space produced divergent costs (zero or net negative removals). Accordingly, $\widehat{H^n}$ was restricted to regions with costs below \$5000/ton to provide sensitivity scores relevant to possibly viable collections of parameters.

Uncertainty for each configuration was calculated via a weighted numerical sampling ($N=2^{20}$) from the parameter spaces. For PV-DACCS, mBECCS-DACCS, and PV-mBB, n equals 11, 22, and 21 respectively. Weighted sampling was performed by defining the probability of selecting any point v in parameter space according to equation (S15):

$$p(v_i) = \begin{cases} \dfrac{1}{2(v_i^* - v_{i,min}^*)}, & v_i \in [v_{i,min}, v_i^*] \\ \dfrac{1}{2(v_{i,max}^* - v_i^*)}, & v_i \in (v_{i,min}, v_i^*] \end{cases} \tag{S15}$$

where v_i^* refers to the nominal value for a parameter i, thereby setting nominal values as median for each parameter. From this sampling, the probability density functions are generated, as seen in Figure 5a.

Section S10: Sensitivity analysis parameters

Parameters for sensitivity and uncertainty analysis were selected by one-at-a-time perturbation, contribution to LCOC and reported importance in literature. Minimum and maximum values for each parameter were chosen based on literature and are presented in table S5. When parameter bounding was not well-founded a range of 0.5-1.5x the nominal value was applied.

Table S5: Global sensitivity and uncertainty analysis parameters

Parameter (units)	Symbol	Min. value	Minimum notes	Max. value	Maximum notes	Base value
Microalgal pond						
Paddlewheel electricity demand (kWh/ha/day)	W_r	40	opt. value	75	sens. max. from Davis[10]	50
Algal raw productivity (g/m^2/day)	y	12	[108,109]	30	[11]	18
Raceway DIC concentration (mM)	δ_r	0.1	Laboratory microalgal growth	0.5	Operational constraint, section S30	0.3
Raceway pond water supply head loss (m)	h_p	1	See section S11	6	See section S11	3
Operating days per year	op	188	[110] lower bound in lit. assumes contamination	365	[10]	330
Biomass carbon frac.	f_C	0.4	[30]	0.6	[111] with nutrient starving	0.5
Biomass nutrient content (C:P ratio)	r_P	75	[34]	120	[34] see note[17]	86
Pond CapEx (\$/ha)	C_{pond}	5.37e4	90% Davis min.[10]	1.45e5	[10]	7.74e4
Settling pond CapEx (scalar of nominal)	C_{set}	0.5		1.5		1

[17] Note that in the sensitivity/uncertainty analysis the N:P ratio is kept constant at the nominal value of 11.8. Accordingly, the implied range of C:N ratio is 6.6 to 9.9 which is slightly higher than that generally observed by Geider and La Roche (C:N ratio generally varies less than C:P ratio). Nonetheless, this allows nutrient content uncertainty to be spanned with one term.

Solar drying CapEx ($/m^2)	C_{sd}	6.3	opt. value	138	[68] (sf=0.95)	55.7
P cost ($/ton DAP)	C^*_{DAP}	582	opt. value	1929	300% real. value, peak phosphorus event	643
N cost ($/ton green NH_3)	$C^*_{NH_3}$	500	opt. value	2096	[74]	1153
Pond const. emissions (scalar of nominal)	GHG_{pond}	0.5		1.5		1
DAP emissions (ton CO_2(eq)/ton DAP)	GHG_P	0.6	[98]	6.4	[98]	1.4
Biomass energy plant						
Total auxiliary electricity consumption (frac. gross)	ℓ_{Aux}	0.25	result of best current values	0.4	result of conservatism in W_{CPU}, W_{ASU} and W_{aux}	0.343
mBECCS gross elec. yield (MJ/kg biomass)	Ψ_G	4.37	$HHV = 16.8$, $\eta_{comb} = 0.265$	9.2	$HHV = 23$, $\eta_{comb} = 0.42$	8.0
BPP CapEx (scalar of nominal)	C_{BPP}	0.5		1.5		1
Net price of elec. surp./deficit ($/kWh)	C_E	0	curtailment	0.1		0.0278
DAC plant						
DAC total electricity req. (kWh/ton)	W_{DAC}	744	[50]	2015	[1]	2116
DAC capacity factor	cf_{DAC}	0.765	see section S11	0.905	see section S11	0.819
DAC plant CapEx ($/plant)	C_{DAC}	4.29e6	[50]	1.33e7	[50]	5.71e6
Adsorbent cost (scalar of nominal)	C_{ads}	0.5		1.5		1
DAC emissions (ton CO_2(eq)/ton)	GHG_{DAC}	0.3	[100]	0.11	[100]	0.085
PVs and batteries						
Average Insolation (kWh/m^2/day)	$\bar{I}$	3.5	[112]	6.5	[112]	4.65
PV efficiency (kWh DC/kWh ins.)	η_{DC}	0.16	[113]	0.33	[113]	0.22
Total battery/curtailment elec. loss frac. [PV-mBB]	$\ell_{\Psi,mBB}$	0.2	see section S11	0.6	see section S11	0.35
Panel CapEx ($/kW DC)	C_{PV}	700	[87]	1661	[87]	808
Battery CapEx ($/kWh capacity)	C_{bat}	230	[90] Cole min	518	[90] Cole max + 15% supply chain vulnerability	334
Solar power emissions (g CO_2(eq)/kWh)	GHG_{PV}	25	[88]	125	[88]	46
Battery production emissions (ton CO_2(eq)/MWh capacity)	GHG_{bat}	49	[102]	203	[114]	75
Burial site						
Salt mass req. (ton salt/ton biomass)	f_s	0.25	[21]	1		0.667
Decomposition (frac. biomass buried)	ℓ_D	0		0.1	geomembrane failure and water intrusion event	0

Section S11: PV-mBB and PV-DACCS configuration optimization

Certain values in the base technoeconomic model and the sensitivity analysis depend strongly on environmental data of the location where a configuration is built as that data relates to the specifics of design. A regional analysis in Egypt was conducted to identify prospective growth and burial sites based on various land use proxies, elevation, and soil type from which burial site distance and water pipeline head loss h_p were derived. PV-DACCS and PV-mBB underwent optimization to determine optimal equipment capacity based on environmental data. Specifically, PV-DACCS optimization derived optimal battery and DAC plant capacities for a PV array given local time resolved insolation throughout the year. PV-mBB optimization derived optimal battery capacity, PV capacity, and pond operating salinity s_r in order to minimize cost of net CDR given time resolved local insolation, evaporation rate Φ, and microalgal productivity y_0, mediated through effects on variable total electricity demand W_m and pipeline electricity losses W_p. Optimization tended to increase costs of PV-mBB and decrease those of PV-DACCS relative

to the base technoeconomic model. Optimization and spatially resolved analysis are not included here due to the inability of microalgal CDR strategies to compete with PV-DACCS even in baseline technoeconomics.

Section S12: Enhancement Factor Derivation

The asymptotic enhancement factor given in equation (30) was derived following the work of Olander et al.[115] We begin by defining the five relevant chemical species: $CO_2 = Z$, $HCO_3^- = X$, $CO_3^{2-} = V$, $OH^- = F$, and $H^+ = L$. From these, we have reactions (S16)–(S20) and, by assuming chemical equilibrium, their equilibrium constants (while these equilibrium constants are apparent equilibrium constants that do not incorporate ion activities, the actual molarity-based equilibrium constants used in analysis are temperature and salinity dependent empirical observations in seawater, thereby reflecting the thermodynamic equilibrium relationship):

$$\mathrm{H_2O} \leftrightharpoons \mathrm{H^+ + OH^-} \quad , \quad \mathrm{K_0 = FL} \tag{S16}$$

$$\mathrm{CO_{2(aq)} + OH^- \leftrightharpoons HCO_3^-} \quad , \quad \mathrm{K_1 = \frac{X}{ZF}} \tag{S17}$$

$$\mathrm{HCO_3^- + OH^- \leftrightharpoons CO_3^{2-}} \quad , \quad \mathrm{K_2 = \frac{V}{XF}} \tag{S18}$$

$$\mathrm{HCO_3^- + H^+ \leftrightharpoons CO_{2(aq)}} \quad , \quad \mathrm{K_3 = \frac{Z}{XL}} \tag{S19}$$

$$\mathrm{CO_3^{2-} + H^+ \leftrightharpoons HCO_3^-} \quad , \quad \mathrm{K_4 = \frac{X}{VL}} \tag{S20}$$

We derive the five following mass balance equations (S21)–(S25) for each chemical species throughout the depth of the liquid film (x) and over time (t). Forward and reverse reaction rates for a reaction with equilibrium constant K_i are represented by k_i and k_i' respectively.

$$D_Z \frac{\partial^2 Z}{\partial x^2} - k_1 ZF + k_1' X + k_3 XL - k_3' Z = \frac{\partial Z}{\partial t} \tag{S21}$$

$$D_F \frac{\partial^2 F}{\partial x^2} + k_0 - k_0' FL - k_1 ZF + k_1' X - k_2 ZF + k_2' V = \frac{\partial F}{\partial t} \tag{S22}$$

$$D_X \frac{\partial^2 X}{\partial x^2} + k_1 ZF - k_1' X - k_2 ZF + k_2' V - k_3 XL + k_3' Z + k_4 VL - k_4' X = \frac{\partial X}{\partial t} \tag{S23}$$

$$D_V \frac{\partial^2 V}{\partial x^2} + k_2 ZF - k_2' V - k_4 VL + k_4' X = \frac{\partial V}{\partial t} \tag{S24}$$

$$D_L \frac{\partial^2 L}{\partial x^2} + k_0 - k_0' FL - k_3 XL + k_3' Z - k_4 VL + k_4' X = \frac{\partial L}{\partial t} \tag{S25}$$

Addition of equations (S21), (S23), and (S24) represents DIC conservation in equation (S26), while addition of (S22), (S23), 2*(S24), and subtraction of (S25) represents total alkalinity (hydroxide functional group) conservation in equation (S27):

$$\frac{\partial^2 Z}{\partial x^2}+\frac{\partial^2 X}{\partial x^2}+\frac{\partial^2 V}{\partial x^2}=\frac{\partial}{\partial t}(Z+X+V) \quad (S26)$$

$$\frac{\partial^2 F}{\partial x^2}+\frac{\partial^2 X}{\partial x^2}+2\frac{\partial^2 V}{\partial x^2}-\frac{\partial^2 L}{\partial x^2}=\frac{\partial}{\partial t}(F+X+2V-L) \quad (S27)$$

Here we assume steady state and solve the differential equations in (S26) and (S27) to yield equations (S28) and (S29) respectively:

$$D_Z Z+D_X X+D_V V=w_1 x+w_2 \quad (S28)$$

$$D_F F+D_X X+2D_V V-D_L L=w_3 x+w_4 \quad (S29)$$

Our first three boundary conditions are those of Olander,[115] characteristic of thin film theory: namely, at the interface $Z(0)=Z_i$ and at the base of the film $Z(x_L)=Z_b$ and $F(x_L)=F_b$. Our third boundary condition is given in equation (S30) reflecting that the steady state assumption that total alkalinity (that is hydroxide plus bicarbonate, plus double the carbonate, minus the hydrogen ion concentrations) in the interface must remain constant as it has no volatile species which could allow for transfer across the film.

$$\int\left[\frac{\partial^2 F}{\partial x^2}+\frac{\partial^2 X}{\partial x^2}+2\frac{\partial^2 V}{\partial x^2}-\frac{\partial^2 L}{\partial x^2}\right]dx=0 \quad (S30)$$

which is solved to imply $w_3=0$.

Inserting the four boundary conditions into equations (S28) and (S29) and solving for L, V, and X via equations (S16)–(S20), we generate equations (S31)–(S34):

$$D_Z Z_i+D_X(K_1 Z_i F_i)+D_V(K_1 K_2 Z_i F_i^2)=w_2 \quad (S31)$$

$$D_Z Z_b+D_X(K_1 Z_b F_b)+D_V(K_1 K_2 Z_b F_b^2)=w_1 x_L+w_2 \quad (S32)$$

$$D_F F_i+D_X(K_1 Z_i F_i)+2D_V(K_1 K_2 Z_i F_i^2)-D_L\left(\frac{K_0}{F_i}\right)=w_4 \quad (S33)$$

$$D_F F_b+D_X(K_1 Z_b F_b)+2D_V(K_1 K_2 Z_b F_b^2)-D_L\left(\frac{K_0}{F_b}\right)=w_4 \quad (S34)$$

From this system of equations we can solve for the enhancement factor given that mass transfer is represented by $J=-w_1$, thereby yielding equation (30). The interfacial hydroxide concentration F_i is calculated as the lone positive root of the cubic in equation (S35):

$$(2D_V Z_i K_1 K_2)F_i^3-(D_F+D_X Z_i K_1)F_i^2+\left(\frac{D_L K_0}{F_b}-D_F F_b-D_X Z_b F_b K_1-2D_V Z_b F_b^2 K_1 K_2\right)F_i-D_L K_0=0 \quad (S35)$$

Section S13: Raceway $k_L a$

Calculation of the k_L value in the raceway pond was calculated based on the empirical correlation of Asadollahzadeh et al.[116] with pond depth and fluid velocity and then adjusted to salinity and temperature based on the observation that k_L values vary with the square of diffusivity.[117]

Section S14: PBR $k_L a$

Mass transfer rate in photobioreactors was calculated as the sum of mass transfer at the bubble interface and mass transfer at the head space. Different k_L values lead to different values of E requiring their separate treatment despite the same driving force. Bubble interface mass transfer calculation followed the methodology of Kawase et al.[118] Specifically we use the empirical relationship for $k_L a_{bub}$ (s^{-1}) calculation in equation (S36):

$$k_L a_{bub} = Sh \cdot \frac{D_Z}{d_c} \tag{S36}$$

where d_c is PBR internal diameter and Sh is the Sherwood calculated by equation (S37):

$$Sh = 12 \cdot c_4 \cdot \sqrt{\frac{1.07}{\pi}} \cdot Sc^{\left(\frac{1}{2}\right)} \cdot Re^{\left(\frac{3}{4}\right)} \cdot Fr^{\left(\frac{7}{60}\right)} \cdot Bo^{\left(\frac{3}{5}\right)} \tag{S37}$$

with c_4 as a unitless constant and Sc, Re, Fr, and Bo are the unitless Schmidt, Reynolds, Froude, and Bond numbers, calculated via equations (S38)–(S41) respectively:

$$Sc = \frac{\nu}{D_A} \tag{S38}$$

$$Re = \frac{\mathrm{d_c} \cdot \mathrm{u_{sf}}}{\nu} \tag{S39}$$

$$Fr = \frac{\mathrm{u_{sf}^2}}{\mathrm{d_c} \cdot \mathrm{g}} \tag{S40}$$

$$Bo = \frac{\mathrm{g} \cdot \mathrm{d_c^2} \cdot \rho}{\sigma} \tag{S41}$$

with ν as kinematic viscosity, d_c as PBR diameter, u_{sf} as superficial gas velocity, σ as surface tension, g as gravity, and ρ as media density. These parameters as well as those used in enhancement factor calculation are provided as functions of temperature and salinity when relevant with values at S=35 g/kg and T=30 °C given in table S5:

For use in calculating enhancement factor via equation (29), $k_{L(bub)}$ (m/s) is determined by equation (S42):

$$k_{L(bub)} = 2\left(\frac{D_Z}{\pi}\right)^{\left(\frac{1}{2}\right)} \cdot \left(\frac{u_{sf} \cdot g}{\nu}\right)^{\left(\frac{1}{4}\right)} \tag{S42}$$

from which the interfacial area can be calculated as the quotient of $k_L a$ from equation (S36), and k_L from equation (S42) with the column volume v_{col}.

Mass transfer at the head space is instead calculated with a value $k_{L(head)}$ by equations (S43) and (S44):

$$k_{L(head)} = \sqrt{D_Z \cdot s} \tag{S43}$$

$$s = \frac{6 \cdot u_{sf}}{d_b} \qquad (S44)$$

where s (s^{-1}) is the fractional rate of surface renewal from Danckwert's surface renewal theory[126] and d_b is average bubble diameter (m) in the column which, while theoretically approximated and implicit in the relationship given by Kawase is simply estimated by photography and visual inspection of bubble columns. Area for headspace mass transfer simply equals column cross sectional area.

Table S5: Enhancement factor and $k_L a$ calculation constants and functions		
Parameter	Value	Source/calculation
$D_Z(S,T)$	2.26e-9 m^2/s	[119]
$D_X(S,T)$	1.22e-9 m^2/s	[119]
$D_V(S,T)$	8.88e-8 m^2/s	[119]
$D_F(S,T)$	5.74e-9 m^2/s	Calculated from conductivity of pure water, adjusted for salinity based on Einstein-Nernst viscosity adjustment[120]
$D_L(S,T)$	9.88e-9 m^2/s	Calculated from conductivity of pure water, adjusted for salinity based on Einstein-Nernst viscosity adjustment[120]
$Z_i(S,T)$	1.10e-2 mol/m^3	[121]
$K_0(S,T)$	9.95e-8 mol^2/m^6	[122]
$K_1(S,T)$	1.64e4 m^3/mol	[122]
$k_1(S,T)$	2.58e0 $m^3/mol/s$	[123]
$K_2(S,T)$	1.53e1 m^3/mol	[122]
c_4	6.45e-2	Empirically determined by Kawase[118]
$\nu(S,T)$	8.41e-7 m^2/s	Empirical salinity and temperature correlation[124]
d_c	0.104 m	
u_{sf}	3.92e-3 m/s	From volumetric gas flow 2 L/min
$\sigma(S,T)$	7.21e-2 N/m	Empirical salinity and temperature correlation[124]
$\rho(S,T)$	1021.6 kg/m^3	Thermodynamic equation of seawater[125]
v_{col}	0.003 m^3	Initially, decreasing due to evaporation and sampling

Section S15: Measured pH and chemical equilibria

We determined DIC concentration in bubble column samples from a calibration curve of pH as a function of DIC concentration at constant total alkalinity. The curve was prepared using freshly prepared and standardized NaOH and $NaHCO_3$, kept without headspace to minimize gas-liquid or liquid-gas CO_2 mass transfer that would alter DIC concentration. Media was prepared as in the bubble column experiment (see Mass Transfer Prediction and Observation). However instead of supplying all alkalinity from NaOH, alkalinity was supplied from varying concentrations of NaOH and $NaHCO_3$ to yield a range of DIC concentrations from 0 to 4 mM while keeping a TA of 5 mM. After alkaline reagents were mixed into media, two minutes incubation was allowed for chemical equilibrium to be reached while minimizing mass transfer before pH was measured at 30 °C (as in the bubble column experiment). pH was measured for two distinct replicates of each DIC concentration. The calibration curve was then generated from a smoothed mapping of average pH to DIC to ensure bijectivity (necessary for determining a single DIC concentration for a given pH—indeed a given TA and DIC represent a unique pH value as below). The calibration curve is shown in Figure S2. The use of this calibration curve for DIC determination

relies on assumption of constant TA which is not quite born out—evaporation from the columns over the course of the 8-hour experiment leads to a modest increase in TA as well as all other chemical species by ~2% (TA measurement by titration at hours 0 and 8 agreed with the expected increase in concentration from measured evaporation). This slight underestimation in the observed mass transfer was neglected.

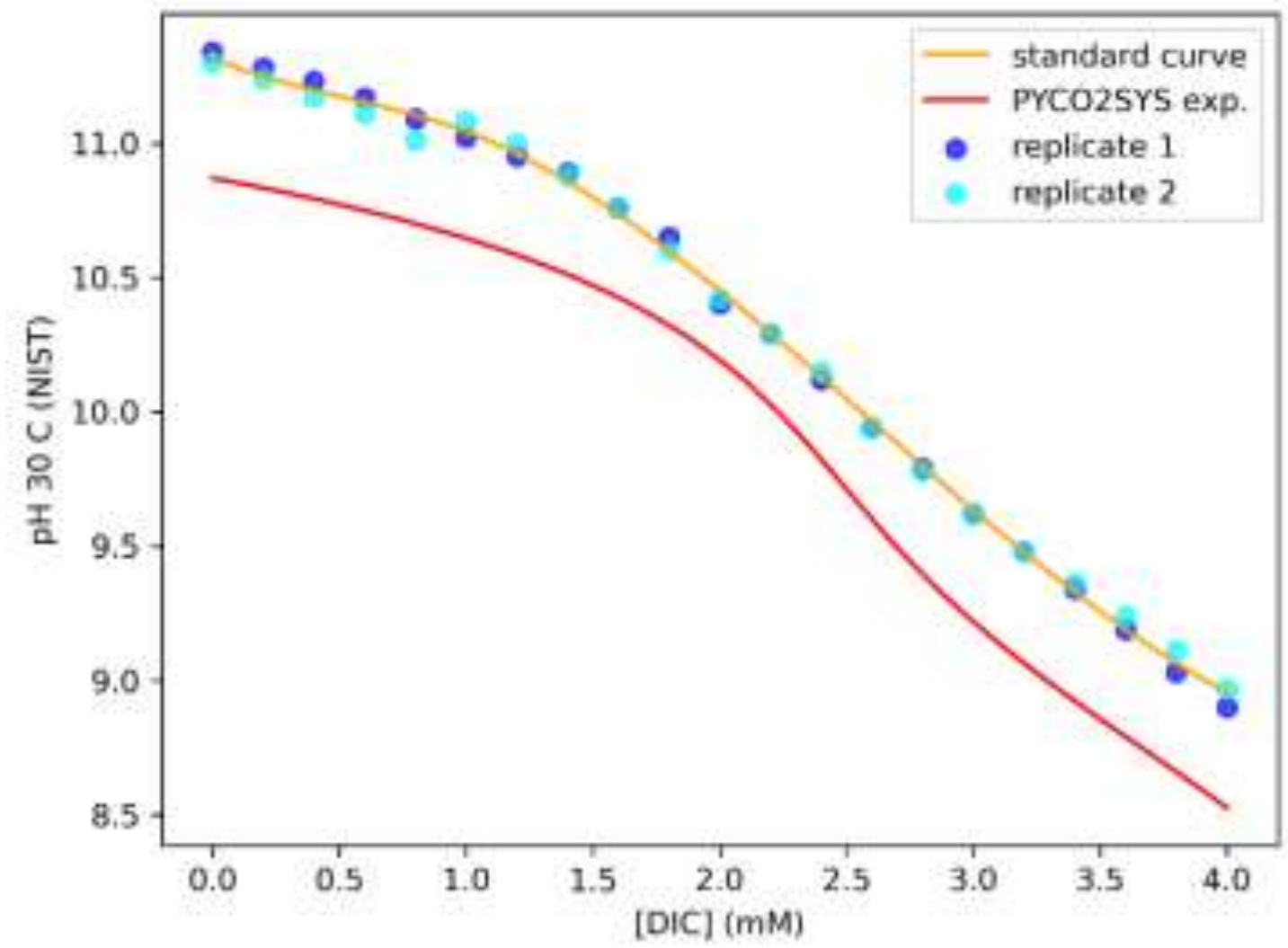

Figure S2: measured pH at different DIC concentrations with a constant TA of 5 mM, with smoothed decreasing standard curve and expected pH as calculated from TA and DIC given equilibrium relationships derived from relationships with temperature and salinity (of standard seawater composition).

While total DIC concentration is necessary for ascertaining the observed mass transfer, predicted mass transfer depends on the concentrations of CO_2 and OH^-, that is the values of Z_b and F_b at each time point for use in equations (27), (29), (30) and (S35) in conjunction with k_L and a values calculated in section S14 to permit calculation of enhancement factor and driving force. These values were determined using the package PYCO2SYS[127] with inputs of TA, media composition (namely concentrations of alkaline species being total phosphate and total borate), temperature, salinity, and DIC concentration (as determined from the calibration curve). Instead of using static TA, media composition, and salinity, these values were adjusted according to the increasing concentration following evaporation as discussed above though these have minimal effect on driving force (Z_b remains negligible compared to Z_i across the pH range tested), or on enhancement factor (F_b and F_i would need to increase severalfold to yield a value of E much greater than 1 for bubble interfaces while mass transfer across the head space is relatively small).

Knowing values of any two of the three parameters of DIC concentration, total alkalinity and pH is sufficient to determine the third with knowledge of chemical equilibria in solution. This principle allows packages like PYCO2SYS to determine the third such value from input of the other two. Thus, DIC concentration can in principle be determined in CO_2 mass transfer experiments simply from inputs of TA and pH without need of a calibration curve. Unfortunately, calculation of DIC based on salinity dependent carbonate, bicarbonate, borate, and phosphate equilibria (see table S5) and in PYCO2SYS was unsuccessful as negative DIC concentrations would be implied for the starting pH values and input TA. This may reflect the fact that empirical equilibrium relationships are based upon standard seawater which does not necessarily behave identically to our media (primarily NaCl due to the exclusion of divalent cations). Calculated pH as a function of DIC concentration at 5 mM TA from PYCO2SYS for the given media composition is also shown in Figure S2.

Supplementary results:

Section S16: Bubble column driving force consideration

Given the partial pressure of CO_2 in the atmosphere, hydrostatic pressure at the bubble column base from which bubbles emerge, and the volumetric gas flow rate, the total CO_2 input can be determined and compared to the observed mass transfer rate in figure 2f. CO_2 mass transfer is observed to consume 40.8% of the CO_2 in bubbles as they pass through the column which in turn will decrease partial pressure and the concentration Z_i, with effects on

driving force and chemical enhancement factor calculation via equations (27) and (30). However, the substantially higher variation in observed mass transfer as compared to calculated mass transfer means that a more properly resolved model with Z_i and following parameters varying with height in the column will not substantially affect correlation of prediction and observation. Calculated bubble surface area of 15.9*10^-3 m^2 (section S14) agreed reasonablg with direct measurements of area based on gas hold up via equation (S45):

$$a = \frac{6\epsilon_g}{d_b} \quad (S45)$$

where a is specific interfacial area (m^{-1}) and ϵ_g is gas hold up. ϵ_g was calculated from an average column height drop of 3.58 mm across the six PBRs when column bubbling was turned off. Combined with a d_b of 6 mm, a mass transfer area of 30.4 *10^-3 m^2 was calculated for a fluid volume of 3 L. More precise validation would measure bubble diameter distribution and use a Sauter mean and consider bubble ellipticity instead of assuming spherical bubbles (which was assumed to be a relatively poor assumption), but such refinements would not substantially help against high noise experimental observations (figure 2f).

The interface between the top of the bubble column and the head space provides an additional area of 7.9*10^-3 m^2 across which mass transfer could occur. $k_L a_{head}$ is much lower than $k_L a_{bub}$ and even the initial headspace enhancement factor of about 2 expected does not substantially affect the analysis.

Section S17: Mass transfer prediction in microalgal raceways

pH, DIC concentration, and mass transfer rate of chemically enhanced CO_2 mass transfer based on realistic raceway conditions are shown in figures S3a, S3b, and S3c respectively. One condition represents an absolute maximum where concentration of total ammonia and total phosphorus are neglected, and DIC is consumed instantaneously following mass transfer during daylight hours, while the other reflects more conservative assumptions where [total ammonia] = 0.8 mM, [total phosphate] = 0.05 mM, and minimum [DIC] is 0.3 mM. Media is assumed to have a total alkalinity of 2.5 mM consisting of 0.1 mM total borate, a temperature of 30 °C, salinity of 35 g/kg with representative seawater composition, raceway depth of 20 cm, and fluid velocity of 25 cm/s.

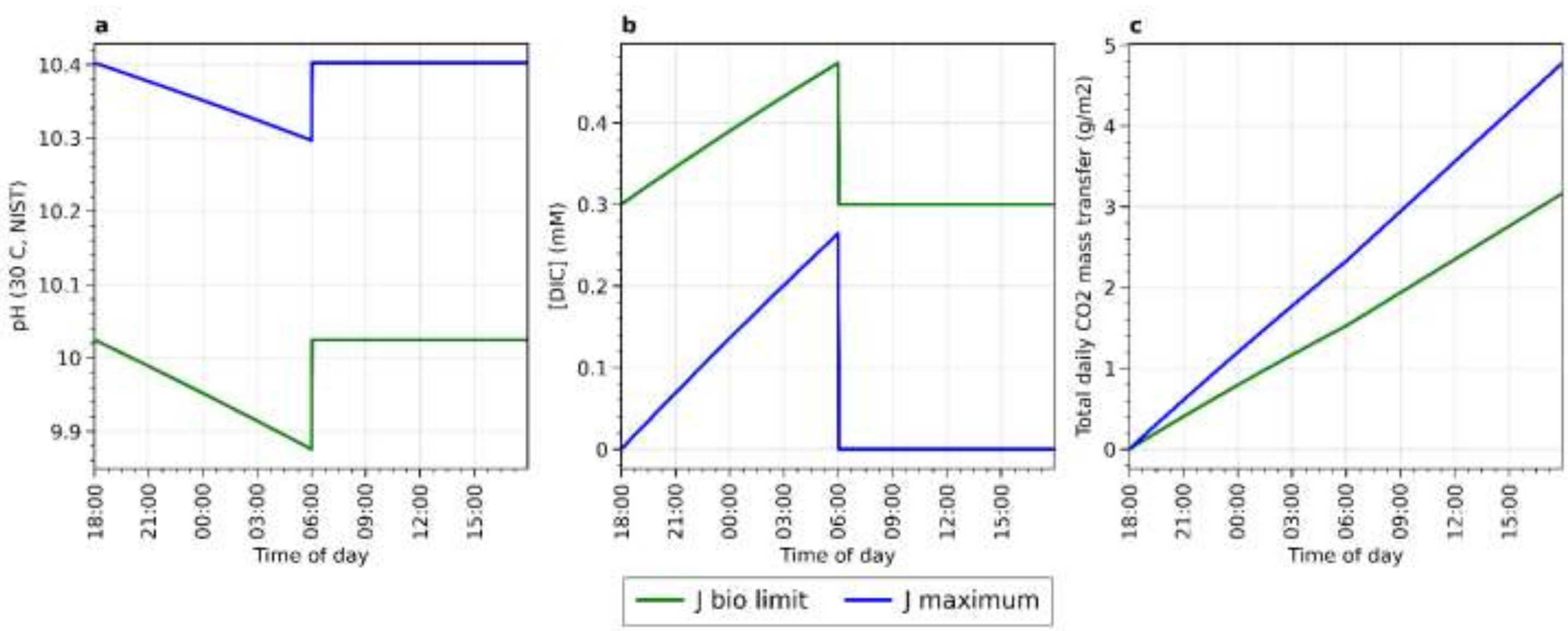

*Figure S3: modeled **a)** pH, **b)** DIC concentration, and **c)** cumulative mass transfer into raceways throughout the day. Microalgal growth and DIC consumption occurs only during daylit hours (6:00-18:00).*

Section S18: Burial site capacity

Burial site lifetime Cap_{BS} (years*ha growth/ha burial) is calculated according to equation (S46):

$$\mathrm{Cap_{BS}} = \frac{\mathrm{h_0 \cdot \kappa \cdot m_{bio}}}{\mathrm{Y}} \quad (S46)$$

Note that a factor of 10,000 is added to convert between m-ha from h_0 and Y to m^3 from m_{bio}. This equation yields a result of 341 and 171 years for the realistic and optimistic scenarios respectively. This result can otherwise be understood as the time required for burial area to equal growth area. Note that burial sites have capacity to store 20,000 and 22,000 tons of biomass corresponding to 28,000 and 41,000 tons of net CDR per hectare in realistic and optimistic scenarios respectively. This far exceeds the maximum observed organic matter content observed in nature of less than 700 tons (corresponding to no more than ~1,500 tons CO_2, even assuming 60% carbon content) per hectare.[128] Given that burial land is not necessarily consumed but could have other uses (as long as they do not introduce moisture) on the surface, burial footprint should not be considered as a substantial limitation.

Section S19: mBECCS solar and thermal drying comparison

Drying of biomass via thermal drying was compared for *marine* mBECCS design. The areal productivity of a solar drying mBECCS-DACCS configuration is calculated according to equations (S47) and (S48) (compare with equations (9) and (12)):

$$\mathrm{G^{T}_{mBECCS-DACCS}} = \kappa \cdot \mathrm{Y} \cdot \eta_{\mathrm{cc}} + \mathrm{Y} \cdot \frac{\mathrm{W_T - W_m}}{\mathrm{E_{DAC}}} \quad (S47)$$

$$\mathrm{W_T} = (\mathrm{HHV} - \mathrm{r_W} \cdot \mathrm{H_{ev}}) \cdot \eta_{\mathrm{comb}} \cdot (1 - \mathrm{W_{aux}}) - \mathrm{W_{ASU}} - \frac{\mathrm{W_{CPU}}}{\kappa} \quad (S48)$$

where W_T is the thermal drying net surplus electricity from the BECCS plant (k Wh/ton AFDW), equivalent to W_{BPP} in the solar drying case. Further, there is no solar drying area required in thermal drying mBECCS. This assumes that W_{aux} depends only on electricity production not on input chemical energy which is likely an underestimate and thus favorable towards thermal drying. Even so, in the realistic scenario $W_T < 0$ rendering the configuration impractical. In the optimistic scenario there is an electricity surplus, but it is less than 25% of that in mBECCS solar drying, which cannot be offset by the 3% increase in areal productivity via removal of the solar drying supplemental area requirement. Thermal drying mBB and the *atmospheric* option are similarly unfavorable in technoeconomics.

Section S20: mBB/mBECCS green and grey ammonia comparison

The use of ammonia produced with hydrogen from steam reforming of methane (grey) and that from renewable powered water electrolysis (green) were compared. Grey ammonia ($500 and $380/ton NH_3 in realistic and optimistic) is cheaper than green ammonia ($1153 or $500/ton NH_3) but grey ammonia has a substantial greenhouse gas footprint of production GHG_{NH3} (3.04 kg CO_2/kg NH_3), while this is much reduced for green ammonia (neglected herein). Though relative LCOC between the two ammonia sources for configurations (figure

S4b) vary quite substantially, green ammonia emerged as technoeconomically preferable. Generally, results are quite similar in the optimistic scenario while green ammonia offers a more substantial edge in the realistic scenario. This is likely due to the relatively high embodied emissions in the realistic scenario, to which any small increase (such as grey ammonia footprint) will dramatically increase LCOC. Note, however, that in a sufficiently broad analysis green ammonia would have its own area requirement and embodied emissions that have not been incorporated in this comparison.

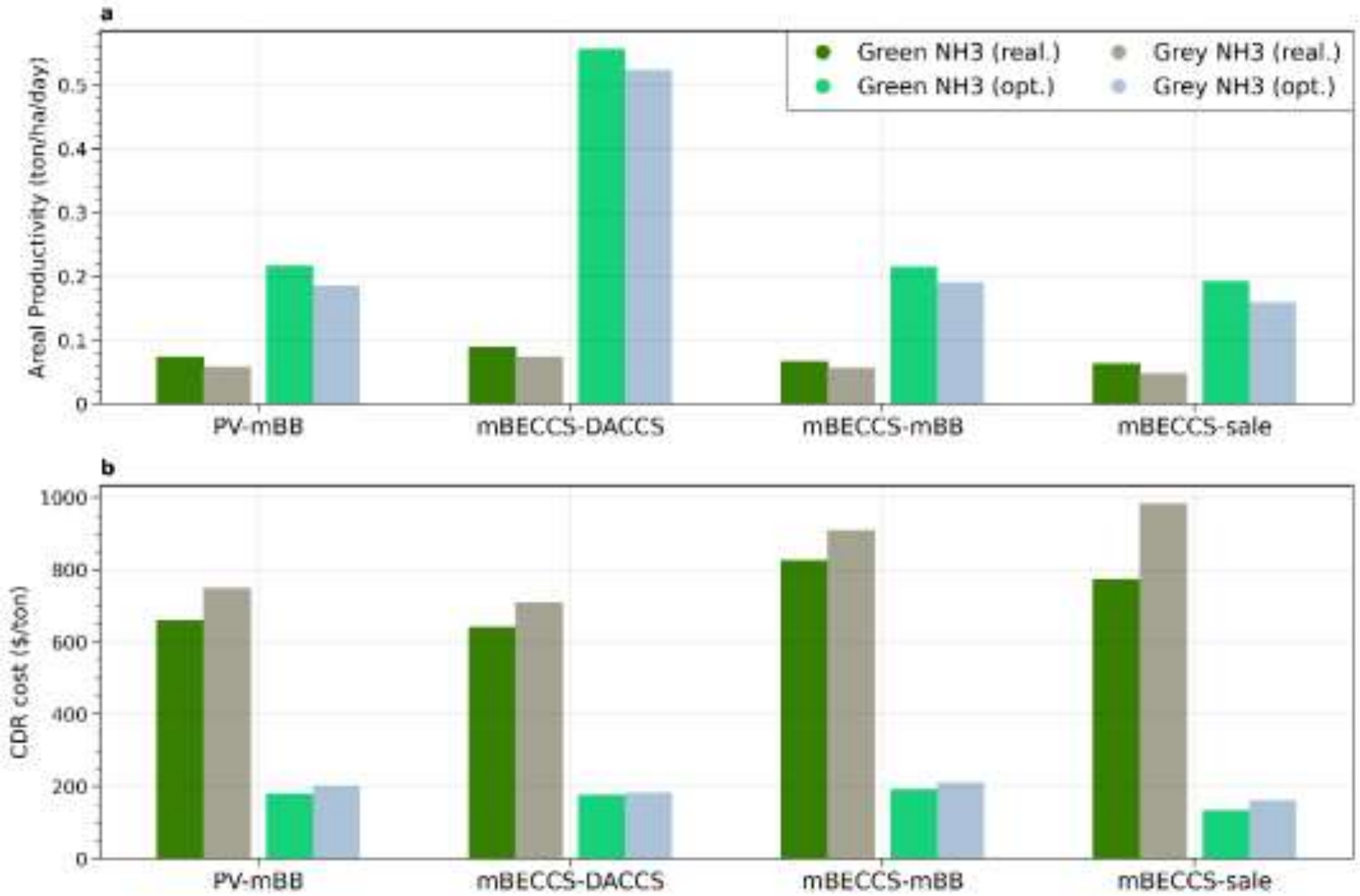

Figure S4: marine *option* ***a)*** *areal productivity and* ***b)*** *LCOC by configuration, scenario and choice of grey or green ammonia production.*

Section S21: Extended configuration and scenario areal productivities

Total areal productivities of mBECCS-mBB and mBECCS-sale configurations in both scenarios are shown in figure S4a. By design mBECCS-sale at 43 ton/ha/yr and 122 ton/ha/yr in the realistic and optimistic scenarios respectively has lower areal productivity than mBECCS-DACCS; in the realistic scenario mBECCS-mBB areal productivity is lower than PV-burial because the CO_2 loss in the CPU and embodied CO_2 of the mBECCS plant outweigh the PV areal footprint and embodied emissons, but in the optimistic scenario mBECCS-mBB areal productivity nearly converges with that of PV-mBB.

Figure S5a-c shows energy and carbon flows, while figure S5d-f shows em bodied emissions for the optimistic scenario for PV-DACCS, mBECCS-DACCS and PV-mBB configurations respectively. Meanwhile, mBECCS-mBB energy and carbon flows are shown in figure S6a-b, while its embodied emissions are shown in figure S6c-d for the realistic and optimistic scenarios respectively. Energy flows and embodied emissions for mBECCS-sale are not shown as they are identical to those for mBECCS-DACCS with the removal of solar drying land and DACCS energy consumption and associated embodied emissions.

Section S22: Extended configuration CDR costs

Net removal costs of mBECCS-mBB and mBECCS-sale are shown in figures S7a an d b respectively. mBECCS-sale has higher costs than both mBECCS-DACCS and PV-mBB in the realistic case but in the optimistic scenario costs are only $135/ton, the lowest value reported in this work. This result is discussed in section S33. Meanwhile,

mBECCS-mBB has higher costs than mBECCS-DACCS or PV-mBB in the realistic scenario while its costs drop to \$176/ton in the optimistic scenario. In mBECCS-mBB 77% and 25% of biomass is allotted to the mBECCS plant for electricity supply respectively.

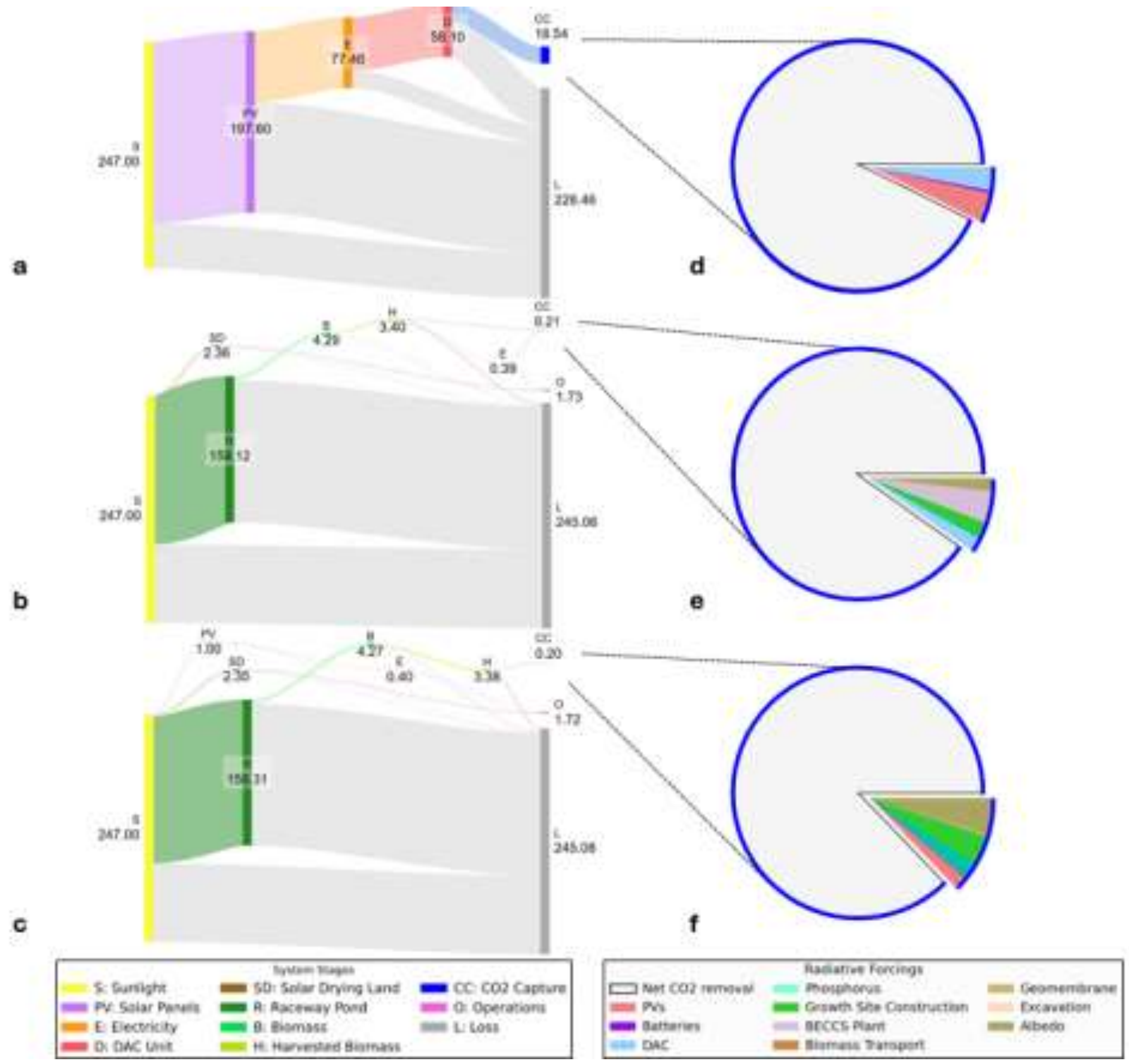

Figure S5: ***a-c)*** *optimistic scenario energy and carbon flows for PV-DACCS, mBECCS-DACCS, and PV-mBB respectively (in W/m^2, note that conversion from biomass chemical energy to carbon capture in c does not represented a process taken but conveys energy lost during the burial process by scaling captured carbon by the thermodynamic minimum to raise CO2 partial pressure to 150 ppm), and* ***b-d)*** *optimistic scenario embodied emissions for PV-DACCS, mBECCS-DACCS, and PV-mBB, respectively.*

Section S23: Microalgal growth, DAC, and total CDR cost comparison with literature

Figure S8a compares CDR costs for PV-DACCS, mBECCS-DACCS, and PV-mBB with those costs reported in literature. Reported realistic DACCS costs are substantially lower than those in literature[50] (due to our assumption of large-scale operations as implied by plant capacities and scaling factors which is generally not the case in realistic assumptions), while optimistic PV-DACCS is comparable to literature.[50,129] mBECCS and mBB CDR costs are higher than tBECCS values from literature in both scenarios;[130–132] finally, microalgal CDR costs are higher than CDR costs calculated from a microalgal injection for geological storage case in a microalgal biofuels study[133] where carbon supply is not so thoroughly interrogated, and all configuration costs provided here are higher than those associated with other promising CDR strategies such as enhanced weathering.[134] Figure S8b compares *marine* option microalgal growth costs (in units of tons AFDW) with those values reported in literature. Realistic costs are generally worse than those produced for large scale cultivation in literature.[10,133,135–137] This results from the demanding process of carbon supply from the ocean as compared to the relative low cost and

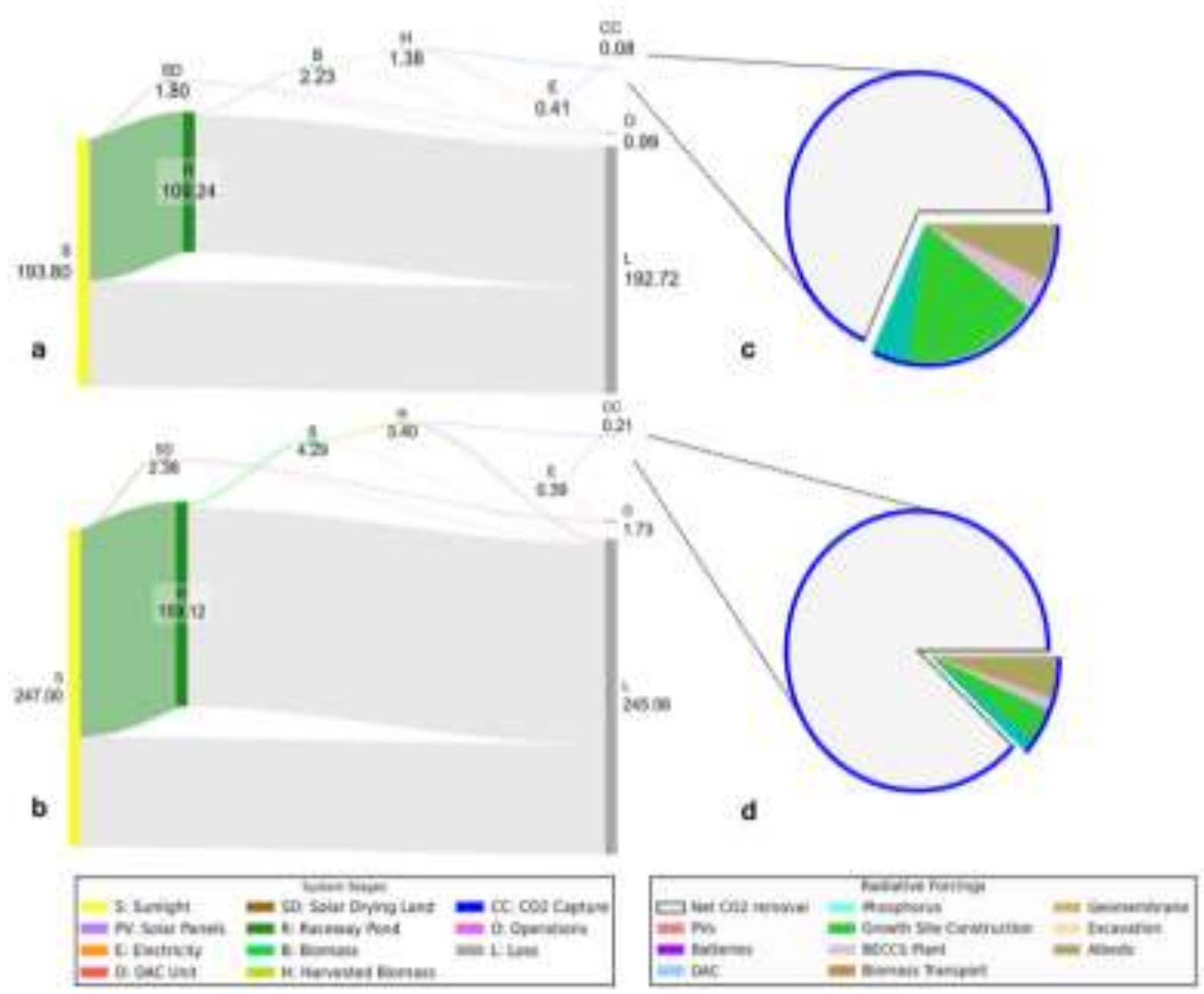

Figure S6: ***a)*** *realistic and* ***b)*** *optimistic scenario energy and carbon flows for mBECCS-mBB (W/m^2, see note above for figure S4 for B to CC conversion); and* ***c)*** *realistic and* ***d)*** *optimistic scenario embodied emissions for mBECCS-mBB configuration.*

low energy process of CO_2 sparging. It should be noted that this analysis, in its breadth produces lower costs for many components of microalgal growth than alternatives. For example, we account for the costs of electricity production explicitly (via PV arrays) instead of assuming an electricity cost from the grid as is done for nearly all sited microalgae or CDR analyses (excluding some DAC studies). The *atmospheric* option (not shown in figure S8b) produces even higher microalgal growth costs of $2540 and $864/ton AFDW in the realistic and optimistic scenarios respectively; beyond CDR application any sustainability motivation towards implementing such a strategy will likely be offset by the emissions embodied in the process.

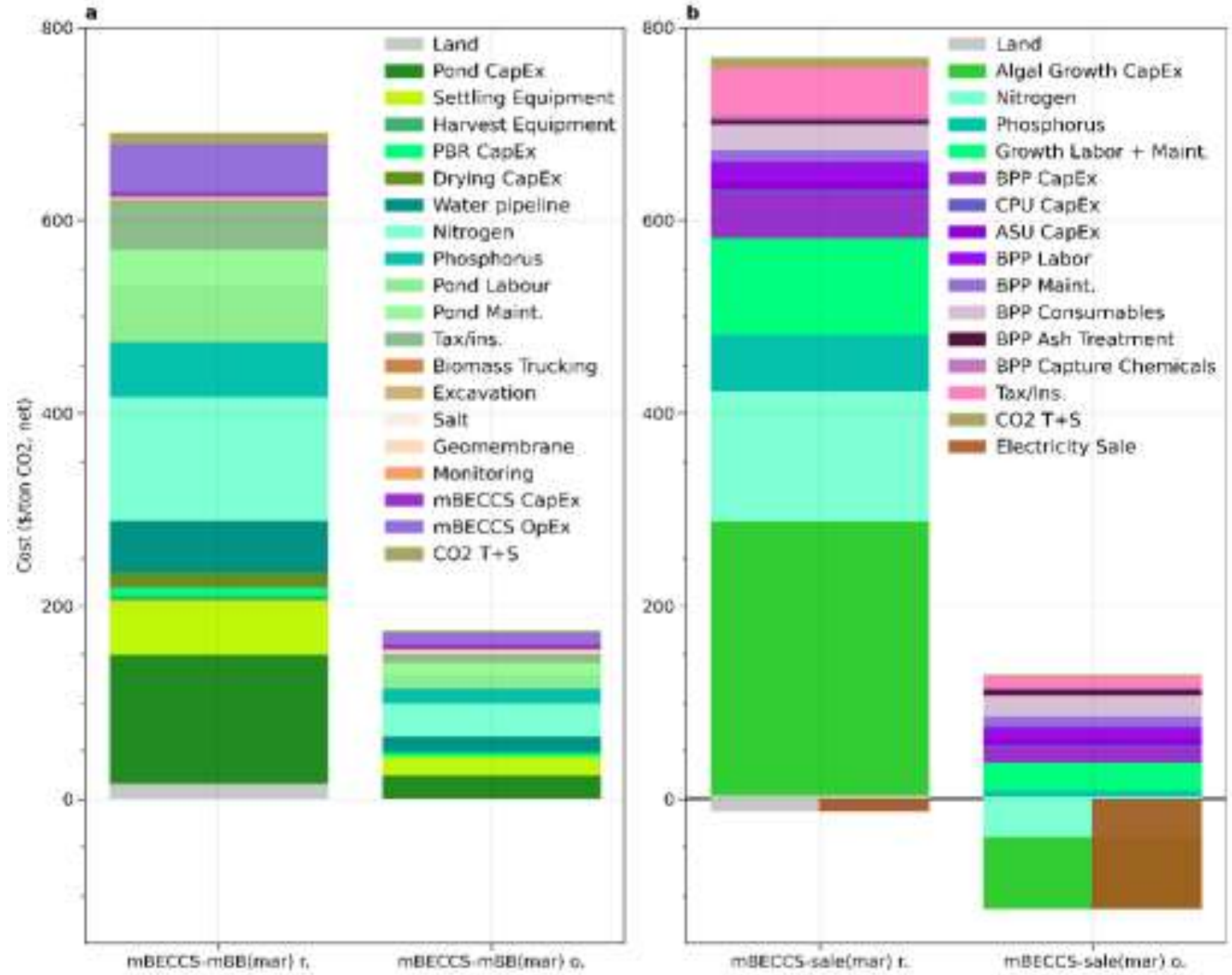

*Figure S7: Cost breakdown into components in each scenario for **a)** mBECCS-mBB and **b)***

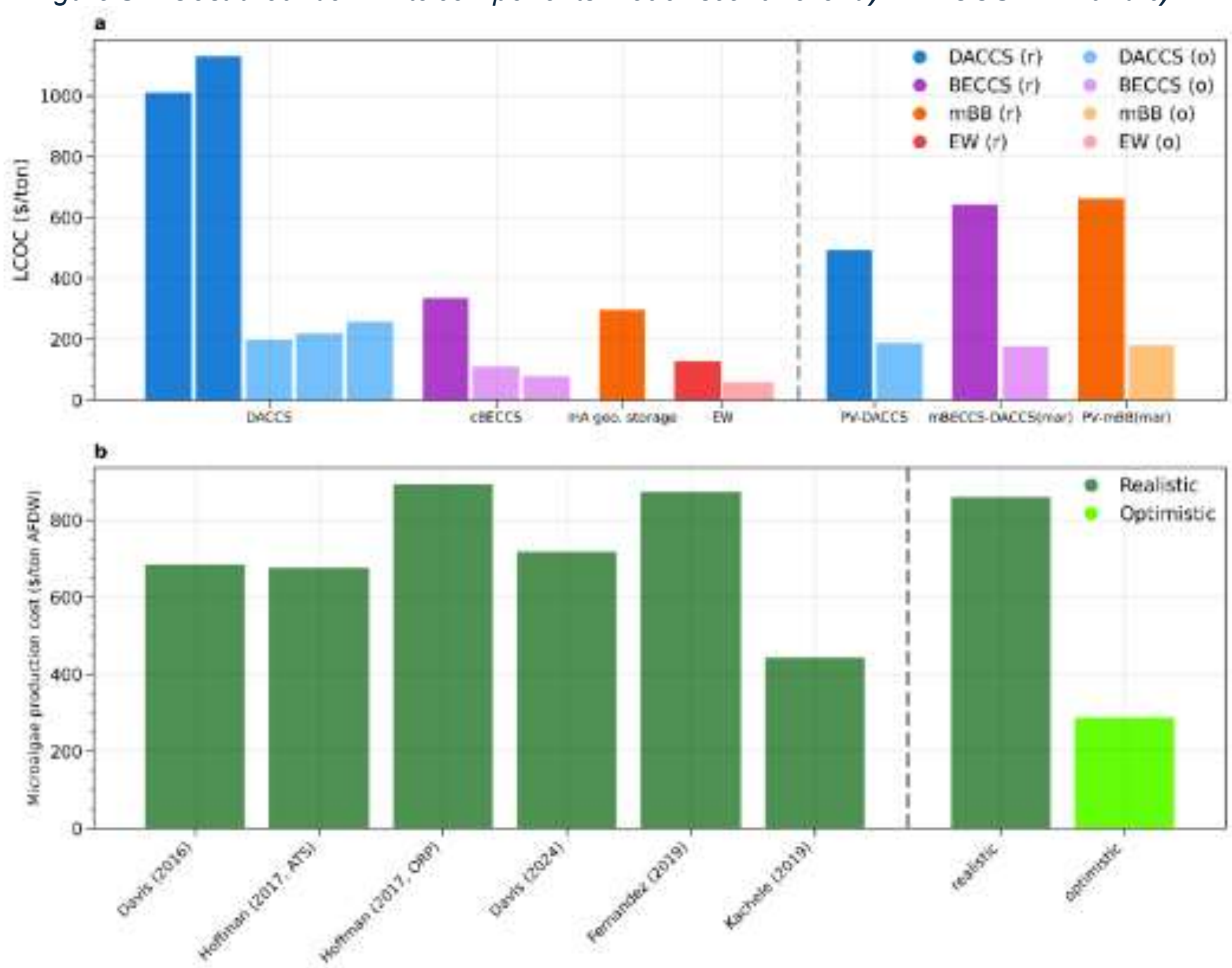

*Figure S8: **a)** CDR cost comparison between this work and literature with (from left to right) bars corresponding to DACCS: LT realistic, HT realistic, LT optimistic and HT optimistic DAC (Fasihi 2019), McQueen 2021; tBECCS: Bhave 2017, Emenike 2020, Sanchez 2016; microalgal geological storage: Kachele 2019; and enhanced weathering: current and future values from Muhlbauer 2024; and **b)** comparison of microalgal production costs from PV-mBB(mar) with values from literature.*

Section S24: *Atmospheric* option PV-mBB and mass transfer

Because biomass productivity is so low, LCOC in the *atmospheric* option is almost exclusively determined by y and the parameters that determine it, namely J_r. Thus, small shifts in outputs of the mass transfer model can cause big shifts in *atmospheric* option LCOC. Accordingly, figure

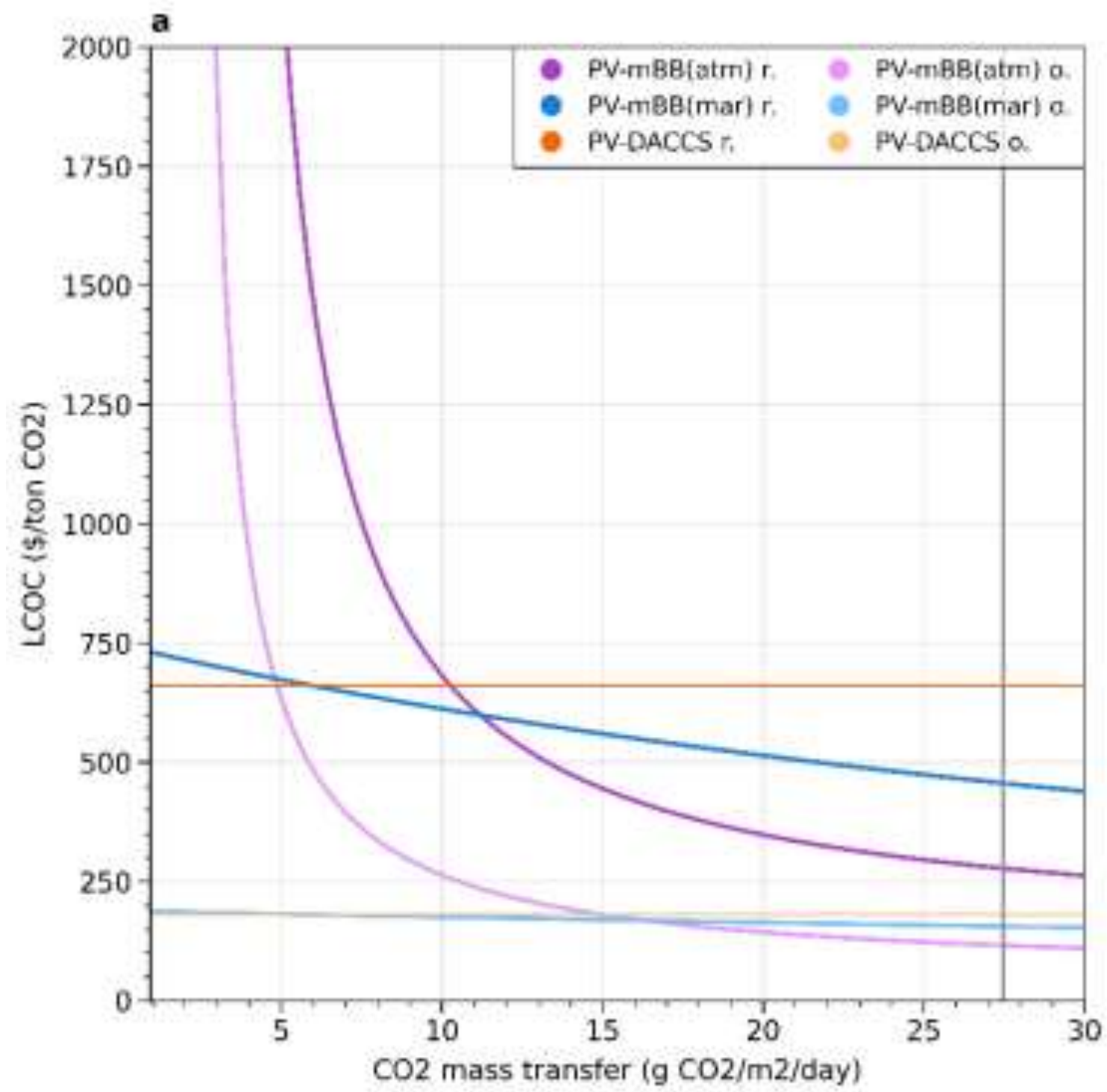

Figure S9: atmospheric *and* marine *option PV-mBB costs in realistic and optimistic scenarios as a function of mass transfer rate J_r as compared against PV-DACCS costs. The grey vertical line represents the microalgal cultivation target of 15 g AFDW/m^2/day.*

S9 shows PV-mBB(*atm*) costs in both scenarios. Certain experimental works[27,28] have suggested that higher mass transfer and microalgal growth rates may be possible, especially under substantially elevated alkalinity, though they could not be rectified with the theory presented here. Thus, the prospects for any further process that may enhance mass transfer rate (as discussed below) can be generally evaluated in terms of its CDR viability according to figure S9.

Section S25: Turbulence and J_r

By increasing turbulence of the mixing process, bulk liquid is more quickly brought into contact with air leading to an increase in k_L as mediated by an effective decrease in film thickness x_L. Thus, increasing turbulence to increase k_L will increase diffusion, and mass transfer in a diffusion limited regime. However, in a reaction rate limited regime, increasing k_L reduces the enhancement factor (see inverse dependence of Ha on k_L) leading to only very modest increase in mass transfer. Within reasonable values of k_L such behavior is observed here, meaning that increased turbulence is unlikely to relieve carbon limitation. See that J_{CO_2} only doubles with an increase to very high fluid velocity of 1 m/s in figure 2b.

Section S26: Air sparging and J_r

Most industrial algal growth relies on CO_2 bubbling. While CO_2 bubbling would defeat the CDR objective, the use of air bubbling into ponds could allow microalgal growth with atmospheric CO_2. However, energy required for such mass transfer is large. As an optimistic lower bound we assume that a perfect aerator can transfer all CO_2 from (micro)bubbles into ponds after bubbling from a depth of only 25 cm (typically much deeper sumps are required for such complete CO_2 transfer). The energy expenditure W_{bub} of compression (kWh/ha/day) is then calculated according to equation (S49):

$$W_{bub} = \frac{1}{\eta_{comp}} \cdot \frac{\gamma}{\gamma - 1} \cdot Q_{air} \cdot (p_0 + p_h) \cdot \left(\frac{p_0}{p_0 + p_h}\right)^{\left(\frac{\gamma-1}{\gamma}\right)} \qquad (S49)$$

where η_{comp} is compressor efficiency, γ is the adiabatic index of air, p_0 and p_h are atmospheric and hydrostatic (at a depth of 25 cm) pressure (Pa) respectively and Q_{air} is the air bubbling volumetric flow rate (m^3/ha/day). Values of η_{comp} of 0.8, $\gamma = 1.4$, p_0 and p_h of 1.01*10^5 and 2501 Pa respectively and Q_{air} sufficient to provide CO_2 for a biomass productivity of 15 g AFDW/m^2/day if supplied only through air bubbling would entail W_{bub} of 7,500 kWh/ha/day which is clearly prohibitively large.

Section S27: Air contactor and J_r

In DAC applications an air contactor is used, whereby large volumes of air are passed through a structure with very high, wetted internal surface area in order to facilitate rapid mass transfer. We considered passing raceway water through an air contactor as in Keith et al.[138] but found that, due to the low total alkalinity (and thereby capacity of seawater to store DIC), a large fraction of raceway volume (depending on depth) would need to be passed through the air contactor daily to maintain growth. This incurs prohibitive air contactor capital costs and pumping energy consumption far exceeding those of the *marine* option.

Section S28: Biofilms and J_r

Instead of growing microalgae planktonically in raceways, they could be grown in a wetted biofilm. Biofilms provide two opportunities for area enhancement, relative to a raceway pond: (1) at the macroscopic level a biofilm can be attached to a surface that is not parallel to the ground, allowing surface area and mass transfer on several inclined planes to far exceed that of the areal footprint,[139] and (2) at the microscopic level where roughness of the biofilm surface increases specific surface area.[140] However, cells within a biofilm often tend to be depleted of nutrient due to poor inter-film mass transfer. Additionally, any increased macroscopic area will likely lead to increased capital costs in proportion to the added specific surface area, as seen in elevated costs of biofilm relative raceway cultivation.[141] While increasing surface area and accordingly mass transfer J at a greater rater than capital costs may be possible, doing so by the factor of ~5 necessary for technoeconomic viability (figure S9) of *atmospheric* option CDR seems unlikely.

Section S29: Alkalinity enhancement and J_r

Artificial increase of alkalinity could increase chemical enhancement of mass transfer. However, increased alkalinity increases the risk of precipitation of various salts from solution, perhaps most immediately $Mg(OH)_2$. Indeed, at pH 10.3, substantial magnesium hydroxide precipitation is observed to lead to the flocculation of microalgae,[142] which in addition to the operational difficulties of precipitate formation and the loss of alkalinity addition, would make sustaining a culture at pH higher than 10.2 very difficult. In fact, even in the standard seawater pond case presented throughout, total DIC consumption raises pH to 10.5 or more (figure 2a) suggesting that further enhancement of alkalinity in microalgal ponds would not be feasible. Alternatively, the design of a second freshwater air contactor pond could be used to enhance mass transfer while avoiding precipitation. This, however, would face substantial difficulties. Figure 2c shows J_{CO_2} of ponds as a function of total alkalinity (far greater than that of seawater) and salinity. Practically, sufficient mass transfer could only be achieved at high alkalinities in freshwater. Even if freshwater must only be consumed at evaporation rate Φ, water supply via solar desalination—optimistically costed at \$3/m^3[143]—would alone present a cost of \$45,000/ha/year which at a y of 15 g AFDW/m2/day (entailing a very alkaliphilic strain) is equivalent to costs of \$448/ton CO_2. Though freshwater may be supplied without desalination, this is not a solution applicable to the gigaton scale of CDR demand or the objectives of this work.

Supplementary discussion:

Section S30: Settling ponds, auto-sedimentation, and flocculation

Microalgae generally have a higher density than water and will tend to naturally settle. However, due to their very small size and densities only slightly larger than that of seawater this process is very slow (<1 m/day). However, by aggregation of cells into particles much larger than the individual cell, they can sink at much faster rates, conducive to design of a settling pond. Certain microalgae naturally aggregate and can auto-flocculate or be used to flocculate other microalgae,[144] while other microalgae can be engineered to auto-flocculate such as the through the excretion of lipids that can allow for nonpolar interaction mediated cell sticking.[17] For microalgal harvest, Davis et al. suggest the use of settling ponds that can remove 90% of biomass into a sedimented phase with a density of 10 g/L from a culture density of 0.5 g AFDW/L in 4 hours.[10] To adapt this to a settling pond with a lower b_r, the collision rate will decrease in proportion to the square of the change in b_r, with proportional effects on sedimentation time even when collisions are highly effective at producing aggregates. Furthermore, flocculation tends towards a maximum efficiency even when flocculants are used well in excess of microalgae,[145] which suggests that loss rate in settling ponds ℓ_{set} may be expected to increase with decreasing b_r. The use of external flocculants may facilitate effective and timely settling, but given that much of the sediment biomass is resuspended in the raceways following DIC replenishment, flocculation would need to be reversible in addition to low cost for application to *marine* microalgal CDR. More research into auto-sedimentation and reversible flocculation of low density microalgal cultures is of great interest for large scale and low-cost media replacement with applications beyond the CDR system discussed herein.

Section S31: Microalgal CDR nutrient consumption

Nutrients (nitrogen and phosphorus) emerge as dominant factors in microalgal CDR economics, particularly in the optimistic scenario. Nitrogen can be supplied through proximately produced green ammonia which avoids adverse effects. Phosphorus, however, is a non-renewable mined resource, and the megaton scale phosphorus requirement implied by gigaton scale microalgal CDR might accelerate phosphorus limitation or peak phosphorus occurrence[133,146] and adversely affect fertilizer costs and thereby agricultural sustainability and economics.[147] Mitigation could entail alternative phosphorus sources, or limiting phosphorus consumption. Seawater in some locations is replete with nitrogen and phosphorus—enabling natural phytoplankton growth in these locations.[148] Net consumption of nutrients from seawater in certain locations such as river deltas with agricultural runoff may prevent harmful algal blooms, an ecosystem benefit, in addition to allowing for the possibility of recovery and sale of these nutrients, such as from bottom ash or hydrothermal treatment of biomass. However, broad consumption of marine nitrogen and phosphorus could adversely affect entire marine ecosystems. Furthermore, microalgal CDR additionality may be partially or entirely offset by impacts on the biological carbon pump.[149] Phosphorus consumption could be reduced by microalgal selection for high C:P ratio, P starvation before harvest, biomass separation based on P content, or P recovery via biomass thermal treatment. Phosphorus stored in buried biomass is not irreversibly lost and, should technology for effective phosphorus recovery from buried biomass composite without CO_2 reemission arise, phosphorus loss could be reversed, though overreliance on such an outcome should be avoided.

Section S32: Broad scope land use considerations

Configuration areal productivity results should be qualified by some consideration of broad-scope land requirement. While land costs of consumables production are implicitly incorporated into cost analyses via the single cost parameters used, the same does not occur for areal productivity. Broad scope land uses that are explicitly implied but not incorporated are for solar salt production and PVs required to generate power for trucking, excavation and electrolysis for green ammonia production. Improved carbon footprints, such as those for optimistic scenario DAC plants would likely also rely on similar green electricity for adsorbent production. Finally, non-sunlight consuming steps in any configuration and processes in related supply chains will reduce areal productivities. Such processes include (but are not limited to) phosphorus mining, burial sites, production of all consumables, production of construction materials, and any relocation of people necessary to provide labor for any configuration.

Section S33: mBECCS-sale and long term microalgal CDR prospects

Though this work is primarily grounded in a comparison of PV-mBB and mBECCS-DACCS as options for microalgal CDR—the mBECCS-sale configuration requires further discussion. This paper focuses on CDR strategies that do not rely on exporting power to grids or demand centers, because they tend to be more aligned with CDR objectives such as avoiding unfavorable land use changes (such marginal lands tend to lack grid connection) or burdening electricity grids. PV-mBB and mBECCS-DACCS readily meet these requirements. mBECCS-sale, which by definition requires electricity export, can respect these aims given intentional implementation. For example, to continue placing algal ponds on remote land, but supply the power generated by mBECCS to sites of demand (which will generally overlap with population centers, not algal ponds), long distance power transmission or biomass trucking from microalgal ponds to a BPP colocalized with power demand could be considered. There is ample current interest in and evidence for the viability of long-distance power transmission and distance limits are likely beyond distances demanded here,[150] and the semi-long distance trucking of biomass proved not to be a prohibitive cost in regional analysis of PV-mBB (see section S11).

We can further contextualize mBECCS-sale results by calculating a levelized cost of electricity (LCOE) to compare with tBECCS (terrestrial BECCS) results. LCOE ($/MWh) is calculated similarly to LCOC (equation (19)) according to equation (S50):

$$\mathrm{LCOE} = \frac{\mathrm{CapEx} \cdot \mathrm{crf} + \mathrm{OpEx}}{\mathrm{Y} \cdot (\mathrm{W_{BPP}} - \mathrm{W_m})} \tag{S50}$$

where subtraction of microalgal growth electricity requirement W_m from net BPP electricity yield W_{BPP} represents final electricity surplus for sale. This calculation yields an LCOE of $1,666/MWh in the realistic and $203/MWh in the optimistic scenario. Compare against values reported for tBECCS such as $291-351/MWh (oxy-BECCS, differences in biomass source, lower LCOE for other carbon capture strategies),[151] $235/MWh (not oxy, before savings with scale),[131] or $435/MWh (oxy, lower LCOE with other carbon capture strategies).[132] Differences between mBECCS and tBECCS can be attributed to the larger cost and energy requirement of microalgal growth and carbon supply as compared to woody biomass and agricultural waste. Inclusion of a carbon credit for CDR or an incentive for offsetting existing electricity production from fossil fuels may be applicable in certain contexts but will not fundamentally change the relationship of costs between mBECCS-sale and tBECCS. Figure S10 shows mBECCS-sale LCOC as a function of electricity revenue ($f_{prof} \cdot C_E$, after curtailment, etc. removed) and compares with PV-mBB costs in each scenario.

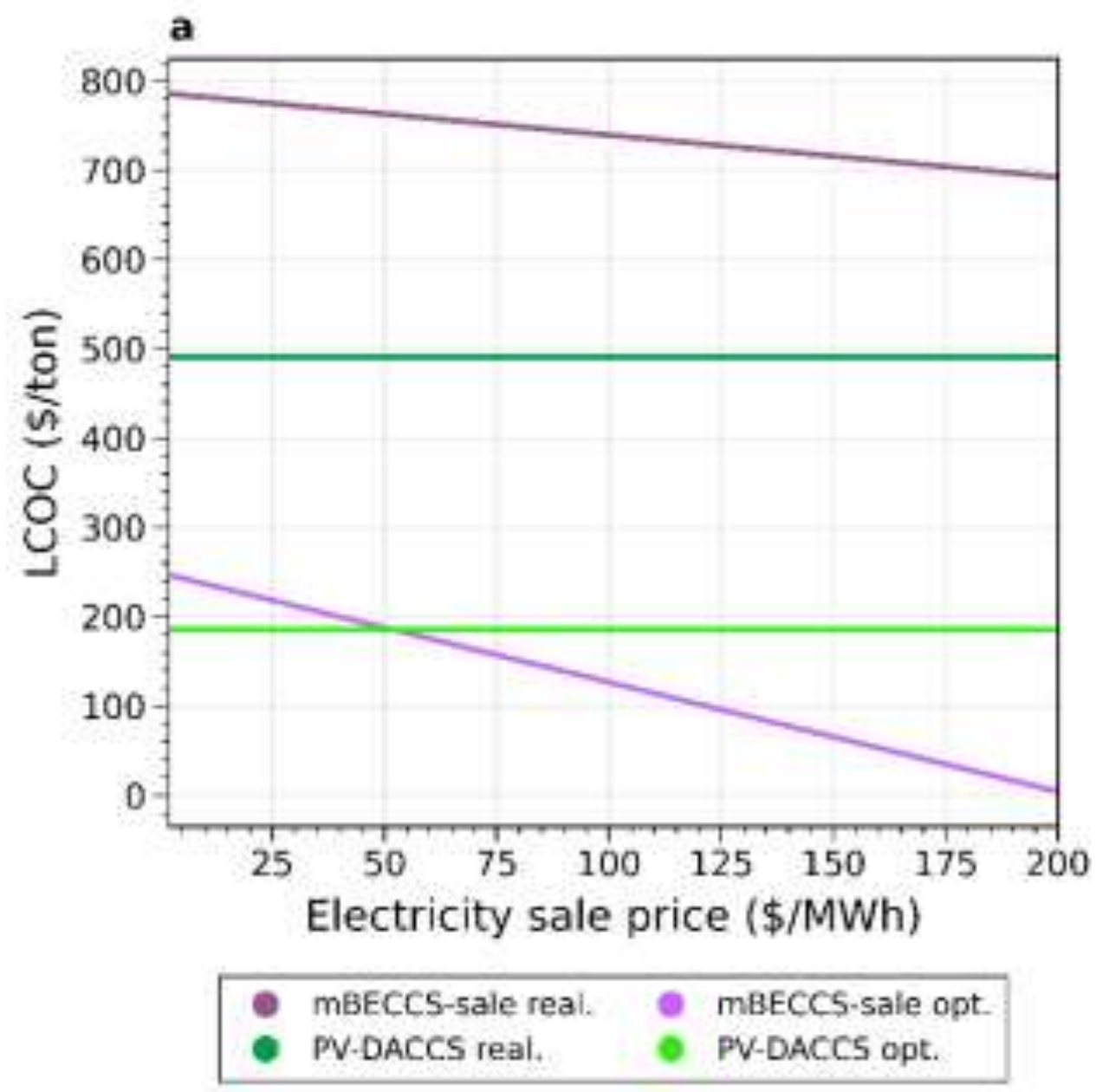

Figure S10: LCOC as a function of electricity sale price by configuration and scenario.

The optimistic LCOC of \$135/ton CO_2 (with electricity revenue) and LCOE of \$203/MWh (without CDR credit), if achieved, could substantially contribute to CDR. That said the improvements necessary to achieve such values are substantial, uncertain, and somewhat hard to justify given that realistic results for *marine* mBECCS-sale and all other microalgal CDR strategies are less compelling. Fortunately, the majority of technologies necessary for achieving the optimistic *marine* mBECCS-sale results will be of interest outside of the context of microalgal CDR. Research into BECCS technology, especially ASU and CPU performance, is rapidly proceeding. Similarly, microalgal technoeconomics are experiencing a new wave of interest and investment due to improvements in bioengineering for culture traits and co-production of high value products. Thus, only low culture density aggregation and media replacement emerges as a technology highly conducive to *marine* mBECCS-sale technoeconomics (and those of *marine* microalgal CDR more broadly) that has not yet received ample attention. Progress in these fields could render microalgal CDR a viable alternative to PV-DACCS especially in locations where land is abundant but capital to construct, operate, and provide specialized sorbents for DAC plants is not abundantly available.

Section S34: Contextualization of the optimistic scenario

While we endeavor in the realistic scenario to present configurations for which cost has been conservatively optimized given currently available technology, the optimistic scenario should be considered as a long-term target of configuration development, approaching a lower bound on costs. For each configuration, the optimistic scenario relies upon efficiencies only found at the lab scale or in specific conditions (e.g. MOF and zeolite-based DAC plant energy efficiencies,[3,152] areal growth rates of newly isolated cyanobacteria having limited growth data without CO_2 bubbling,[17] etc.), and on uncertain projected cost improvements (e.g. 2050 battery median cost estimates,[90] 2040 on-site green ammonia production cost[75]). To avoid double counting of improvements in the optimistic scenario, however, efficiency increases are considered first, before cost reductions are assessed more conservatively (assuming that new technologies may not achieve both cost reductions and efficiency approvements simultaneously), and carbon footprints are generally left equivalent to the realistic case except in those cases that carbon footprint may be linked to efficiency or cost improvement (e.g. DAC embodied carbon and sorbent performance/lifetime). Coherent bounding to improvements in cost, efficiency, and embodied carbon was explicitly ensured in the various optimization problems solved that yielded capacities, capacity factors and electricity loss rates. In the optimistic scenario (and to a lesser extent the realistic) certain processes and technologies employed have low levels of technological readiness (e.g. year-round industrial scale microalgal growth without CO_2 in either option, high throughput solar drying, extensive microalgal burial and monitoring), which would impact first-of-its-kind CDR performance and learning rates. Accordingly, we offer the optimistic scenario as an aspirational target for CDR performance once a configuration begins to be implemented at scale.

Section S35: Model limitations

Due to the spatially ambiguous nature of the technoeconomic analysis, all costs provided herein should be treated with caution. In attempting to design a CDR strategy, costing in dollars for each ton removed are not so important as dollars for each gigaton removed: scalability is obligatory. While such estimates can be (and have been) made for PV-DACCS due to its relatively limited susceptibility to environmental effects (other than insolation), they have not been for microalgal CDR strategies. The microalgal strategies are contingent upon ample use of vacant low elevation coastal land close to a biomass burial or CCS site; spatially resolved analysis of costs (section S11) suggest that microalgal CDR will not be able to achieve nearly the same scalability as PV-DACCS without manifold increases in LCOC which should hedge interpretation of optimistic microalgal CDR costs. CO_2 loss from pipelines and injection as well as the analogous of biomass throughout drying, transport, and burial processes have both been neglected as well as the embodied emissions of water and CO_2 pipelines. In this way, it is likely that if operated as framed in this work, removal costs for all configurations would be higher than those reported here, although we are not aware of any omissions that are likely to fundamentally alter conclusions.

Simultaneously, however, the configurations as modeled did not take advantage of several potential cost saving measures. These measures include variable pumping rate (so as to decrease salinity or increase DIC supply in times of curtailment), the production of algal byproducts, and equipment or byproduct sharing with other industries. CDR system design also provides opportunities for microalgal CDR. The PV-mBB system analyzed herein suffers from the curtailment of 35% of the electricity generated by its PV array in a cost optimized scenario due to the time offset between profiles of PV generation and the evaporation and harvest driven electricity demand. Similarly for PV-DACCS 25–27% of electricity generated is curtailed. Design of the PV-mBB system to use excess electricity to increase circulation and algal growth rates, drive on-site green ammonia production, or aid in the production of salt for burial could all decrease net removal costs. Indeed, on-site production of consumables emerges as a prospect that could decrease net removal costs through sharing of equipment, complementary usage of power sources, and reduction of embodied carbon footprint of consumable supply chains and transport.

References:

1. Deutz, S. & Bardow, A. Life-cycle assessment of an industrial direct air capture process based on temperature–vacuum swing adsorption. *Nat. Energy* **6**, 203–213 (2021).

2. Mahajan, S. & Lahtinen, M. Recent progress in metal-organic frameworks (MOFs) for CO2 capture at different pressures. *J. Environ. Chem. Eng.* **10**, 108930 (2022).

3. Wilson, S. M. W. The potential of direct air capture using adsorbents in cold climates. *iScience* **25**, 105564 (2022).

4. Rosa, L., L. Sanchez, D. & Mazzotti, M. Assessment of carbon dioxide removal potential via BECCS in a carbon-neutral Europe. *Energy Environ. Sci.* **14**, 3086–3097 (2021).

5. Shehzad, M. F., Ishaq, H. & Crawford, C. Offshore wind energy prospects for power-to-direct air capture and power-to-gas. *Int. J. Hydrog. Energy* **48**, 39216–39224 (2023).

6. Kreidenweis, U. *et al.* Afforestation to mitigate climate change: impacts on food prices under consideration of albedo effects. *Environ. Res. Lett.* **11**, 085001 (2016).

7. Bright, R. M. & Lund, M. T. CO_2-equivalence metrics for surface albedo change based on the radiative forcing concept: a critical review. *Atmospheric Chem. Phys.* **21**, 9887–9907 (2021).

8. Bright, R. M., Bogren, W., Bernier, P. & Astrup, R. Carbon-equivalent metrics for albedo changes in land management contexts: relevance of the time dimension. *Ecol. Appl.* **26**, 1868–1880 (2016).

9. Joos, F. *et al.* Carbon dioxide and climate impulse response functions for the computation of greenhouse gas metrics: a multi-model analysis. *Atmospheric Chem. Phys.* **13**, 2793–2825 (2013).

10. Davis, R. *et al. Process Design and Economics for the Production of Algal Biomass: Algal Biomass Production in Open Pond Systems and Processing Through Dewatering for Downstream Conversion*. https://www.osti.gov/biblio/1239893 (2016) doi:10.2172/1239893.

11. Moheimani, N. R. & Borowitzka, M. A. The long-term culture of the coccolithophore Pleurochrysis carterae (Haptophyta) in outdoor raceway ponds. *J. Appl. Phycol.* **18**, 703–712 (2006).

12. Pfeifroth, U. *et al.* PAR: Surface Radiation Data Set - Heliosat (SARAH) - Edition 3. 12.3 TiB Satellite Application Facility on Climate Monitoring (CM SAF) https://doi.org/10.5676/EUM_SAF_CM/SARAH/V003 (2023).

13. Lu, W. & Tian, J. Water surface reflection characteristics and power generation of bifacial PV modules. *IOP Conf. Ser. Earth Environ. Sci.* **601**, 012047 (2020).

14. NOAA Solar Calculator. *Global Monitoring Laboratory* https://gml.noaa.gov/grad/solcalc/index.html.

15. Alavianghavanini, A., Moheimani, N. R. & Bahri, P. A. Process design and economic analysis for the production of microalgae from anaerobic digestates in open raceway ponds. *Sci. Total Environ.* **923**, 171554 (2024).

16. Melis, A. Solar energy conversion efficiencies in photosynthesis: Minimizing the chlorophyll antennae to maximize efficiency. *Plant Sci.* **177**, 272–280 (2009).

17. Long, B. *et al.* Machine learning-informed and synthetic biology-enabled semi-continuous algal cultivation to unleash renewable fuel productivity. *Nat. Commun.* **13**, 541 (2022).

18. Ruangsomboon, S., Dimak, J., Jongput, B., Wiwatanaratanabutr, I. & Kanyawongha, P. Outdoor open pond batch production of green microalga Botryococcus braunii for high hydrocarbon production: enhanced production with salinity. *Sci. Rep.* **10**, 2731 (2020).

19. Cunha, P. *et al.* Nannochloropsis oceanica Cultivation in Pilot-Scale Raceway Ponds—From Design to Cultivation. *Appl. Sci.* **10**, 1725 (2020).

20. Romagnoli, F., Weerasuriya Arachchige, A., Paoli, R., Feofilovs, M. & Ievina, B. Growth Kinetic Model for Microalgae Cultivation in Open Raceway Ponds: A System Dynamics Tool. *Environ. Clim. Technol.* **25**, 1317–1336 (2021).

21. Jovine, R. *et al. Methodology: Carbon Dioxide Removal Through Algal Biomass Burial*. https://www.ecoengineers.us/wp-content/uploads/2023/11/EcoEngineers-BrilliantPlanet-Methodology.pdf (2023).

22. Tiris, C., Tiris, M. & Dincer, I. Energy efficiency of a solar drying system. *Int. J. Energy Res.* **20**, 767–770 (1996).

23. Silva, J. P. S., Veloso, C. R. R., de Souza Barrozo, M. A. & Vieira, L. G. M. Indirect solar drying of *Spirulina platensis* and the effect of operating conditions on product quality. *Algal Res.* **60**, 102521 (2021).

24. Gregor, L. & Gruber, N. OceanSODA-ETHZ: a global gridded data set of the surface ocean carbonate system for seasonal to decadal studies of ocean acidification. *Earth Syst. Sci. Data* **13**, 777–808 (2021).

25. Chatsungnoen, T. & Chisti, Y. Harvesting microalgae by flocculation–sedimentation. *Algal Res.* **13**, 271–283 (2016).

26. Talling, J. F., Wood, R. B., Prosser, M. V. & Baxter, R. M. The upper limit of photosynthetic productivity by phytoplankton: evidence from Ethiopian soda lakes. *Freshw. Biol.* **3**, 53–76 (1973).

27. Vadlamani, A., Pendyala, B., Viamajala, S. & Varanasi, S. High Productivity Cultivation of Microalgae without Concentrated CO2 Input. *ACS Sustain. Chem. Eng.* **7**, 1933–1943 (2019).

28. Gao, S. *et al.* A newly isolated alkaliphilic cyanobacterium for biomass production with direct air CO2 capture. *J. CO2 Util.* **69**, 102399 (2023).

29. Indrayani, I., Moheimani, N. R. & Borowitzka, M. A. Long-term reliable culture of a halophilic diatom, Amphora sp. MUR258, in outdoor raceway ponds. *J. Appl. Phycol.* **31**, 2771–2778 (2019).

30. Halder, P. & Azad, A. K. Chapter 7 - Recent trends and challenges of algal biofuel conversion technologies. in *Advanced Biofuels* (eds Azad, A. K. & Rasul, M.) 167–179 (Woodhead Publishing, 2019). doi:10.1016/B978-0-08-102791-2.00007-6.

31. Magalhães, I. B., Pereira, A. S. A. de P., Silva, T. A. & Renato, N. dos S. Predicting the higher heating value of microalgae biomass based on proximate and ultimate analysis. *Algal Res.* **64**, 102677 (2022).

32. Hedges, J. I. *et al.* The biochemical and elemental compositions of marine plankton: A NMR perspective. *Mar. Chem.* **78**, 47–63 (2002).

33. Redfield, A. C. On the Proportions of Organic Derivatives in Sea Water and Their Relation to the Composition of Plankton. in *James Johnstone Memorial Volume* 176–192 (University Press of Liverpool, Liverpool, UK, 1934).

34. Geider, R. & and La Roche, J. Redfield revisited: variability of C:N:P in marine microalgae and its biochemical basis. *Eur. J. Phycol.* **37**, 1–17 (2002).

35. Béchet, Q., Sialve, B., Steyer, J.-P., Shilton, A. & Guieysse, B. Comparative assessment of evaporation models in algal ponds. *Algal Res.* **35**, 283–291 (2018).

36. Piepmeier, J. *et al.* RSS SMAP Salinity. NASA https://doi.org/10.5067/GWYQTF307Y9Y (2023).

37. ESIA Project Team. *Red Sea Dead Sea Water Conveyance Study*. https://www.eib.org/attachments/registers/82425040.pdf (2017).

38. Wei, X., Manovic, V. & Hanak, D. P. Techno-economic assessment of coal- or biomass-fired oxy-combustion power plants with supercritical carbon dioxide cycle. *Energy Convers. Manag.* **221**, 113143 (2020).

39. Yuan, Y., Wang, L., Zhuang, Y., Wu, Y. & Bi, X. Energy and Economic Assessment of Oxy-Fuel Combustion CO2 Capture in Coal-Fired Power Plants. *Energies* **17**, 4626 (2024).

40. Banaszkiewicz, T., Chorowski, M. & Gizicki, W. Comparative Analysis of Oxygen Production for Oxy-combustion Application. *Energy Procedia* **51**, 127–134 (2014).

41. Cai, R., Dou, J., Krzystowczyk, E., Richard, A. & Li, F. Chemical looping air separation with Sr0.8Ca0.2Fe0.9Co0.1O3-δ perovskite sorbent: Packed bed modeling, verification, and optimization. *Chem. Eng. J.* **429**, 132370 (2022).

42. Rao, A. B., Rubin, E. S. & Berkenpas, M. B. *OXYFUEL COMBUSTION BASED CO2 CAPTURE SYSTEMS IN PULVERIZED COAL PLANTS*. https://www.researchgate.net/publication/237775185_OXYFUEL_COMBUSTION_BASED_CO2_CAPTURE_SYSTEMS_IN_PULVERIZED_COAL_PLANTS (2005).

43. Cau, G., Tola, V., Ferrara, F., Porcu, A. & Pettinau, A. CO2-free coal-fired power generation by partial oxy-fuel and post-combustion CO2 capture: Techno-economic analysis. *Fuel* **214**, 423–435 (2018).

44. Jackson, S. & Brodal, E. A comparison of the energy consumption for CO2 compression process alternatives. *IOP Conf. Ser. Earth Environ. Sci.* **167**, 012031 (2018).

45. Kotowicz, J., Michalski, S. & Brzęczek, M. The Characteristics of a Modern Oxy-Fuel Power Plant. *Energies* **12**, 3374 (2019).

46. Ling, J. L. J., Yang, W., Park, H. S., Lee, H. E. & Lee, S. H. A comparative review on advanced biomass oxygen fuel combustion technologies for carbon capture and storage. *Energy* **284**, 128566 (2023).

47. Talei, S., Fozer, D., Varbanov, P. S., Szanyi, A. & Mizsey, P. Oxyfuel Combustion Makes Carbon Capture More Efficient. *ACS Omega* **9**, 3250–3261 (2024).

48. García-Freites, S., Gough, C. & Röder, M. The greenhouse gas removal potential of bioenergy with carbon capture and storage (BECCS) to support the UK's net-zero emission target. *Biomass Bioenergy* **151**, 106164 (2021).

49. Mac Dowell, N. & Fajardy, M. Inefficient power generation as an optimal route to negative emissions via BECCS? *Environ. Res. Lett.* **12**, 045004 (2017).

50. Fasihi, M., Efimova, O. & Breyer, C. Techno-economic assessment of CO2 direct air capture plants. *J. Clean. Prod.* **224**, 957–980 (2019).

51. Schlosser, F. *et al.* Large-scale heat pumps: Applications, performance, economic feasibility and industrial integration. *Renew. Sustain. Energy Rev.* **133**, 110219 (2020).

52. McQueen, N., Desmond, M. J., Socolow, R. H., Psarras, P. & Wilcox, J. Natural Gas vs. Electricity for Solvent-Based Direct Air Capture. *Front. Clim.* **2**, (2021).

53. Mohan, A. *et al.* Direct air capture integration with low-carbon heat: Process engineering and power system analysis. *Energy Convers. Manag.* **322**, 119136 (2024).

54. ModelE AR5 Simulations: Past Climate Change and Future Climate Predictions. https://data.giss.nasa.gov/modelE/ar5plots/srlocat.html.

55. Wild, M. *et al.* The cloud-free global energy balance and inferred cloud radiative effects: an assessment based on direct observations and climate models. *Clim. Dyn.* **52**, 4787–4812 (2019).

56. Maalouf, A., Okoroafor, T., Jehl, Z., Babu, V. & Resalati, S. A comprehensive review on life cycle assessment of commercial and emerging thin-film solar cell systems. *Renew. Sustain. Energy Rev.* **186**, 113652 (2023).

57. Alharbi, F. H. & Kais, S. Theoretical limits of photovoltaics efficiency and possible improvements by intuitive approaches learned from photosynthesis and quantum coherence. *Renew. Sustain. Energy Rev.* **43**, 1073–1089 (2015).

58. Yilmaz, S. & Dincer, F. Impact of inverter capacity on the performance in large-scale photovoltaic power plants – A case study for Gainesville, Florida. *Renew. Sustain. Energy Rev.* **79**, 15–23 (2017).

59. Gooding, J. L. Geologic perspective for carbon sequestration by woody biomass burial. *Sci. Technol. Energy Transit.* **78**, 17 (2023).

60. Weidner, T., Yang, A., Forster, F. & Hamm, M. W. Regional conditions shape the food-energy-land nexus of low-carbon indoor farming. *Nat. Food* **3**, 206–216 (2022).

61. Becattini, V. *et al.* Carbon dioxide capture, transport and storage supply chains: Optimal economic and environmental performance of infrastructure rollout. *Int. J. Greenh. Gas Control* **117**, 103635 (2022).

62. Smith, E. *et al.* The cost of CO2 transport and storage in global integrated assessment modeling. *Int. J. Greenh. Gas Control* **109**, 103367 (2021).

63. Heddle, G., Herzog, H. & Klett, M. *The Economics of CO2 Storage*. https://energy.mit.edu/wp-content/uploads/2003/08/MIT-LFEE-03-003-RP.pdf (2003).

64. Boscoe, F. P., Henry, K. A. & Zdeb, M. S. A Nationwide Comparison of Driving Distance Versus Straight-Line Distance to Hospitals. *Prof. Geogr. J. Assoc. Am. Geogr.* **64**, 10.1080/00330124.2011.583586 (2012).

65. Veolia Water. *Winnipeg Sewage Treatment Program: South End Plant*. https://legacy.winnipeg.ca/finance/findata/matmgt/documents/2012/682-2012/682-2012_appendix_h-wstp_south_end_plant_process_selection_report/appendix%207.pdf.

66. Waghmare, A. G. *et al.* Characterization of storage stability of microalgal biomass for its applications as protein feed ingredients in animal and aquafeeds. *Anim. Feed Sci. Technol.* **288**, 115323 (2022).

67. Elfeky, K. E. & Wang, Q. Techno-economic assessment and optimization of the performance of solar power tower plant in Egypt's climate conditions. *Energy Convers. Manag.* **280**, 116829 (2023).

68. Udomkun, P. *et al.* Review of solar dryers for agricultural products in Asia and Africa: An innovation landscape approach. *J. Environ. Manage.* **268**, 110730 (2020).

69. Buchheit, K. L., Lewis, E., Mahbubani, K. & Carlson, D. R. *Technoeconomic and Life Cycle Analysis for Bio-Energy with Carbon Capture and Storage (BECCS) Baseline*. https://www.osti.gov/biblio/1810056 (2021) doi:10.2172/1810056.

70. Brownlie, W. J. *et al.* Phosphorus price spikes: A wake-up call for phosphorus resilience. *Front. Sustain. Food Syst.* **7**, (2023).

71. US Department of Agriculture. Fertilizer Transportation Dashboard. https://agtransport.usda.gov/stories/s/Fertilizer-Transportation-Dashboard/dtqv-e4ux/.

72. Moosazadeh, M., Mansourimarand, A., Ajori, S., Taghikhani, V. & Yoo, C. Waste-to-Ammonia: A sustainable pathway for energy transition. *Renew. Sustain. Energy Rev.* **208**, 115012 (2025).

73. Burgess, J. Interactive: Ammonia price chart. *S&P Global Commodity Insights* https://www.spglobal.com/commodity-insights/en/news-research/latest-news/energy-transition/051023-interactive-ammonia-price-chart-natural-gas-feedstock-europe-usgc-black-sea (2025).

74. Campion, N., Nami, H., Swisher, P. R., Vang Hendriksen, P. & Münster, M. Techno-economic assessment of green ammonia production with different wind and solar potentials. *Renew. Sustain. Energy Rev.* **173**, 113057 (2023).

75. Fasihi, M., Weiss, R., Savolainen, J. & Breyer, C. Global potential of green ammonia based on hybrid PV-wind power plants. *Appl. Energy* **294**, 116170 (2021).

76. Al-Qayim, K., Nimmo, W. & Pourkashanian, M. Comparative techno-economic assessment of biomass and coal with CCS technologies in a pulverized combustion power plant in the United Kingdom. *Int. J. Greenh. Gas Control* **43**, 82–92 (2015).

77. Seo, S. B. *et al.* Techno-economic comparison between air-fired and oxy-fuel circulating fluidized bed power plants with ultra-supercritical cycle. *Energy* **233**, 121217 (2021).

78. Yan, L., Cao, Y. & He, B. Energy, exergy and economic analyses of a novel biomass fueled power plant with carbon capture and sequestration. *Sci. Total Environ.* **690**, 812–820 (2019).

79. Porter, R. T. J. *et al.* Cost and performance of some carbon capture technology options for producing different quality CO2 product streams. *Int. J. Greenh. Gas Control* **57**, 185–195 (2017).

80. Fu, C. & Gundersen, T. Techno-economic analysis of CO2 conditioning processes in a coal based oxy-combustion power plant. *Int. J. Greenh. Gas Control* **9**, 419–427 (2012).

81. Cruz, J. D. *et al.* Bioprospecting for industrially relevant exopolysaccharide-producing cyanobacteria under Portuguese simulated climate. *Sci. Rep.* **13**, 13561 (2023).

82. Walsh, M., Schenk, G. & Schmidt, S. Realising the circular phosphorus economy delivers for sustainable development goals. *Npj Sustain. Agric.* **1**, 1–15 (2023).

83. Fajardy, M. *et al.* The economics of bioenergy with carbon capture and storage (BECCS) deployment in a 1.5 °C or 2 °C world. *Glob. Environ. Change* **68**, 102262 (2021).

84. Cox, J., Belding, S., Campos, G. & Lowder, T. *High-Temperature Heat Pump Model Documentation and Case Studies*. (2023).

85. Young, J. *et al.* The cost of direct air capture and storage can be reduced via strategic deployment but is unlikely to fall below stated cost targets. *One Earth* **6**, 899–917 (2023).

86. Sinha, A., Darunte, L. A., Jones, C. W., Realff, M. J. & Kawajiri, Y. Systems Design and Economic Analysis of Direct Air Capture of CO2 through Temperature Vacuum Swing Adsorption Using MIL-101(Cr)-PEI-800 and mmen-Mg2(dobpdc) MOF Adsorbents. *Ind. Eng. Chem. Res.* **56**, 750–764 (2017).

87. Egli, F., Orgland, N., Taylor, M., Schmidt, T. S. & Steffen, B. Estimating the cost of capital for solar PV projects using auction results. *Energy Policy* **183**, 113849 (2023).

88. NREL. Utility-scale PV. *Anual Technology Baseline* https://public.tableau.com/views/2024CMTS/TechSummary?:embed=y&:toolbar=no&Technology=UtilityPV&:embed=y&:showVizHome=n&:bootstrapWhenNotified=y&:apiID=handler0 (2025).

89. Wiser, R. H., Bolinger, M. & Seel, J. *Benchmarking Utility-Scale PV Operational Expenses and Project Lifetimes: Results from a Survey of U.S. Solar Industry Professionals | Energy Markets & Policy*. https://emp.lbl.gov/publications/benchmarking-utility-scale-pv (2020).

90. Cole, W., Ramasamy, V. & Turan, M. Cost Projections for Utility-Scale Battery Storage: 2025 Update. https://doi.org/10.2172/2583471 (2025) doi:10.2172/2583471.

91. Mercer, L., Burke, J. & Rodway-Dyer, S. *Towards Improved Cost Estimates for Monitoring, Reporting and Verification of Carbon Dioxide Removal*. https://www.lse.ac.uk/granthaminstitute/publication/towards-improved-cost-estimates-for-monitoring-reporting-and-verification-of-carbon-dioxide-removal/ (2024).

92. Austin, D. *Pricing Freight Transport to Account for External Costs: Working Paper 2015-03*. https://ideas.repec.org//p/cbo/wpaper/50049.html (2015).

93. Marschalko, M. *et al.* Engineering-geological study of relationships between soil and rock workability, type and volume of excavated materials, and earthwork costs (six case studies). *Sci. Rep.* **13**, 5377 (2023).

94. Zhao, J., Cheng, H., Wang, X., Cheng, W. & Cheng, F. Experimental investigation and cost assessment of the salt production by solar assisted evaporation of saturated brine. *Chin. J. Chem. Eng.* **26**, 701–707 (2018).

95. Yablonovitch, E. & Deckman, H. W. Scalable, economical, and stable sequestration of agricultural fixed carbon. *Proc. Natl. Acad. Sci.* **120**, e2217695120 (2023).

96. HDPE price per ton 2023. *Statista* https://www.statista.com/statistics/1171074/price-high-density-polyethylene-forecast-globally/.

97. Bradley, T., Forbes, J. & Ingram-Hardwick, G. The potential of microalgae for large-scale carbon capture and storage: A Life Cycle Assessment based on real-world data. Preprint at https://doi.org/10.21203/rs.3.rs-3147220/v1 (2023).

98. Walling, E. & Vaneeckhaute, C. Greenhouse gas emissions from inorganic and organic fertilizer production and use: A review of emission factors and their variability. *J. Environ. Manage.* **276**, 111211 (2020).

99. Mann, M. K. & Spath, P. L. *A Life Cycle Comparison of Electricity from Biomass and Coal*. https://www.osti.gov/biblio/20001888 (1999).

100. Terlouw, T., Treyer, K., Bauer, C. & Mazzotti, M. Life Cycle Assessment of Direct Air Carbon Capture and Storage with Low-Carbon Energy Sources. *Environ. Sci. Technol.* **55**, 11397–11411 (2021).

101. LCA Harmonization Project. *Life Cycle Greenhouse Gas Emissions from Solar Photovoltaics*. https://docs.nrel.gov/docs/fy13osti/56487.pdf (2012).

102. Kallitsis, E. *et al.* Think global act local: The dependency of global lithium-ion battery emissions on production location and material sources. *J. Clean. Prod.* **449**, 141725 (2024).

103. Mulholland, E., Ragon, P.-L. & Rodriguez, F. *CO2 Emissions from Trucks in the European Union: An Analysis of the 2020 Reporting Period*. https://theicct.org/publication/hdv-co2-emissions-eu-2020-reporting-jul23/ (2023).

104. Devi, L. P. & Palaniappan, S. A study on energy use for excavation and transport of soil during building construction. *J. Clean. Prod.* **164**, 543–556 (2017).

105. Carbon Dioxide Emissions Coefficients. https://www.eia.gov/environment/emissions/co2_vol_mass.php (2024).

106. Riihelä, A., Jääskeläinen, E. & Kallio-Myers, V. Four decades of global surface albedo estimates in the third edition of the CM SAF cLoud, Albedo and surface Radiation (CLARA) climate data record. *Earth Syst. Sci. Data* **16**, 1007–1028 (2024).

107. Kiparissides, A., Kucherenko, S. S., Mantalaris, A. & Pistikopoulos, E. N. Global Sensitivity Analysis Challenges in Biological Systems Modeling. *Ind. Eng. Chem. Res.* **48**, 7168–7180 (2009).

108. Mohan, N., Rao, P. H., Boopathy, A. B., Rengasamy, R. & Chinnasamy, S. A sustainable process train for a marine microalga-mediated biomass production and CO2 capture: A pilot-scale cultivation of *Nannochloropsis salina* in open raceway ponds and harvesting through electropreciflocculation. *Renew. Energy* **173**, 263–272 (2021).

109. Koley, S., Mathimani, T., Bagchi, S. K., Sonkar, S. & Mallick, N. Microalgal biodiesel production at outdoor open and polyhouse raceway pond cultivations: A case study with *Scenedesmus accuminatus* using low-cost farm fertilizer medium. *Biomass Bioenergy* **120**, 156–165 (2019).

110. Quiroz, D., McGowen, J. & Quinn, J. C. Implications of pond reliability on the techno-economic and life cycle environmental impacts of algal biofuels. *J. Clean. Prod.* **469**, 143178 (2024).

111. Maltsev, Y., Kulikovskiy, M. & Maltseva, S. Nitrogen and phosphorus stress as a tool to induce lipid production in microalgae. *Microb. Cell Factories* **22**, 239 (2023).

112. Habte, A., Sengupta, M., Gueymard, C., Golnas, A. & Xie, Y. Long-term spatial and temporal solar resource variability over America using the NSRDB version 3 (1998–2017). *Renew. Sustain. Energy Rev.* **134**, 110285 (2020).

113. Manisha, Pinkey, Kumari, M., Sahdev, R. K. & Tiwari, S. A Review on Solar Photovoltaic System Efficiency Improving Technologies. *Appl. Sol. Energy* **58**, 54–75 (2022).

114. Rahman, M. M., Gemechu, E., Oni, A. O. & Kumar, A. The greenhouse gas emissions' footprint and net energy ratio of utility-scale electro-chemical energy storage systems. *Energy Convers. Manag.* **244**, 114497 (2021).

115. Olander, D. R. Simultaneous mass transfer and equilibrium chemical reaction. *AIChE J.* **6**, 233–239 (1960).

116. Asadollahzadeh, M. J., Ardjmand, M., Seafkordi, A. A. & Heydarian, S. M. Efficient storage and utilization of CO2 in open raceway ponds for cultivation of microalgae. *Korean J. Chem. Eng.* **31**, 1425–1432 (2014).

117. Takagaki, N., Kurose, R., Kimura, A. & Komori, S. Effect of Schmidt number on mass transfer across a sheared gas-liquid interface in a wind-driven turbulence. *Sci. Rep.* **6**, 37059 (2016).

118. Kawase, Y. & Moo-Young, M. Mathematical models for design of bioreactors: Applications of: Kolmogoroff's theory of isotropic turbulence. *Chem. Eng. J.* **43**, B19–B41 (1990).

119. Zeebe, R. E. On the molecular diffusion coefficients of dissolved CO2, HCO3-, and CO32-, and their dependence on isotopic mass. *Geochim. Cosmochim. Acta* **75**, 2483–2498 (2011).

120. Light, T. S., Licht, S., Bevilacqua, A. C. & Morash, K. R. The Fundamental Conductivity and Resistivity of Water. *Electrochem. Solid-State Lett.* **8**, E16 (2004).

121. Weiss, R. F. Carbon dioxide in water and seawater: the solubility of a non-ideal gas. *Mar. Chem.* **2**, 203–215 (1974).

122. Millero, F. J. Thermodynamics of the carbon dioxide system in the oceans. *Geochim. Cosmochim. Acta* **59**, 661–677 (1995).

123. Johnson, K. S. Carbon dioxide hydration and dehydration kinetics in seawater. *Limnol. Oceanogr.* **27**, 849–855 (1982).

124. Sharqawy, M. H., Lienhard, J. H. & Zubair, S. M. Thermophysical properties of seawater: a review of existing correlations and data. *Desalination Water Treat.* **16**, 354–380 (2010).

125. McDougall, T. J. & Barker, P. M. Thermodynamic Equation of SeaWater 2010 (TEOS-10). https://www.teos-10.org/ (2011).

126. Danckwerts, P. V. Significance of Liquid-Film Coefficients in Gas Absorption. *Ind. Eng. Chem.* **43**, 1460–1467 (1951).

127. Lewis, E., Wallace, D. & Allison, L. J. *Program Developed for CO{sub 2} System Calculations*. https://www.osti.gov/biblio/639712 (1998) doi:10.2172/639712.

128. Soepadmo, E. Tropical rain forests as carbon sinks. *Chemosphere* **27**, 1025–1039 (1993).

129. McQueen, N. *et al.* Cost Analysis of Direct Air Capture and Sequestration Coupled to Low-Carbon Thermal Energy in the United States. *Environ. Sci. Technol.* **54**, 7542–7551 (2020).

130. Emenike, O., Michailos, S., Hughes, K. J., Ingham, D. & Pourkashanian, M. Techno-economic and environmental assessment of BECCS in fuel generation for FT-fuel, bioSNG and OMEx. *Sustain. Energy Fuels* **5**, 3382–3402 (2021).

131. Sanchez, D. L. & Callaway, D. S. Optimal scale of carbon-negative energy facilities. *Appl. Energy* **170**, 437–444 (2016).

132. Bhave, A. *et al.* Screening and techno-economic assessment of biomass-based power generation with CCS technologies to meet 2050 CO2 targets. *Appl. Energy* **190**, 481–489 (2017).

133. Kächele, R., Nurkowski, D., Martin, J., Akroyd, J. & Kraft, M. An assessment of the viability of alternatives to biodiesel transport fuels. *Appl. Energy* **251**, 113363 (2019).

134. Mühlbauer, A., Keiner, D. & Breyer, C. Techno-economic insights and deployment prospects of permanent carbon dioxide sequestration in solid carbonates. *Energy Environ. Sci.* **17**, 8756–8775 (2024).

135. Hoffman, J., Pate, R. C., Drennen, T. & Quinn, J. C. Techno-economic assessment of open microalgae production systems. *Algal Res.* **23**, 51–57 (2017).

136. Davis, R., Coleman, A. & Badgett, A. *Chapter 7: Emerging Resources: Microalgae, Macroalgae, and Point-Source Carbon Dioxide Waste Streams*. https://www.osti.gov/biblio/2316174 (2024) doi:10.23720/BT2023/2316174.

137. Acién Fernández, F. G., Fernández Sevilla, J. M. & Molina Grima, E. Chapter 21 - Costs analysis of microalgae production. in *Biofuels from Algae (Second Edition)* (eds Pandey, A., Chang, J.-S., Soccol, C. R., Lee, D.-J. & Chisti, Y.) 551–566 (Elsevier, 2019). doi:10.1016/B978-0-444-64192-2.00021-4.

138. Holmes, G. & Keith, D. W. An air–liquid contactor for large-scale capture of CO2 from air. *Philos. Trans. R. Soc. Math. Phys. Eng. Sci.* **370**, 4380–4403 (2012).

139. Ennaceri, H., Ishika, T., Mkpuma, V. O. & Moheimani, N. R. Microalgal biofilms: Towards a sustainable biomass production. *Algal Res.* **72**, 103124 (2023).

140. Chen, H. *et al.* Mass transfer in heterogeneous biofilms: Key issues in biofilm reactors and AI-driven performance prediction. *Environ. Sci. Ecotechnology* **22**, 100480 (2024).

141. Xu, Y. *et al.* Life-cycle and economic assessments of microalgae biogas production in suspension and biofilm cultivation systems. *Bioresour. Technol.* **395**, 130381 (2024).

142. Vandamme, D., Beuckels, A., Markou, G., Foubert, I. & Muylaert, K. Reversible Flocculation of Microalgae using Magnesium Hydroxide. *BioEnergy Res.* **8**, 716–725 (2015).

143. Ali, E. S. *et al.* Cost analysis and performance investigation of solar-powered desalination system utilizing modified sodium polyacrylate adsorbent material. *Energy Convers. Manag. X* **20**, 100421 (2023).

144. Bakhtiari, H., Taghavi, L., Mirbagheri, S. A., Rajaee, T. & Ramezani, M. Effective harvesting of the microalgae Cyclotella via autoflocculation and bioflocculation. *Int. J. Environ. Sci. Technol.* **19**, 6421–6428 (2022).

145. Labeeuw, L. *et al.* A comprehensive analysis of an effective flocculation method for high quality microalgal biomass harvesting. *Sci. Total Environ.* **752**, 141708 (2021).

146. Rhodes, C. J. Peak phosphorus - peak food? The need to close the phosphorus cycle. *Sci. Prog.* **96**, 109–152 (2013).

147. Mogollón, J. M., Beusen, A. H. W., van Grinsven, H. J. M., Westhoek, H. & Bouwman, A. F. Future agricultural phosphorus demand according to the shared socioeconomic pathways. *Glob. Environ. Change* **50**, 149–163 (2018).

148. Ustick, L. J. *et al.* Metagenomic analysis reveals global-scale patterns of ocean nutrient limitation. *Science* **372**, 287–291 (2021).

149. Polimene, L., Sailley, S., Clark, D., Mitra, A. & Allen, J. I. Biological or microbial carbon pump? The role of phytoplankton stoichiometry in ocean carbon sequestration. *J. Plankton Res.* **39**, 180–186 (2017).

150. Humpert, C. Long distance transmission systems for the future electricity supply – Analysis of possibilities and restrictions. *Energy* **48**, 278–283 (2012).

151. Emenike, O. *et al.* Initial techno-economic screening of BECCS technologies in power generation for a range of biomass feedstock. *Sustain. Energy Technol. Assess.* **40**, 100743 (2020).

152. Wiegner, J. F., Grimm, A., Weimann, L. & Gazzani, M. Optimal Design and Operation of Solid Sorbent Direct Air Capture Processes at Varying Ambient Conditions. *Ind. Eng. Chem. Res.* **61**, 12649–12667 (2022).
